# High-performance alkaline water electrolyzers based on Ru-perturbed Cu nanoplatelets cathode

**DOI:** 10.1038/s41467-023-40319-5

**Published:** 2023-08-04

**Authors:** Yong Zuo, Sebastiano Bellani, Michele Ferri, Gabriele Saleh, Dipak V. Shinde, Marilena Isabella Zappia, Rosaria Brescia, Mirko Prato, Luca De Trizio, Ivan Infante, Francesco Bonaccorso, Liberato Manna

**Affiliations:** 1 Nanochemistry Department, Istituto Italiano di Tecnologia, Via Morego 30, 16163 Genova, Italy; 2 BeDimensional S.p.A, Via Lungotorrente Secca, 30R, 16163 Genova, Italy; 3 Electron Microscopy Facility, Istituto Italiano di Tecnologia, Via Morego 30, 16163 Genova, Italy; 4 Materials Characterization Facility, Istituto Italiano di Tecnologia, Via Morego 30, 16163 Genova, Italy; 5 BCMaterials, Basque Center for Materials, Applications, and Nanostructures, UPV/EHU Science Park, Leioa, 48940 Spain; 6 Ikerbasque Basque Foundation for Science, Bilbao, 48009 Spain; 7 Graphene Labs, Istituto Italiano di Tecnologia, Via Morego 30, 16163 Genova, Italy; 8 Present Address: National Physical Laboratory, Hampton Road, Teddington, TW11 0LW UK

**Keywords:** Energy science and technology, Materials for energy and catalysis, Electrocatalysis

## Abstract

Alkaline electrolyzers generally produce hydrogen at current densities below 0.5 A/cm^2^. Here, we design a cost-effective and robust cathode, consisting of electrodeposited Ru nanoparticles (mass loading ~ 53 µg/cm^2^) on vertically oriented Cu nanoplatelet arrays grown on metallic meshes. Such cathode is coupled with an anode based on stacked stainless steel meshes, which outperform NiFe hydroxide catalysts. Our electrolyzers exhibit current densities as high as 1 A/cm^2^ at 1.69 V and 3.6 A/cm^2^ at 2 V, reaching the performances of proton-exchange membrane electrolyzers. Also, our electrolyzers stably operate in continuous (1 A/cm^2^ for over 300 h) and intermittent modes. A total production cost of US$2.09/kg_H2_ is foreseen for a 1 MW plant (30-year lifetime) based on the proposed electrode technology, meeting the worldwide targets (US$2–2.5/kg_H2_). Hence, the use of a small amount of Ru in cathodes (~0.04 g_Ru_ per kW) is a promising strategy to solve the dichotomy between the capital and operational expenditures of conventional alkaline electrolyzers for high-throughput operation, while facing the scarcity issues of Pt-group metals.

## Abstract (web summary)

Achieving high-efficiency alkaline water electrolyzer operating at large current densities remains a critical challenge. Here the authors report Ru nanoparticle-perturbed Cu nanoplatelets as cathode for hydrogen evolution reaction coupled with stainless steel anode in alkaline electrolyzer with high performance, long-term stability and relatively low-capital expenditures.

## Introduction

Hydrogen has long been considered as the most sustainable alternative to fossil fuels to meet the climate neutrality^1^. However, the industrial production of hydrogen mainly relies on hydrocarbon fuel processing, such as steam methane reforming^2^, partial oxidation^3^ and coal gasification^4^, which release harmful gases, including CO and CO_2_^5^. Zero-carbon “green” hydrogen can be produced through water electrolysis driven by electricity originated from renewable energy sources (e.g., solar and wind)^6^. Despite the huge potential of water electrolysis, the industrial production cost of “green” hydrogen, mainly relying on alkaline electrolyzers (AELs) based on platinum group material (PGM)-free catalysts (e.g., Ni and Fe)^7^, is generally between US$4–5.5/kg_H2_ (depending on the electricity costs)^8^, which is not competitive with that of “grey” hydrogen produced from fossil fuels (e.g., ~US$2.5/kg_H2_ for hydrogen produced through steam methane reforming)^9^. Traditionally, AELs face several limitations that are associated with the following factors: (1) the sluggish kinetics of alkaline water reduction and oxidation processes^10^, which restrict the cell-level operation to a current density <0.5 A/cm^2^ to reach a satisfactory energy efficiency (>70% based on the hydrogen higher heating value -HHV-)^11^; (2) traditional diaphragms feature a moderate OH^-^ mobility that causes resistive losses, while their porosity requires a pressure control system to avoid the safety-related issues resulting from the possible crossover of gases^12^.

In this work, we demonstrate that the main limitations of AELs can be overcome by systematically designing cost-effective cathodes and anodes with state of the art-like performances for alkaline water splitting reactions and using the recently validated commercial diaphragms^13^ for building performance-robust and safe AELs. The proposed hydrogen evolution reaction (HER) catalyst consists of electrodeposited Ru nanoparticles (with mass loading as low as 53 µg/cm^2^) on vertically oriented Cu nanoplatelets arrays grown on a Ti mesh (TM), leading to a Ru@Cu-TM electrode. Theoretical calculations reveal that the deposited Ru nanoparticles perturb the Cu substrate and weaken the Cu-H bond, hence facilitating H_2_ adsorption-desorption. Meanwhile Ru and Ti-based species act as water dissociation sites, thus leading to high performance of Ru@Cu-TM towards alkaline HER. As anode for the oxygen evolution reaction (OER), we employed commercially available stacked stainless steel meshes (SSMs). In the AEL environment (30 wt% KOH at 80 °C), stacked SSMs outperformed the research benchmark anode consisting of amorphous NiFe hydroxide grown onto Ni foam (NiFe@NF anode) in terms of catalytic activity and stability. We selected Zirfon Perl UTP 220 as the porous composite diaphragm since it features a high ionic conductivity (resistance ≤0.1 Ω·cm^2^)^13^ and a low hydrogen crossover (anodic hydrogen content typically <2%, and even <0.2% at operating current density ≥ 0.5 A/cm^2^) up to an operating pressure of 20 bar^13^. In these conditions, the diaphragm does not show the significant diffusive hydrogen crossover flux commonly observed in ion-exchange membranes^13,14^. The as-designed AELs, coupling the Ru@Cu-TM cathode with a 5-stacked SSMs anode, reach energy efficiencies (based on the hydrogen HHV and neglecting energy consumption from auxiliary electronics and thermal energy input) of 86.9% and 73.4% at current densities of 1.0 A/cm^2^ and 3.6 A/cm^2^, respectively. Therefore, they compete with the most performant electrolyzer (EL) technologies, including PGM-based proton-exchange membrane (PEM)-ELs and anion-exchange membrane (AEM)-ELs.

The stability and reliability of the proposed AEL technology are proven by both continuous operation and an accelerated stress test (AST), showing the possibility to modernize current high-technology readiness level AELs for green H_2_ production plants. Meanwhile, a preliminary technoeconomic analysis (TEA) of a 1 MW AEL plant (30-year lifetime) based on our electrode technology estimates a total cost (including capital expenditures -CAPEX-) of green hydrogen of ~US$2.09/kg_H2_. Beyond matching the performance of the most efficient PEM- or AEM-ELs, our AELs do not require massive amounts of PGM-based catalysts (e.g., Pt for cathodes and IrO_2_ for anodes) and expensive Ti bipolar plates (in our case, Ni bipolar plate were used), as instead needed by PEM-ELs to withstand the corrosion processes occurring in acidic environments^15^. In particular, we prove that, in our case, Ru has a negligible impact on the overall CAPEX. Additionally, our AELs take advantage of commercially available Zirfon-type diaphragms^13^, avoiding the instability issues of AEM-ELs during operation caused by the OH^-^-induced degradation of AEMs and CO_2_-induced formation of (bi)carbonates that decrease the membrane conductivity^16^.

Hence, we prove in this work that our AEL technology is competitive with grey hydrogen production methods (~US$2.5/kg_H2_), satisfying the target green hydrogen cost set by the European Commission^17^ for the coming decade (<US$2.5/kg_H2_). Moreover, despite Ru availability issues (typical of PGMs), a preliminary calculation based on the Ru mass per unit of deployed AEL power (i.e., ~0.04 g_Ru_/kW_AEL_) and the overall global electrolysis power forecasted by IRENA’s Energy Scenarios indicates that Ru usage should not be the bottleneck for the large-scale deployment of this technology. The obtained values are promising to fulfil the more ambitious targets set by the U.S. Department of Energy (US$2/kg_H2_ by 2026)^18^, thus competing with fossil fuels and boosting the transition to a climate-neutral economy.

## Results and discussion

### Cathode development and electrode-level (three-electrode configuration) characterization

**Fig. 1:**
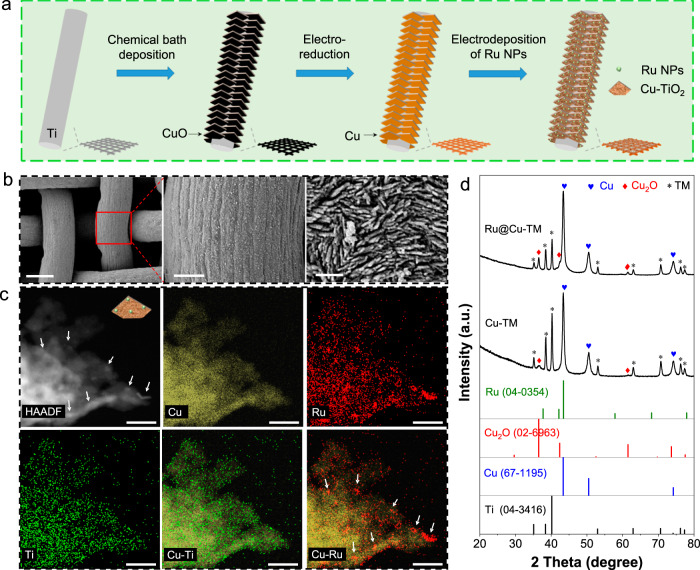
Morphological and compositional characterization of the Ru@Cu-TM cathode. **a** Fabrication scheme and **b** SEM images of the cathode with different magnifications. Scale bars: 200 μm (left), 50 μm (middle) and 1 μm (right). **c** HAADF-STEM micrograph of the fresh-prepared catalyst, and the corresponding EDS elemental maps for Cu, Ru, Ti, and the combination of Cu-Ti and Cu-Ru. White arrows indicate the Ru nanoparticles. Scale bars: 60 nm. **d** XRD pattern of the as-prepared Ru@Cu-TM cathode. The XRD pattern of Cu-TM is also shown for comparison, together with standard XRD patterns of Ti, Cu, Cu_2_O and Ru. a.u., arbitrary unit.

To develop efficient and low-cost HER electrocatalysts, we selected Ru as the cheapest material among PGMs with low Gibbs free energy for the hydrogen adsorption, as proven by efficient Ru-based catalysts for the HER reported in literature^19,20^. As for all PGM-based catalysts, the maximization of the Ru mass activity is paramount to reduce the impact on the overall costs of the electrodes^21^. Based on this rationale (check also Supplementary Note 1), we produced a cathode consisting of electrodeposited Ru nanoparticles (ca. 53 µg/cm^2^) on vertically oriented Cu nanoplatelets arrays grown on TM (Ru@Cu-TM) (see Scheme in Fig. 1a), in which the Cu nanoplatelets are expected to provide abundant surface for Ru loading, while acting as electrical pathways connecting the surface-deposited Ru nanoparticles to electrode current collector (TM). Contrary to previous works^19,20,22^, our Ru@Cu-TM features limited production costs and a greatly improved catalytic performance (e.g., 2.5 times the current density at an overpotential of 100 mV compared to ref. ^22^) achieved by systematically optimizing the Ru deposition step while upscaling the cathode size (up to 25 cm^2^) for practical applications.

Figure 1a and Supplementary Fig. 1 schematize the fabrication steps of the Ru@Cu-TM electrode: (i) growth of CuO nanoplatelets on TM through a chemical bath deposition; (ii) electroreduction of CuO nanoplatelets; (iii) electrodeposition of Ru nanoparticles (see details in Experimental Procedures). The Ru@Cu-TM cathode was first optimized by investigating either chronoamperometry (CA) protocols at various voltages or chronopotentiometry (CP) procedures for electrodeposition of Ru nanoparticles. The performances of the so-produced cathodes were assessed through linear sweep voltammetry (LSV) measurements (Supplementary Fig. 2a, b), as well as Tafel plots and electrochemical impedance spectroscopy (EIS) analyses (Supplementary Fig. 2c, d). Afterwards, the Ru nanoparticles electrodeposition was further optimized by tuning the concentration of the Ru precursor in the electrolyte to reduce the deposition time by a 7.3 factor (from 22 h to 3 h), without affecting the electrocatalytic performance (Supplementary Fig. 3). Besides, by such optimisation, we increased the efficiency of Ru deposition (i.e. increasing the effective amount of Ru precursor deposited from ca. 27% to over 50%), proving a cost-effective manufacturing protocol.

As shown by scanning electron microscopy (SEM) (Fig. 1b and Supplementary Fig. 4) and transmission electron microscopy (TEM) images (Supplementary Fig. 5), the as-developed Ru@Cu-TM cathode is composed of a three-dimensional nanostructured Cu nanoplatelet porous layer (thickness of *~*2 µm) vertically grown onto the TM current collector and decorated with Ru nanoparticles. The energy dispersive X-ray spectroscopy (EDS) elemental maps, coupled with SEM micrograph (Supplementary Fig. 6), and the high-angle annular dark-field scanning TEM (HAADF-STEM) imaging of catalyst fragments (Fig. 1c) indicate a uniform distribution of Cu, Ti and Ru over the catalytic layer, with Ru being present in the form of nanoparticles (Fig. 1c). High-resolution TEM (HRTEM) images evidence the presence of rutile TiO_2_ and Ru nanoparticles (Supplementary Fig. 7a, b), further confirmed by our XPS analyses (Supplementary Fig. 8), while inductively coupled plasma-optical emission spectroscopy (ICP-OES) measurements revealed that the average amount of electrodeposited Ru is 53 µg/cm^2^. The formation of TiO_2_ was explained considering that upon the formation of the Ru@Cu-TM, part of the Ti was etched and redeposited in the form of TiO_2_^23^. Indeed, ICP-OES measurements detected a Ti amount of ~34 ppb in the electrolyte after cathode preparation. The X-ray diffraction (XRD) pattern of the Ru@Cu-TM cathode is characterized by peaks that can be indexed with metallic Cu, Cu_2_O (caused by oxidation of air-exposed Cu), and metallic Ti of the TM substrate (Fig. 1d). Due to the low amount and small size of deposited Ru and TiO_2_ species, no XRD peak related to them were observed.

**Fig. 2:**
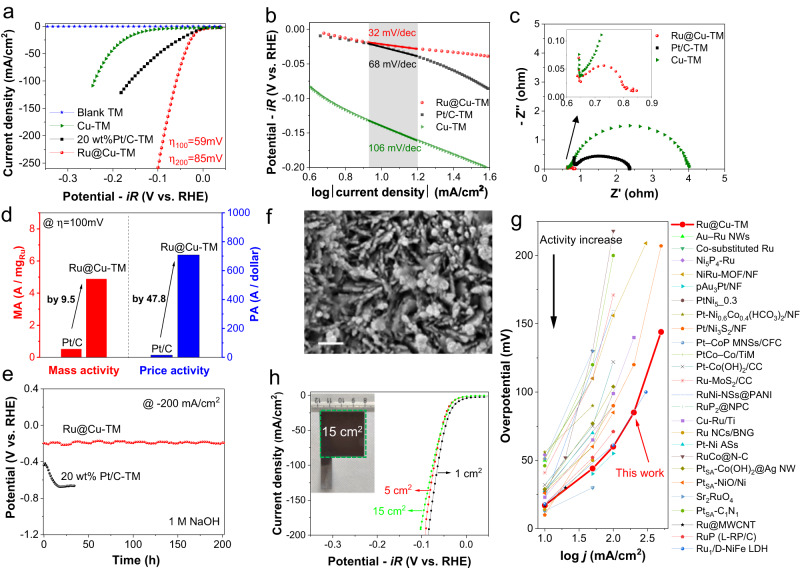
Electrochemical characterization of Ru@Cu-TM cathode at electrode-level. **a** Comparison among the iR-corrected LSV curves measured for blank TM, Ru@Cu-TM and Pt/C-TM benchmark (100 μg_Pt_/cm^2^) deposited onto the same substrate (TM) in 1 M NaOH (electrode geometric area = 1 cm^2^). **b** Tafel slopes, and **c** EIS plots of indicated electrodes. **d** Mass activities and price activities measured for Ru@Cu-TM and Pt/C-TM benchmark at 100 mV HER overpotential. **e** Non iR-corrected chronopotentiometric potential vs. time plots measured for Ru@Cu-TM and Pt/C-TM benchmark; **f** SEM image of Ru@Cu-TM after CP test (200 h at −200 mA/cm^2^). Scale bar: 1 μm. **g** Comparison between the HER activity of our Ru@Cu-TM and PGM-based electrocatalysts for the HER in 1 M KOH/NaOH reported in literature, as listed in Supplementary Table 1. **h** Comparison between the iR-corrected LSV curves measured for 1 cm^2^, 5 cm^2^ and 15 cm^2^ Ru@Cu-TM cathodes. The photograph of a representative 15 cm^2^ Ru@Cu-TM cathode is also reported.

The Ru@Cu-TM electrode displayed a much higher HER activity than that of the Cu-TM and Pt/C-TM benchmark at the same overpotentials, either before (Supplementary Fig. 9a) or after (Fig. 2a) iR correction, achieving a −200 mA/cm^2^ current density at an iR-corrected overpotential of 85 mV (>200 mV for Pt/C). The analysis of the Tafel plot measured on Cu-TM for the HER in 1 M NaOH demonstrated a Tafel slope of 106 mV/dec, while the incorporation of Ru greatly decreases the Tafel slope to 32 mV/dec (Ru@Cu-TM), evidencing that the deposited Ru is the main catalytic site for the HER. The benchmark Pt/C-TM displayed a Tafel slope of merely 68 mV/dec, thus showing slower HER kinetics compared to our Ru@Cu-TM (Fig. 2b). The best activity towards HER on Ru@Cu-TM in 1 M NaOH has been confirmed using the EIS analysis, in which a much smaller resistance of charge transfer has been observed, compared to that of either Cu-TM or Pt/C-TM electrodes (Fig. 2c). Notably, the resistance used for iR compensation, mainly from the electrolyte, was measured as 0.65 Ω for both Cu-TM and Ru@Cu-TM electrodes. In terms of electrode mass activity at 100 mV HER overpotential (calculated by normalizing the geometric current density to mass loading of PGMs, i.e., Ru or Pt), Ru@Cu-TM (mass activity = 4.87 A/mg_Ru_) outperformed the Pt/C benchmark catalyst (mass activity = 0.51 A/mg_Pt_) by almost a factor of 10 (Fig. 2d, see details in the Supplementary Information). Importantly, at 100 mV HER overpotential, the price activity of our catalysts (calculated as electrode current normalized to the cost of PGMs) was found to be as high as 707.8 A/US$_Ru_, around 50 times the one of the Pt/C benchmarks (14.8 A/US$_Pt_) (Fig. 2d).

To better compare the electrodes, their specific activity was calculated by dividing the geometric current density by their corresponding electrochemically active surface area (ECSA) (calculated considering all the material components including non-active species). As shown in Supplementary Fig. 10, at the HER overpotential of 100 mV, Ru@Cu-TM exhibited a specific activity (161 µA/cm^2^_ECSA_) 81% higher than that of Pt/C on TM (89 µA/cm^2^_ECSA_). Meanwhile, the turn over frequency (TOF) of the HER performed with the Ru@Cu-TM cathode at an overpotential of 100 mV (calculated by considering all the Ru atoms as the active sites) is 2.55 s^−1^, which is higher than those reported for PGM-based electrocatalysts working under the same conditions (overpotential of 100 mV), (e.g., 1.27 s^−1^ for Ru_1_/D-NiFe LDH^24^, 0.90 s^−1^ for Sr_2_RuO_4_^25^, 1.41 s^−1^ for Pt/Ni_3_S_2_/NF^26^, 0.0498 s^−1^ for RuNi-NSs@PANI^27^ and 1.23 s^−1^ for PtCo-Co/TiM^28^). In addition, Ru@Cu-TM demonstrated a durable HER activity over >200 h at −200 mA/cm^2^ (Fig. 2e), without exhibiting significant morphological changes (Fig. 2f). Differently, the HER overpotential of the Pt/C-TM increased significantly (>200 mV) over the first 18 h (Fig. 2e). Besides, the Faradaic efficiency for the HER measured on the Ru@Cu-TM cathode operating at −50 mA/cm^2^ was ~100% by gas chromatography (Supplementary Fig. 11).

Overall, these results prove that the designed Ru@Cu-TM can achieve superior (geometric) performances compared to those of the conventional Pt/C benchmark, even when employing half PGM loadings (~53 µg_Ru_/cm^2^ for Ru@Cu-TM vs. 100 µg_Pt_/cm^2^ for Pt/C). Our Ru@Cu-TM outperformed most of PGM-based electrocatalysts for the alkaline HER reported in literature (Fig. 2g, Supplementary Table 1). We validated the Ru@Cu-TM cathode also under conditions mimicking the AEL operational ones, i.e., a highly concentrated alkaline electrolyte (6 M NaOH, equals ~20 wt% NaOH) up to a temperature of 80 ^o^C, exhibiting stable HER-overpotential at a current density of −500 mA/cm^2^ (Supplementary Fig. 12a–c). As expected, the mass transport/diffusion in such industrial conditions is limited compared to that at ambient temperature and low-concentration aqueous electrolytes (Supplementary Fig. 12d), the latter commonly used for the lab-scale evaluation of electrodes for the HER^29^. Lastly, Fig. 2h and Supplementary Fig. 13 evidence that our Ru@Cu-TM cathode can be upscaled to 5 cm^2^, 15 cm^2^ and even 25 cm^2^, while preserving the HER activity. Thus, the developed cathode can meet the requirement for industrial green hydrogen production scenarios.

To understand the role of the substrate in the catalytic activity of our Ru@Cu-TM, cathodes based on mesh substrates other than TM (i.e., Ni mesh—NM, Cu mesh—CM, stainless steel mesh—SSM) were also investigated. Among them, only NM allowed for the vertical growth of CuO nanoplatelets and the subsequent preparation of a Ru@Cu-NM electrode (Supplementary Figs. 14, 15), demonstrating that the CuO nanoplatelets growth is strongly affected by the chemistry (affinity) of the substrate surface. Interestingly, although presenting the same structure of catalyst arrays on the substrate surface (Supplementary Fig. 14d vs. Fig. 1b in the main text), Ru@Cu-NM performed much worse than the Ru@Cu-TM case (Supplementary Fig. 16a), which could be due to the incorporation of Ti species into the latter catalyst. To understand the difference between HER kinetics of Ru@Cu based on TM and NM, EIS measurements were performed at various overpotential conditions to conclude that the incorporated Ti-based species contribute to Volmer step, thus promoting the dissociation of water (see details of Supplementary Figs. 15, 16).

**Fig. 3:**
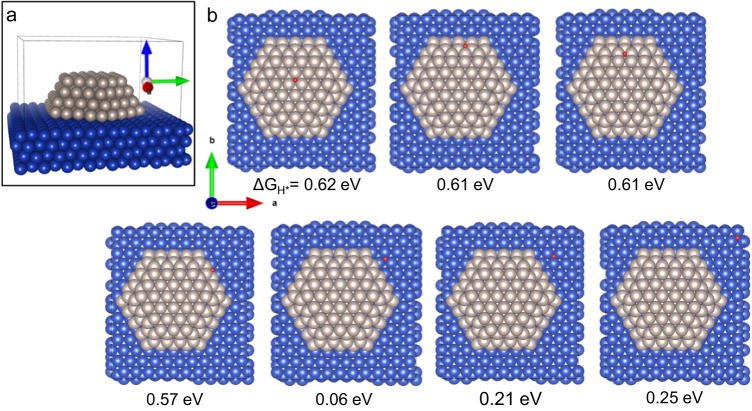
Hydrogen desorption free energy on a Ru nanoparticle atop Cu substrate. The desorption free energy was calculated for representative adsorption positions (**b**). It is defined as ΔG_H*_ = G_CuRu_ + ½ G_H2_—G_CuRu-H_, where G_CuRu-H_ is the free energy of a given system shown in panel **b**, while G_CuRu_ is the free energy of the system shown in **a**. G_H2_ is the free energy of an H_2_ molecule in vacuum (see “Methods” section for the free energy estimation). Cu, Ru, and H atoms are shown as blue, grey, and red spheres, respectively. The adopted unit cell is shown as full gray lines in **a**.

We performed density functional theory (DFT) simulations to understand the role of the various components of our catalyst and to gain insights into the atomistic origin or its functioning. A key step for the HER is the desorption of H_2_, which can occur either through the Heyrovsky or the Tafel steps. The Tafel step is the simple desorption of H_2_ by the combination of two H atoms previously adsorbed on the catalyst surface, while the Heyrovsky step in alkaline environments also involves a proton donation from water and an electron transfer from the cathode^30^. In both cases, it is reasonable to assume that the best catalysts are those that bind H neither too weakly nor too strongly. In fact, it was shown that the best performing HER metal catalysts have a free energy associated to hydrogen desorption (ΔG_H*_) close to zero^31^. Thus, ΔG_H*_ can be used to estimate the HER performance of a catalyst. We adopted DFT to calculate ΔG_H*_ on a realistic model of a Ru nanoparticle on a Cu substrate (Fig. 3a; see also “Methods**”** section). The results (Fig. 3b) show an unexpected trend. While ΔG_H*_ values on Cu and Ru surfaces far from the Cu-Ru interface are similar to that on the pure metal (Supplementary Table 2), this value approaches zero on the Cu regions close to the interface. Thus, the Ru nanoparticle perturbs the Cu substrate and weakens the Cu-H bond, allowing the binding energy to reach an optimal value for HER. In addition, we note that both Ru (Supplementary Table 3) and TiO_2_ spontaneously dissociate water, as also supported by EIS measurements (Supplementary Fig. 16), and previous reports^23^. In alkaline environments, the initial step of HER (Volmer) and possibly also the abovementioned Heyrovsky step involve the dissociation of a water molecule^30^. Thus, it is likely that the presence of species that are active in promoting water dissociation accelerates the overall HER, thereby improving the performances of the catalyst. Overall, our DFT simulations show that the Ru nanoparticles deposited on Cu create optimal regions for the hydrogen desorption, and that the three components of our catalysts (Ru, Cu, and TiO_2_) may act in synergy to promote the various steps of HER, resulting in the observed outstanding catalytic performance.

### Development and optimization of AELs and their cell-level characterization

**Fig. 4:**
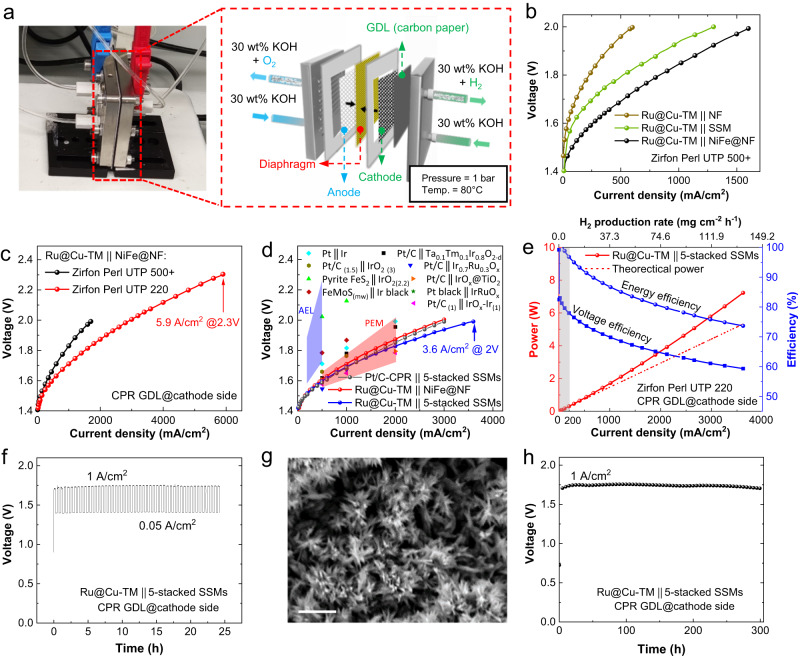
Characterization of AELs at the cell level. **a** Set-up and sketch of the AELs configuration. **b** Polarization curves measured for the following AELs (indicated as anode || cathode): Ru@Cu-TM || NiFe@NF, Ru@Cu-TM | | SSM and Ru@Cu-TM | | NF. No extra GDLs were used for these AELs. **c** Polarization curves measured for Ru@Cu-TM || NiFe@NF AELs using Zirfon diaphragms with different thicknesses: Zirfon Perl UTP 500+ (500 ± 50 μm) and Zirfon Perl UTP 220. A CPR was used as extra GDL at the cathode side. **d** Polarization curves measured using diaphragm of Zirfon Perl UTP 220 with different cathode || anode couples. Pt/C-CPR cathode: 150 μg_Pt_/cm^2^. A CPR GDL was used as extra GDL at the cathode side. Light-blue and red shadings correspond to the operating cell voltages and current densities reported for AELs^11^ and PEM-ELs^73^, respectively. Symbols correspond to PEM-ELs reported recently, as listed in Supplementary Table 6, in which numbers in brackets indicate the catalyst mass loading expressed in mg/cm^2^. **e** Power, H_2_ production rate, energy efficiency (based on the hydrogen HHV) and voltage efficiency as a function of current density on Ru@Cu-TM | | 5-stacked SSMs AEL. The theoretical power for water splitting is based on the thermoneutral voltage at 80 °C, 1 bar. Grey shading indicates an operating region corresponding to poorly practical current densities (<0.20 A/cm^2^). **f** AST of the Ru@Cu-TM | | 5-stacked SSMs AEL using diaphragm of Zirfon Perl UTP 220 for 24 h. **g** SEM image showing the morphology of the surface of the Ru@Cu-TM cathode facing the Zirfon Perl UTP 220 diaphragm after the AST measurement in AEL. Scale bar, 2 μm. **h** Continuous stability test for Ru@Cu-TM | | 5-stacked SSMs AEL using diaphragm of Zirfon Perl UTP 220 at 1 A/cm^2^ for 300 h. All the data shown herein are without iR-correction.

To have a suitable anode benchmark for the evaluation of the AELs, we have developed a series of OER-active electrodes based on NiFe catalysts, by immersing a piece of pre-cleaned NF in a solution of Fe(NO_3_)_3_ and Ni(NO_3_)_2_ (see details in “Methods”), as well as duplicating other efficient OER catalysts taking inspiration from previous reports^32,33^. Since the anode development is not the main objective of this work, we detailed the optimization of the NiFe anode in the Supplementary Information (Supplementary Figs. 17–22, and Supplementary Tables 4, 5). In short, the NiFe@NF anode (prepared at 80 ^o^C for 3 h) stands out for its best performance among the screened candidate anodes (267 mV of overpotential to reach current density of 200 mA/cm^2^) and for its stability (only ~25 mV overpotential increase after 200 h operation at 200 mA/cm^2^). Such NiFe@NF anode was coupled with our developed cathode (Ru@Cu-TM) to assemble single-cell AELs as indicated in Fig. 4a, where 30 wt% aqueous KOH was fed and operated at 80 °C under atmospheric pressure (~1 bar). The whole set-up for AEL test is shown in Supplementary Fig. 24.

Figure 4b reports the polarization curves of our zero-gap AEL using Zirfon Perl UTP 500+ diaphragm and based on Ru@Cu-TM cathode and NiFe@NF anode, in comparison with those based on commercial SSM and NF anodes, here considered prototypical benchmarks for alkaline OER^34,35^. Hereafter, the AELs are named cathode || anode. Based on our electrode characteristics, the configuration of the AEL has been systematically optimized by tuning several parameters (see details in Supplementary Information), including the presence and the type of additional gas diffusion layers (GDLs). Even though both cathode and anode were fabricated on metallic mesh or foam supports that can act as GDLs, the use of carbon paper (CPR) as extra GDL at the cathode side resulted in an optimal trade-off between AEL performance and cost amongst the tested configurations (Supplementary Fig. 25). In addition, the upscaling of the electrode active area from 1 cm^2^ to 5 cm^2^ did not cause any relevant change of the AEL performance (Supplementary Fig. 26).

With the aim of reducing the ohmic resistance of our AELs, thus increasing further their performance, a ~ 220 µm-thick Zirfon Perl UTP 220 diaphragm was then used instead of the thicker Zirfon Perl UTP 500+ (500 ± 50 μm)^13^. Zirfon Perl UTP 220 significantly improved the performance of our previous AELs, achieving current densities of 0.5 A/cm^2^, 1.0 A/cm^2^ and 2.0 A/cm^2^ at cell voltages of 1.60 V, 1.71 V and 1.87 V, respectively (Fig. 4c). Interestingly, the Zirfon Perl UTP 220-based AEL operated at ultrahigh current density of 5.90 A/cm^2^ at cell voltage as low as 2.30 V. Such current densities have been rarely reported in the literature for electrolyzers operating in alkaline media (including AEM-ELs, only two cases^36,37^), indicating the capability to reach state-of-the-art performances of PGM-based PEM-ELs (e.g., 6.0 A/cm^2^ at 2.0 V)^38^.

Starting from our optimized AEL configurations, other anodes were then evaluated to compare their performance with our benchmark NiFe@NF. Our attention was focused on cost-effective SSM as it shows robust RuO_2_-like performance towards OER (Supplementary Fig. 17g) thanks to the presence of surface catalytic hetero-layered Ni-Fe hydroxide/oxide nanostructures originated by dealloying and oxidation of stainless steel during OER operation^39,40^. Acknowledging that the OER reaction still represents the bottleneck of water electrolysis^41^, a multielectrode anode configuration, obtained by stacking multiple SSMs, was tested to balance the catalytic activity of cathode and anode (Supplementary Fig. 27). The performance of the AELs using Ru@Cu-TM as cathode progressively increased with increasing the number of SSMs. Although similar ohmic resistances have been observed amongst the cells (Supplementary Fig. 28), the AELs obtained when employing 5-stacked SSMs as the anode surprisingly outperformed those based on NiFe@NF ones (Fig. 4d) (see explanation in Supplementary Note 3), reaching current densities of 0.5 A/cm^2^, 1.0 A/cm^2^ and 3.6 A/cm^2^ at practical (non iR-corrected) voltages of 1.60 V, 1.69 V and 2.00 V, respectively (Fig. 4d). These values correspond to energy efficiencies (based on the hydrogen HHV and neglecting energy consumption from auxiliary electronics and thermal energy input) of 91.7%, 86.9% and 73.4% (voltage efficiencies of 74%, 70.1% and 59.2%), respectively (Fig. 4e). These performances surpass those of the AEL based on benchmark Pt/C-CPR | | 5-stacked SSMs, although a much higher PGM mass loading was used for the Pt/C cathode (150 μg_Pt_/cm^2^ vs. 53 μg_Ru_/cm^2^ in Ru@Cu-TM). Also, to the best of our knowledge, 3.6 A/cm^2^@2.00 V represents the current state of the art for the AELs, including those based on electrodes with high PGM loadings (see Fig. 4d, Supplementary Table 6, and Note 2). Our AELs, based on Zirfon diaphragm and anode of either NiFe@NF or SSMs, can significantly outperform the single cells based on commercial materials, including AEMs (Supplementary Fig. 29) and electrodes, as shown for an entire commercially available AEL single cell (Supplementary Fig. 30). Even more, as discussed in recent literature for both AELs^42^ and other types of ELs^43^, we remark the importance to validate the catalysts also in the operational electrolyzer environments to bridge the intrinsic properties of materials under realistic operating conditions.

The stabilities of AELs based on Ru@Cu-TM cathode, and anode of NiFe@NF and 5-stacked SSMs were then evaluated and compared. Although the optimized AEL based on NiFe@NF anode demonstrated a performance close to that achieved with 5-stacked SSMs anode, its stability over time was unsatisfactory. As shown in Supplementary Fig. 31a, the Ru@Cu-TM || NiFe@NF AEL exhibited a significant performance decay ( + 220 mV voltage increase) after continuous operation at 0.5 A/cm^2^ for 100 h. The electrochemical instability of NiFe@NF anode was also evident by the presence of brownish residuals in the anode electrolyte feedstock, attributed to Fe species leached from the anode (Supplementary Fig. 31b). The insufficient stability of NiFe@NF anode agrees with previous literature, in which the NiFe-(oxy)hydroxide anodes were found to be unstable under industrially relevant environments (75 °C in 10 M KOH)^41^. Our observations on NiFe@NF in AEL stress the significance of screening electrocatalyts for water electrolyzer at the practical conditions. Contrary to the NiFe@NF case, the Ru@Cu-TM | | 5-stacked SSMs AEL was stable during both intermittent and continuous operations. The intermittent operation of the AELs was evaluated through an in-situ accelerated stress test (AST) and a quasi-continuous operation, aiming to preliminary assess our technology compatibility with renewable source-powered conditions. As similarly reported for other type of electrolyzers (e.g., PEM-ELs)^44^, the AST protocol involved AEL cycling between 0.05 A/cm^2^ and 1.0 A/cm^2^, with each galvanostatic step kept for 15 min and a total test duration of 24 h, while the quasi-continuous test underwent 608 h of 1.0 A/cm^2^ operation interrupted for ca. 50 h every ca. 120 h (experimentally, the operation was stopped during the weekend days, assessing the robustness of our AEL in presence of low-load operation). As shown in Fig. 4f, our AEL operated at nearly stable voltage during consecutive 1.0 A/cm^2^ steps, indicating its reliability under intermittent operating conditions. The Ru@Cu-TM | | 5-stacked SSMs AEL reached the highest performance recorded among the investigated AELs, including the one fabricated using a commercially viable anode, i.e., NiFe_2_O_4_@SSFF^45^ (Supplementary Fig. 32) and could compete with the high-performance PGM-free ELs reported previously (Supplementary Table 4).

A morphology modification of the Ru@Cu-TM surface was detected after the AST operation of the AEL, showing bundle-like Cu nanotubes instead of the original Cu nanoplatelets (Fig. 4g, Supplementary Figs. 33–36). Notably, the optimized AEL could even work stably in quasi-continuous operation for over 600 h (Supplementary Fig. 37). A morphological change similar to that observed at the end of AST test was also observed on the both sides of the Ru@Cu-TM electrode after a quasi-continuous operation test (Supplementary Figs. 38, 39). Such morphological transformation is ascribed to the in-situ dynamical nanostructuring of the Cu nanoplatelets during operation at large current densities in alkaline media (i.e., 1.0 A/cm^2^ and 30 wt% KOH) (Supplementary Fig. 40)^23^. Yet, this morphology change did not affect the catalytic performance of the cathode both at electrode- and cell-levels (Fig. 2e and Fig. 4f, h, respectively), indicating that the chemical environment of Ru-based HER active sites was preserved. The detailed analysis on such transformation is reported in Supplementary Figs. 33–41. Importantly, our Ru@Cu-TM | | 5-stacked SSMs AEL continuously operated at 1.0 A/cm^2^ for 300 h with no significant performance decay (Fig. 4h), confirming the stability of our system under controlled galvanostatic regime with practical current densities. To assess the electrochemical stability of our SSM-based anodes, we evaluated a benchmark Pt/C-CPR | | 5-stacked SSMs AEL, demonstrating a stable performance over 1000 h operation at 1 A/cm^2^ (see details in Supplementary Fig. 42). Thus, the stacked SSMs were confirmed to be a cost-effective, efficient and stable anode for the realization of high-performance and robust AELs, as also indicated in recent studies^46,47^.

Prospectively, the multielectrode concept can be extended to both cathodes and other advanced anodes, eventually including extra GDLs between the stacked electrodes^37^. Lastly, we point out that the cathode technology developed here can be directly implemented into other valuable low-CAPEX water splitting technologies recently proposed in the literature, including electrochemical-thermally activated chemical water splitting^48^ and capillarity-fed AELs^49^, which, so far, operate using alkaline electrolytes. In this context, the designed cathodes can represent advantageous alternatives to traditional cathodes, e.g., Raney-Ni^50^ or other HER electrocatalysts^48^. Indeed, preliminary results showed that our capillarity-fed AELs can reach current densities of 0.5 A/cm^2^ and 0.82 A/cm^2^ at 1.78 V and 2.0 V, respectively (see further details in Supplementary Fig. 43).

### Techno-economic analysis

**Fig. 5:**
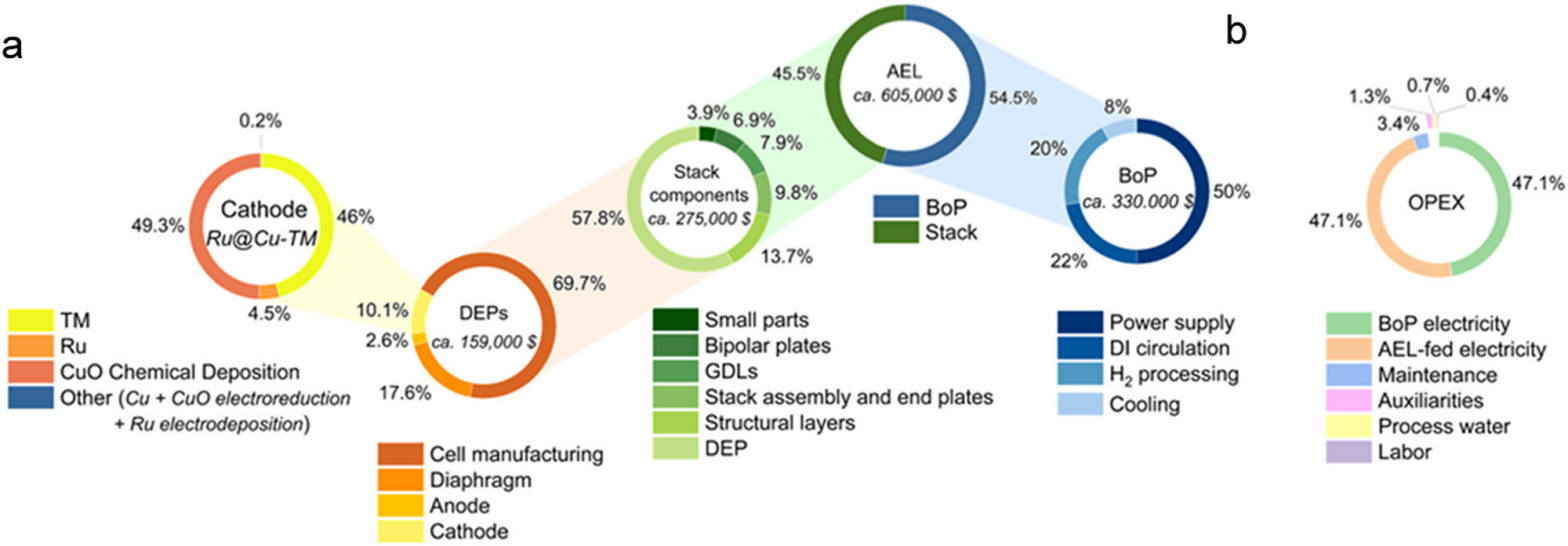
TEA for our AEL technology operating at 1 MW-scale. **a** CAPEX and **b** OPEX of a 1 MW AEL plant (1000 cells) based on the Ru@Cu-TM || Zirfon Perl UTP 220 | | 5-stacked SSMs cell configuration.

A preliminary TEA was performed to roughly assess the cost of H_2_ production on an ideal 1 MW-scale AEL plant based on the technologies reported in this work (see details in Supplementary Information and Supplementary Data 1). By relying on the average data of worldwide currently operating MW-scale AEL plants^51^, the CAPEX and operational expenditure (OPEX) of a 1 MW AEL plant based on Ru@Cu-TM | | 5-stacked SSMs cells (using Zirfon Perl UTP 220 diaphragm) have been calculated (Supplementary Table 7–10). The pie charts in Fig. 5 provide a graphical depiction of the CAPEX and OPEX breakdown of the AEL plant. Noteworthy, the cost associated with a single AEL cell implementing our Ru-based catalysts matches the one calculated from data provided by IRENA and the Korea Institute of Energy Research (KIER) (Supplementary Table 11), which are Raney-type cathodes made of Ni or NiMo alloy-coated perforated stainless steel support^51,52^.

Starting with a bottom-up approach, our TEA (Supplementary Data 1) demonstrates that the low mass loading of Ru results in a moderate impact of the PGM-related cost on the overall cathode CAPEX, accounting only for ~4.5% of the unitary cost of the electrode (Fig. 5a). This result confirms that the nanostructuring of electrocatalysts is an effective method to increase the profitability of the AELs^22,53^. The pie charts in Fig. 5a indicate that the electrode substrate plays a major role in defining the overall cost of the cathodes. Therefore, the replacement of an expensive electrode substrate (e.g., TM) with cheaper ones (e.g., SSM) represents the most straightforward way to decrease the CAPEX of the cathode, which will be discussed hereafter.

Considering the average cost of all the other stack components (e.g., bipolar plates and GDLs), the deployment of 5 stacks of 200 cells, summing up to a total effective power of *~*1.03 MW, would result in a stack-level CAPEX of *~*US$275,000 for the Ru@Cu-TM | | 5-stacked SSMs cell configuration (Fig. 5a). Considering all the AEL’s auxiliaries (i.e., Balance of Plant—BoP—) the estimated total CAPEX for the deployment of a 1 MW scale electrolyzer implementing our cathode is found to be of *~*US$600,000 (Fig. 5a). This CAPEX value is similar to the cost of state-of-the-art large-scale AEL plants, usually ranging from US$500,000/MW to US$1,000,000/MW for 10 + MW systems^51^. The annual OPEX of the AELs ( ~ US$450,000/year) has been calculated considering the cost of the energy (i.e., electricity) fed to the electrolyzer, the water consumption, the labor, the maintenance, and general ancillary costs. As expected for large-scale AEL plants^51^, the main contribution to the annual OPEX of our AELs is given by the electrical energy consumed evenly by electrolyzer component and BoP, accounting for *~*94% of the total OPEX (Fig. 5b). Since the CAPEX contribution to the overall cost of H_2_ production is evenly spread on the plant lifetime through depreciation financial models^54^, the annual OPEX is the parameter that defines the cost of the produced H_2_ and, therefore, the profitability of the AEL. By considering 10-year lifetime of our ideal 1 MW AELs plant, the green hydrogen would be produced at a cost of *~*US$2.26/kg_H2_. A further decrease of the hydrogen production cost to *~*US$2.09/kg_H2_ can be envisaged for an AEL plant lifetime of 30 years^51^. For comparison, a similar TEA has been carried out for a commercial Ni-based single cell AEL (Alkaline Electrolysis Stack, 1 cell, 12 cm^2^ active size—Fuel Cell Corp.)^55^, whose performances are reported in Supplementary Fig. 44. A hydrogen production cost of *~*US$2.61/kg_H2_ is computed for this system (10-year lifetime). Such result demonstrates how cheaper (~0.005 US$/cm^2^ for Ni cathodes versus ~0.011 US$/cm^2^ for Ru@Cu-TM ones) yet less efficient ( ~ 2 V @ 1 A/cm^2^ for Ni cathodes versus ~1.7 V for Ru@Cu-TM ones) electrodes lead to higher hydrogen production costs. This economically justifies the use of Ru on large-scale AELs where OPEX minimization is the key to achieve cost competitiveness. Further comments on this analysis can be found in the dedicated Excel file (Supplementary Data 2). Nevertheless, we are aware that if the activity of PGM-free catalysts will be further enhanced in the future, then the advantages of Ru catalysts would be reconsidered.

Overall, our TEA clearly indicates that our AEL leads to green hydrogen costs already hitting the target set by the European Commission^17^ for the following decade (<US$2.5/kg_H2_ based on average European wind energy productivity), and also approaching the target of U.S. Department of Energy (US$2/kg_H2_ by 2026)^18^.

To check whether the replacement of TM substrate in cathode with other cheaper substrates has a big impact on the H_2_ cost, we have also included additional cathode candidates based on metallic substrates of SSM, NM, and CM. As discussed above, only NM was found to be an adequate support to produce vertical-aligned Cu platelets decorated with Ru as in the TM case, but the performance of the Ru@Cu-NM cathode was significantly inferior to that of Ru@Cu-TM. To solve this issue, we coated these substrates with a thin Ti layer via sputtering (Supplementary Figs. 45, 46), and observed that, except for the CM case (Supplementary Fig. 46), a thin (15 nm- or 100 nm-thick) Ti layer was sufficient to allow for the vertical growth of CuO nanoplatelets, as in the case of the TM substrate. The electrodes based on Ti-coated NM and Ti-coated SSM exhibited a HER activity comparable with that of Ru@Cu-TM in both three-electrode configuration (Supplementary Fig. 47) and AEL tests (Supplementary Fig. 48). These results prove that both Ti-coated NM and Ti-coated SSM are suitable low-cost substitutes of TM for the proposed cathode technology, which will then be taken into consideration. We therefore calculated the H_2_ production costs of plants based on either Ru@Cu-Ti@NM || NiFe@NM or Ru@Cu-Ti@SSM | | 5-stacked SSMs AELs (see Supplementary Figs. 49, 50). Cathodes based on TM substrate have an estimated manufacturing cost of US$0.022/cm^2^, while replacement of TM with NM or SSM results in a ~25% (US$0.017/cm^2^) and *~*50% (US$0.013/cm^2^) reduction of cathodes unitary cost, respectively (Supplementary Data 1). Since other costs related to diaphragm/electrode package (DEPs) (i.e., anode, diaphragm, and cell manufacture) remain almost constant in the various AEL configurations, the implementation of less expensive cathodes result in a lower CAPEX for DEPs manufacture (e.g., from *~*US$159,000 to *~*US$150,000 for 1,000 cells passing from TM to SSM as the support, Supplementary Fig. 50a). Noteworthy, the cost of labour and coating process of Ti is not included to simplify the model.

The predicted H_2_ production costs for different cathode || anode combinations are summarized in Table 1. Interestingly, the total production cost is mainly affected by the performance of the AEL rather than by its CAPEX (see Supplementary Table 12), while the replacement of TM with Ti-coated SSM, decreasing the H_2_ costs by only US$0.005/kg_H2_, is not a valid solution, considering also the possible risk of Ti detachment from the surface of SSM in industrial practical scenarios and a more complex production procedure (i.e., additional step of Ti sputtering onto SSM). As a conclusion, our TEA (Supplementary Data 1) reveals that our best AEL, namely Ru@Cu-TM | | 5-stacked SSMs, represents the most effective choice for cheap H_2_ production.

**Table 1:** Predicted H_2_ production cost (US$/$${{{{{{\rm{kg}}}}}}}_{{H}_{2}}$$) on an ideal 1 MW AEL plant based on the catalysts reported in this work

Anode	Case 1: NiFe@NF	Case 2: 5-stacked SSMs
Cathode		
Case 1: Ru@Cu-TM	2.259 (2.090^a^)	2.260 (2.091)
Case 2: Ru@Cu-Ti@NM	2.411^b^ (2.233^b^)	–
Case 3: Ru@Cu-Ti@SSM	–	2.255 (2.088)

^a^Number in bracket: considering the lifetime of the AEL plant to be 30 years instead of the default 10 years.

^b^Diaphragm: Zirfon Perl UTP 500 + , differently from all other configurations that used the thinner Zirfon Perl UTP 220.

Finally, as Ru belongs to the PGMs, its scarce availability^56^ has also to be considered when assessing the large-scale deployment of our AEL technology. Our preliminary calculations (see details in “Methods”) return a Ru annual usage of ca. 21 metric tons (ca. 70% of the annual Ru production^51^). Such a mass is relatively low when compared with the Pt one that would be required to achieve the same global electrolysis power (ca. 535,000 metric tons/year, in the face of a ca. 200,000 metric tons annual production^51^). The main reason behind this disparity must be sought in the PGM mass loading per deployed power: indeed, state-of-the-art Pt-based PEM-ELs require 1 g_Pt_/kW_PEMEL_^51,57^, while our Ru-based electrodes have demonstrated (at lab scale) a mass to power ratio as low as ca. 0.04 g_Ru_/kW_AEL_ (see calculation details in Supplementary Data 1), stemming from the low Ru loading (ca. 53 μg/cm^2^) of our cathode technology. Having above predicted cost on H_2_ production and feasibility of implementing our Ru@Cu-TM cathode into a 1 MW plant, we recognize that calculations based on our lab-scale data of the single AEL cell may not represents accurately the actual plant-level data. However, this work supports the evidence that a small amount of PGMs (Ru in our case) allows for a large reduction in the OPEX. Hence, AELs can reach the same level of performance as PEM-ELs in terms of efficiency, while preserving the merits of AEL technology (especially in terms of robustness).

In conclusion, we design a cost-effective and robust cathode for the HER processes, achieving state-of-the-art performances. The HER activity of the Ru@Cu-TM cathode surpasses that of the Pt/C benchmark, achieving a current density of −0.2 A/cm^2^ at an overpotential of 0.085 V (>0.20 V for Pt/C). By means of DFT simulations supported by experimental analyses, we ascribe the high performance to multiple effects: 1) the deposited Ru nanoparticle perturbs the Cu substrate and weakens the Cu-H bond to favour hydrogen adsorption-desorption; 2) Ru and TiO_2_ facilitate water dissociation, thus leading to high performance of Ru@Cu-TM towards alkaline HER. Our cathode, upscaled up to 25 cm^2^, contains only 53 µg/cm^2^ Ru, which is 10 times lower than that used for Pt in the Pt/C cathode of PEM-ELs (e.g., 600 µg/cm^2^)^58^, demonstrating its cost-effectiveness. Besides, we discovered that 5-stacked SSMs could be a simple and cost-effective anode to replace the well-performant NiFe anode screened by us for designing AELs, with unprecedented performance and long-term durability. Our observations herein stress the significance of screening electrocatalyts for water electrolyzer at the industrial working conditions. Indeed, we demonstrate that the Ru@Cu-TM | | 5-stacked SSMs AEL can reach current densities as high as 1.0 and 3.6 A/cm^2^ at voltage of 1.69 and 2.0 V, respectively, corresponding to energy efficiencies (based on hydrogen HHV) of 86.9% and 73.4% (voltage efficiencies of 70.1% and 59.2%) with a satisfactory durability. The latter was demonstrated in a continuous mode at 1.0 A/cm^2^ and 80 °C for 300 h, as well as in intermittent-modes. These experimental data set allowed us to carry out a TEA, assessing the cost for the green hydrogen production in a 1 MW plant. By estimating its total cost (including CAPEX and OPEX), the predicted total hydrogen production cost was found to be US$2.09/kg_H2_ for 30-year plant lifetime (US$2.26/kg_H2_ for 10-year lifetime). We therefore prove that low amount of Ru in the cathode and the use of TM substrate have negligible impacts on the green hydrogen production cost and that Ru availability issues are relieved by the ultra-low Ru mass loading-to-deployed AEL power ratio (ca. 0.04 g_Ru_/kW_AEL_) of the cathode, the proposed technology is already competitive with grey hydrogen production methods, while almost meeting the worldwide targets for the cost of green hydrogen set by the US and EU (US$2–2.5/kg_H2_). Hence, although PGMs are typically not considered for AELs, we support that incorporation of a small amount of Ru could be the game-changer in terms of cutting down the OPEX, without significantly impacting on the overall CAPEX of traditional AELs.

## Methods

### Chemicals

Copper (II) chloride dehydrate (99.999%), sodium hydroxide (NaOH) (98%), ammonia solution (25%), potassium hexachlororuthenate(IV) (K_2_RuCl_6_) (99.95%), nickel (II) nitrate hexahydrate [Ni(NO_3_)_2_ ∙ 6H_2_O] (98.5%), and NF (1.6 mm thickness) were purchased from Sigma-Aldrich. Iron (III) nitrate nonahydrate [Fe(NO_3_)_3_ ∙ 9H_2_O] (99%) was purchased from EMSURE-Merck. Ti mesh (60 mesh, 0.2 mm thickness), NM (60 mesh, 0.2 mm thickness), SSM (80 mesh, 0.18 mm thickness, Type 316) and CM (60 mesh, 0.19 mm thickness) were purchased from Fisher Scientific. All the chemicals were used as received. Mesh substrates were cleaned with isopropanol/ethanol (1:1, v/v) and distilled water, and dried using a N_2_-gun stream. Pt@TFF and (PTFE-untreated) CPRs (AvCarb MGL280) used as GDLs were purchased from FuelCell Store. Zirfon Perl UTP 500+ and Zirfon Perl UTP 220 used as diaphragms were purchased from Agfa. Sustainion X37-50 grade 60 AEM was purchased from Dioxide Materials. Fumasep FAA-3-PK AEM was purchased from FuelCell Store.

### In-situ deposition of Ru nanoparticles onto Cu nanoplatelets grown on TM (Ru@Cu-TM)

As sketched in Fig. 1a, the preparation of Ru@Cu nanoplatelets onto the substrate of Ti mesh (denoted as Ru@Cu-TM) included the following two steps:

#### Synthesis of CuO nanoplatelets

CuO nanoplatelets were grown on TM by a chemical bath deposition strategy, modifying protocols reported in our previous work^23^. Typically, 5 mmol copper (II) chloride dihydrate was added in a beaker containing 100 mL Milli-Q water, and 5 mL ammonia solution (25%) was added inside dropwise until observing a blue color solution. A TM (2.5 cm × 5 cm) was vertically placed and then heated up to 90 °C for 2 h using a water bath. After reaction completion, the TM with CuO nanoplatelets vertically grown onside (CuO-TM) was washed thoroughly with Milli-Q water and dried with a N_2_ stream. A mild sonication of 5 s was used to remove excess of CuO microflowers consisting of nanoplatelet aggregates formed at the edges of TM substrate.

#### Electroreduction of CuO nanoplatelets and electrodeposition of Ru nanoparticles

To prepare a 1 cm^2^ electrode of Ru@Cu-TM, the initial prepared CuO-TM electrode (Supplementary Fig. 1a) was cut into 1.5 cm×1 cm and used as a working electrode with 1 cm^2^ area been immersed in a 25 mL of 1 M NaOH solution. Afterwards, a negative current of −5 mA/cm^2^ (CP protocol) was applied using Ivium-n-Stat potentiostat (Supplementary Fig. 1b) to reduce the CuO layer to metallic Cu (Supplementary Fig. 1c), until the electrode potential became stable. This electroreduction process transformed CuO-TM into Cu nanoplatelets on the TM (Cu-TM). Then, Ru nanoparticles were electrodeposited onto the surface of the Cu-TM by adding 500 µL K_2_RuCl_6_ solution (1 mg/mL, in water) into the electrolyte solution while a potential of −0.2 V vs. RHE was applied to the working electrode (CA protocol). During cathode optimization, other potentials, and even CP protocols, were evaluated, as discussed in the main text. As shown in Supplementary Fig. 1d, the current increased immediately upon the Ru precursor addition, indicating the beginning of the Ru deposition, as well as the HER. After *~*3 h, the current became stable, indicating the completion of Ru@Cu-TM cathode (Supplementary Fig. 1e). The electrochemical cell was placed onto a hot-plate with a temperature set at 30 ^o^C to minimize the temperature fluctuation during Ru@Cu-TM electrosynthesis processes (in this way, the temperature of the electrolyte was maintained at *~*27 ^o^C, as measured by a thermometer).

#### Upscaling of cathode

To upscale the area of Ru@Cu-TM electrode to 5, 15 and 25 cm^2^, the synthesis protocol was similar to the 1 cm^2^ case, adjusting the applied potential by taking into account the different iR drop derived from the change of series resistance measured at high-frequency (10 kHz) through EIS measurements (so that the same iR-corrected potential was applied).

### In-situ deposition of Ru nanoparticles onto Cu nanoplatelets grown on other mesh substrates (NM, SSM)

The deposition of Ru nanoparticles onto Cu nanoplatelets grown on NM was carried out following the protocols described above, except that TM was replaced by NM and Ru@Cu-NM was therefore obtained. When SSM was instead used as the substrate, the protocols described above did not work. Therefore, before depositing CuO nanoplatelets, a 15 nm layer of Ti layer was sputtered onto the surface of SSM (15Ti@SSM) using a sputter coater (Q150T ES PLUS, FTM model, tooling factor: 3.4). The rest of the procedure above-described led to the electroreduction of CuO nanoplatelets (Cu-15Ti@SSM), followed by the electrodeposition of Ru nanoparticles, delivering the electrode named Ru@Cu-15Ti@SSM. By using NM instead of SSM as the starting substrate, the sample named Ru@Cu-Ti@NM was also prepared. The thickness of the sputtered Ti layer was varied among the investigated cathodes, leading to a Ti coating denoted as xTi, where x is equal to the thickness of the coating expressed in nm (i.e., 100 nm-thick Ti, named 100Ti). To obtain a complete coverage, we selected 15 nm as the minimum thickness for Ti coating. Preliminary tests indicated that, regardless of Ti deposition, the CuO nanoplatelets cannot be grown vertically on the surface of CM.

### Preparation of a control 20 wt% Pt/C-TM cathode

2 mg of commercial 20 wt% Pt/C (platinum on graphitized carbon, Sigma-Aldrich) were dispersed into water/isopropanol (0.2/0.19 ml) and then 10 μl of Nafion (10 wt% Nafion 117 containing solution, Sigma-Aldrich) was added into the dispersion. The mixture was sonicated for 30 min to obtain a homogeneous ink, and then drop-casted onto the TM electrode with a mass loading of 0.5 mg/cm^2^, corresponding to a Pt loading of 100 μg/cm^2^. The prepared electrode was dried in air before measurements.

### In-situ fabrication of NiFe@NF as the anode

The method used to produce NiFe@NF was inspired by previous reports^32,33^, whose protocols were, however, modified to maximize the anode OER activity. In practice, a piece of NF was cleaned using ethanol/acetone (1/1, v/v) and HCl solution (1 M), respectively. The cleaned NF was immersed in a solution containing 50 mM Fe(NO_3_)_3_ and 50 mM Ni(NO_3_)_2_ at 80 ^o^C for 3 h. The resulting NiFe@NF electrode (also denoted as Ni50Fe50_3h@80@NF to specify synthesis parameters) was washed with water and dried using a N_2_ stream.

#### Upscaling of anode

The size of the electrode was easily upscaled according to the size of starting NF, without any protocol modification.

### Preparation of a control RuO_2_/NF anode

The preparation of the RuO_2_/NF anode is similar to the one of the 20 wt% Pt/C-TM cathode, except that commercial RuO_2_ was used instead Pt/C, and the mass loading of RuO_2_ onto NF electrode was 1.0 mg/cm^2^. The prepared electrode was dried in air before the measurements.

### Preparation of a control NiFe/NF anode

Nickel-iron hydroxide (NiFe) nanosheets were electrodeposited onto surface of NF by replicating Zhao et al.’s method^59^. Typically, a piece of pre-cleaned NF as the working electrode, a double-junction Ag/AgCl (saturated KCl) as the reference electrode, and a winded Pt wire as the counter electrode were mounted into an electrolyte solution containing 3 mM Ni(NO_3_)_2_·6H_2_O and 3 mM Fe(NO_3_)_3_·9H_2_O. The electrodeposition of NiFe was carried out at a constant potential of −1.0 V vs. Ag/AgCl for 300 s at *~*5 ^o^C. The as-produced NiFe/NF electrode was taken out from the electrolyte and washed with water. Afterward, the electrode was dried using a N_2_ stream.

### Characterization of electrode materials

XRD measurements were performed on a PANalytical Empyrean using Cu Kα radiation. XPS measurements were carried out on a Kratos Axis UltraDLD spectrometer using a monochromatic Al Kα source, operated at 20 mA and 15 kV. High resolution analyses were carried out at pass energy of 10 eV. Spectra were calibrated based on the mainline of carbon 1 s spectrum set to 284.8 eV. SEM images were acquired on an FEI NanoLab 600 dual beam system with an acceleration voltage of 5-10 kV, while EDS was performed at the voltage of 20 kV. Overview TEM images were acquired using a JEOL JEM-1011 microscope operated at 100 kV. The TEM samples were prepared by sonicating the catalyst electrode in ethanol to peel off the catalyst from the TM substrate and obtain a catalyst dispersion. Such dispersion was then drop-casted onto ultrathin carbon-coated Cu grids. HAADF-STEM and HRTEM imaging was carried out on a JEOL JEM-2200FS TEM (Schottky emitter), operated at 200 kV, equipped with a CEOS corrector for the objective lens and an in-column image filter (Ω-type). To avoid any beam damage to the sample particles, they were exposed to a comparably low dose rate ( ~ 30 electrons/(Å^2^ s)) and HRTEM images were acquired using a direct electron detector (K2 Summit, Gatan), in super-resolution mode. Each image shown here is a portion of the (260 nm)^2^ frame obtained by summing aligned 40 frames obtained at a short exposure time (0.3 s), with a total acquisition time of 12 s. STEM-EDS data were acquired by a Bruker XFlash 5060 EDS system installed on the same microscope and quantification was carried out by the Cliff-Lorimer method, using the K series of Cu, Ru, Ti, K and O. The HRTEM samples were prepared by depositing catalyst dispersion onto a holey carbon-film coated Au grid. For STEM-EDS analyses, an analytical holder was used. ICP-OES was carried out on an iCAP 6500 Thermo spectrometer. Depending on the requirements, the samples were prepared by dissolving either the whole catalyst electrode (of known size) or the collected catalyst powder from the substrate surface in 2.5 mL aqua regia (HCl/HNO_3_ 3:1, v/v) overnight for digestion. The resulting solution was then diluted to 25 ml with Milli-Q water, and ~10 mL solution was collected after filtering using a 0.45 μm Nylon filter. The ICP measurements were affected by a systematic error of ~5%.

### Electrochemical tests of the electrodes

The electrochemical characterization of the electrodes was carried out using an Ivium multichannel potentiostat and a three-electrode cell configuration. Note: Although for the evaluation of HER catalysts the use of carbon rod is recommended instead of Pt counter electrode (due to the possible deposition of dissolved Pt onto the working electrode^60^), the carbon oxidation leads to the release of carbon ashes during tests at high current density^61^. Besides, several investigations have been carried out to exclude the influence of possible Pt deposition onto the electrode surface (see details in Supplementary Fig. 23). A 1 M NaOH solution was used as the electrolyte. Beyond the produced cathodes and anodes (see synthesis part), as-received SSM and NM were also evaluated to benchmark the electrocatalytic performance of our anodes, together with a commercially available anode supplied by Dioxide Materials. The latter consists of NiFe_2_O_4_ particles with a Nafion binder on a 316 L sintered stainless steel fibre felt (anode named NiFe_2_O_4_@SSFF)^45^. Linear sweep voltammetry curves were acquired at a scan rate of 2 mV/s and were iR-corrected, while CP and CA plots were displayed without iR-correction. For anode analysis, a high scan rate of 50 mV/s was used only for the CV activation (20 cycles), while a low scan rate of 2 mV/s was used to investigate the electrode catalytic activity for the OER. To measure the electrode series resistance (R), EIS measurements were performed on the electrodes at −0.2 V vs. RHE for cathode and at 1.52 V vs. RHE for anode, using a frequency range of 0.1 Hz-100 kHz. EIS spectra of Ru@Cu-TM and Ru@Cu-NM were acquired at various potentials (25, 0, −25, −50, −100 mV vs. RHE) in a frequency range of 0.01Hz-50 KHz. The durability of the working electrode was assessed through CP measurements by fixing the current density at 200 mA/cm^2^. The recorded potentials were converted to the RHE scale according to the Nernst equation, i.e.:

1 $${{{{{{\rm{E}}}}}}}_{{{{{{\rm{RHE}}}}}}}={{{{{{\rm{E}}}}}}}_{{{{{{\rm{obs}}}}}}}+{{{{{{\rm{E}}}}}}}_{{{{{{\rm{Ag}}}}}}/{{{{{\rm{AgCl}}}}}}}^{{{{{{\rm{o}}}}}}}+0.0591\times {{{{{\rm{pH}}}}}}={{{{{{\rm{E}}}}}}}_{{{{{{\rm{obs}}}}}}}+1.02{{{{{\rm{V}}}}}}$$

The value of (E^o^_Ag/AgCl_ + 0.0591×pH) was experimentally measured through the experimental calibration process described in previous literature^19^. The experimentally measured value of ~1.02 V was comparable to the theoretical value obtained from Nernst equation calculation: 1.024 V. The Tafel slope was used as a metric to evaluate the HER kinetics of our electrodes. Such parameter can be estimated from the linear portion of the Tafel plot (overpotential vs. log( | current density | ) curve) according to the Tafel equation:

2 $${{{{{\rm{Overpotential}}}}}}={{{{{\rm{b}}}}}}\times {{{{{\rm{|}}}}}}{{\log }}({{{{{\rm{current\; density}}}}}}){{{{{\rm{|}}}}}}+{{{{{\rm{A}}}}}}$$

in which the overpotential is referenced to the 0 V vs. RHE potential, b is the Tafel slope and A is a constant. For an insufficient adsorbed hydrogen (H*) surface coverage, the Volmer reaction is the rate‐limiting step of the HER, and a theoretical Tafel slope of 120 mV/dec is expected. Conversely, in the limit of high H* surface coverage, the Tafel slope decreases towards the theoretical values of 40 or 30 mV/dec, expressing the HER kinetics of the Heyrovsky reaction or Tafel reaction^62^. See these reaction steps below:

3 $${{{{{\rm{Volmer}}}}}}:\,{{{{{{\rm{H}}}}}}}_{2}{{{{{\rm{O}}}}}}+{{{{{{\rm{e}}}}}}}^{-}\rightleftarrows {{{{{{\rm{H}}}}}}}^{*}+{{{{{{\rm{OH}}}}}}}^{-}$$

4 $${{{{{\rm{Heyrovsky}}}}}}:{{{{{{\rm{H}}}}}}}_{2}{{{{{\rm{O}}}}}}+{{{{{{\rm{H}}}}}}}^{*}+{{{{{{\rm{e}}}}}}}^{-}\rightleftarrows {{{{{{\rm{H}}}}}}}_{2}+{{{{{{\rm{OH}}}}}}}^{-}$$

5 $${{{{{\rm{Tafel}}}}}}:{2{{{{{\rm{H}}}}}}}^{*}\rightleftarrows {{{{{{\rm{H}}}}}}}_{2}$$

To evaluate the performance of electrodes under simulated industrial conditions, a 6 M NaOH solution was used as the electrolyte in a cell made of polypropylene put on a hot-plate with feedback-detector insert into electrolyte through a tube. The temperature was varied from 30 ^o^C to 80 ^o^C. A 6 M KOH-filled Hg/HgO electrode with a PTFE-body was used as the reference electrode, which was calibrated at each temperature before measurements.

***The TOF*** of Ru@Cu-TM cathode was calculated according to:

6 $${{{{{\rm{TOF}}}}}}=\frac{{{{{{\rm{number}}}}}}\; {{{{{\rm{of}}}}}}\; {{{{{\rm{hydrogen}}}}}}\; {{{{{\rm{molecules}}}}}}}{{{{{{\rm{number}}}}}}\; {{{{{\rm{of}}}}}}\; {{{{{\rm{active}}}}}}\; {{{{{\rm{sites}}}}}}}$$

in which

7 $${{{{{\rm{Number}}}}}}\; {{{{{\rm{of}}}}}}\; {{{{{\rm{hydrogen}}}}}}\; {{{{{\rm{molecules}}}}}}=\left(\frac{{{{{{\rm{j}}}}}}}{1000}\times {{{{{{\rm{N}}}}}}}_{{{{{{\rm{A}}}}}}}\right)/\left({{{{{\rm{F}}}}}}\times {{{{{\rm{n}}}}}}\right)=3.12\times {10}^{15}{{{{{\rm{|j|}}}}}}\frac{{{{{{{\rm{H}}}}}}}_{2}/{{{{{\rm{s}}}}}}}{{{{{{{\rm{cm}}}}}}}^{2}}{{{{{\rm{per}}}}}}\frac{{{{{{\rm{mA}}}}}}}{{{{{{{\rm{cm}}}}}}}^{2}}$$

being j the current density expressed in mA/cm^2^, N_A_ the Avogadro constant (6.022 × 10^23^/mol), F the Faraday constant (96485 C/mol) and n the number of electrons transferred to generate one molecule of the H_2_ (i.e., *n* = 2)

8 $$	{{{{{\rm{Number}}}}}}\; {{{{{\rm{of}}}}}}\; {{{{{\rm{Ru}}}}}}\; {{{{{\rm{sites}}}}}}\left({{{{{\rm{assuming}}}}}}\; {{{{{\rm{all}}}}}}\; {{{{{\rm{the}}}}}}\; {{{{{\rm{electrodeposited}}}}}}\; {{{{{\rm{Ru}}}}}}\; {{{{{\rm{to}}}}}}\; {{{{{\rm{be}}}}}}\; {{{{{\rm{active}}}}}}\right) \\ 	\;=\left(\frac{{{{{{\rm{mass}}}}}}\; {{{{{\rm{loading}}}}}}\; {{{{{\rm{of}}}}}}\; {{{{{\rm{Ru}}}}}}\; {{{{{\rm{determined}}}}}}\; {{{{{\rm{by}}}}}}\; {{{{{\rm{ICP}}}}}}\; {{{{{\rm{per}}}}}}\; {{{{{\rm{geometric}}}}}}\; {{{{{\rm{area}}}}}}}{{{{{{\rm{Ru}}}}}}\; {{{{{\rm{Mw}}}}}}}\right)\times {{{{{{\rm{N}}}}}}}_{{{{{{\rm{A}}}}}}} \\ 	\;=\frac{53\times {10}^{-6}\frac{{{{{{\rm{g}}}}}}}{{{{{{{\rm{cm}}}}}}}^{2}}}{101.1\frac{{{{{{\rm{g}}}}}}}{{{{{{\rm{mol}}}}}}}}\left(\frac{6.022\times {10}^{23}}{1{{{{{\rm{mol}}}}}}}\right)=3.157\times {10}^{17}\left({{{{{\rm{Ru}}}}}}\; {{{{{\rm{sites}}}}}}\right){{{{{\rm{per}}}}}}{{{{{{\rm{cm}}}}}}}^{2}$$

Therefore,

9 $${{{{{\rm{TOF}}}}}}=\frac{3.12\times {10}^{15}}{3.157\times {10}^{17}}{{{{{\rm{|j|}}}}}}=\frac{0.00988{{{{{\rm{|j|}}}}}}}{{{{{{\rm{s}}}}}}}{{{{{\rm{per}}}}}}\frac{{{{{{\rm{mA}}}}}}}{{{{{{{\rm{cm}}}}}}}^{2}}$$

***The ECSA of the cathodes*** was measured by performing CV measurements in a non-Faradaic potential region (from −0.90 V to −0.96 V, vs. Ag/AgCl) at the following potential scan rates: 10, 20, 30, 40, 50, 60 and 80 mV/s. The C_dl_ was determined by plotting the Δ*j*/2 versus of the scan rate (ν), where Δj indicates the current density between the cathodic and anodic sweeps at a certain potential within the scan window (−0.93 V vs. Ag/AgCl). The slope of the linear regression fit is equal to the C_dl_ value, which is proportional to the ECSA of the electrocatalyst. The ECSA is therefore calculated as:

10 $${{{{{\rm{ECSA}}}}}}={{{{{{\rm{C}}}}}}}_{{{{{{\rm{dl}}}}}}}/{{{{{{\rm{C}}}}}}}_{{{{{{\rm{s}}}}}}}$$

in which C_s_ is the specific electrochemical double-layer capacitance of an atomically smooth surface, as assumed equal to 0.04 mF/cm^2^, as reported previously^63^. Note: As discussed in this work, the C_s_ values are different for various materials and the value is also different for the same material in alkaline and acidic conditions. It is therefore difficult to accurately determine the specific capacitance of a specific catalyst that contains various species. Since most of those reported materials demonstrated a specific capacitance between 0.022 and 0.04 mF/cm^2^, we assumed 0.04 mF/cm^2^ as the specific capacitance for our samples.

***The Faradaic efficiency*** for the HER of the cathode was determined by measuring the evolved H_2_ through a gas chromatograph (SRI instruments) equipped with a HayeSep D porous polymer column, thermal conductivity detector, and flame ionization detector. Ultra-pure N_2_ gas (99.999%) was bubbled inside the cathode side of a well-sealed H-cell, where cathodes operated at constant current density of −50 mA/cm^2^ (CP mode), allowing the Faradaic efficiency to be continuously monitored.

The Faradaic efficiency for the HER of the cathode was calculated as:

11 $${{{{{\rm{Faradaic\; efficiency}}}}}}=\frac{{{{{{\rm{n}}}}}}\times {{{{{\rm{F}}}}}}\times {{{{{\rm{C}}}}}}\times {{{{{\rm{G}}}}}}\times {{{{{\rm{P}}}}}}}{{{{{{\rm{R}}}}}}\times {{{{{\rm{T}}}}}}\times {{{{{\rm{i}}}}}}}$$

where n is the number of electrons transferred to generate one molecule of the H_2_ (*n* = 2), F is the Faraday constant (96485 C/mol), C is the measured concentration of the product by gas chromatography (in ppm), G is the gas flow rate (mL/min), P is the working pressure (1.01×10^5 ^Pa), R is the universal gas constant (8.314 J∙mol^−1^ K^−1^), T is the room temperature (293.15 K), and i is the working current applied to the electrode.

### First-principles simulations

#### General simulation settings

All simulations were performed through the VASP code^64^, adopting the Perdew-Burke-Ernzerhof (PBE) exchange correlation functional^65^ within the framework of the projector augmented-wave (PAW) method^66^. When not otherwise stated, the plane wave cutoff was set to 360 eV and the reciprocal space was sampled through a single k point (“Gamma-only”) since the adopted unit cells were considerably large (28 × 31 × 24 Å). The structures of the simulated systems are shown in Fig. 3; here we highlight a few details. (i) The Cu slab was built from the bulk Cu whose geometry was fully optimized with DFT. (ii) The periodic images along the direction perpendicular to the slab are separated by at least 9 Å of vacuum. (iii) During the slab geometry optimizations, the unit cell parameters and the positions of the Cu atoms in the bottom layer were kept frozen, to simulate the presence of the bulk underlying the surface in the real system. All other atoms were allowed to reach their minimum-energy position. (iv) The structure of the Ru nanoparticle was taken from ref. ^67^ as the most stable structure of that size, cut in half, and placed on the Cu surface. The top layer atoms were removed to avoid making the computational load unmanageable (the systems of Fig. 3 already contain 790 atoms), since these atoms were not relevant to study the adsorption energy at the Cu-Ru interface.

#### Free energy estimation

We obtained a reliable estimation of experimental free energy of adsorption ΔG^H^_ads_ (=-ΔG_H*_) by approximating it with the DFT-calculated adsorption energy, ΔE^H^_ads(PBE)_ and by exploiting error compensation. Indeed, ΔE^H^_ads(PBE)_ deviates from ΔG^H^_ads_ because of two types of error: neglect of dynamical effects (ε_dyn_), i.e., changes in vibrational and entropic contributions upon adsorption, and the intrinsic overbinding error of the adopted DFT functional (ε_PBE_). The vibrational contribution was shown to be 0.04 eV^31^. The entropic contribution can be calculated from the data measured on adsorbed^68^ and gas phase^69^ hydrogen, and it amounts to 0.11 eV/atom (see details in Supplementary Information, “Experimental data on hydrogen adsorption free energy”). Thus, ε_dyn_ = 0.04 + 0.11 = + 0.15 eV. ε_PBE_ is −0.06 eV and −0.14 eV for Cu^68^ and Ru^70^, respectively (Supplementary Information). While ε_PBE_ and ε_dyn_ do not exactly sum up to zero, their difference is fully comparable to the experimental uncertainty (e.g., >0.10 eV in ref. ^68^), thus making ΔE^H^_ads(PBE)_≈ΔG^H^_ads_. Finally, the DFT adsorption energy was calculated as ΔE^H^_ads(PBE)_ = -ΔE_H*_ = E_CuRu-H_—E_CuRu_ - ½ E_H2_ where the three terms are the DFT energy of, respectively, the CuRu system with adsorbed hydrogen shown in Fig. 3b, the CuRu system of Fig. 3a, and the gas phase H_2_.

### Electrochemical characterization of the AELs

The AEL were produced using a zero-gap single electrolysis cell (Dioxide Materials), including corrosion resistant Ni-based anode and cathode flow field (bipolar) plates, o-ring seals, and Teflon gasketing. When specified, CPR and Pt@TFF were used as extra GDLs (in principle, our electrodes are gas diffusion electrodes that do not strictly require the use of additional GDLs). Depending on the AEL, the cathode was one of those described in afore-mentioned synthesis part, while the anode was one of the following: NF, SSM, 3 or 5-stacked SSMs (multielectrode-type anode), NiFe@NF and NiFe_2_O_4_@SSFF. Zirfon Perl UTP 500+ or Zirfon Perl UTP 220 were used as diaphragms with different thicknesses (500 ± 50 μm and <250 µm, respectively). The cell components were compressed during installation to realize a (quasi) zero-gap assembly. For thicker diaphragm or multielectrode-type anodes, a compressible EPDM spacer was used to avoid excessive compression and avoiding unproper sealing leading to electrolyte leakage. Beside AELs, two AEM-ELs based on Ru@Cu-TM cathode, NiFe@NF anode and Sustainion X37-50 grade 60 and Fumasep FAA-3-PK AEMs were tested to evaluate the effect of different type of EL separators (diaphragm vs. AEM). A commercially available 5 cm^2^ AEM electrolyzers (Dioxide Materials) based on Sustainion® AEM, NiFe_2_O_4_@SSFF anode, and a cathode based on NiFeCo nanoparticles deposited onto a Sigracet 39BC CPR GDL was also tested to benchmark the performance of our AELs. Also, an AEL using a Pt/C cathode optimized in ref. ^71^ was fabricated and characterized to assess the long-term stability of our 5-stacked SSMs anode at 1 A/cm^2^ continuous operation. The AELs were connected to a custom-built station, which, through a peristaltic pump (Masterflex L/S Series), continuously supplies the anodic and cathodic half-cells with a 30 wt% KOH solution at a flow rate of 30 mL/min per cm^2^ of electrode area, at a temperature of 80 °C (controlled with a proportional-integral-derivative controller) and an atmospheric (1 bar) system pressure. Capillarity-fed AELs were also assembled using the same cell hardware above described, except using large diaphragms, the latter immersed in temperature-controlled electrolyte feedstock. The distance between the bottom part of the AEL electrodes and the surface of the electrolyte feedstock was fixed at 2.1 cm^49^, corresponding to the pathway length of the capillarity-induced transport of the electrolyte. Optionally, two nonwoven wipers were added as additional spacers, providing additional pathways for the capillarity-induced transport of the electrolyte beyond the one given by Zirfon Perl UTP 220 diaphragm. The electrolysis power was supplied by VMP3 Biologic potentiostat/galvanostat, equipped with an external high current booster channel. The potentiostat/galvanostat was used to perform LSV, CP, and EIS measurements. Polarization curves were acquired through CP sequences. The cell voltage was averaged over 2 min of each current step to provide a point of the polarization curve. The diaphragm/membrane resistance was obtained by means of EIS measurements, where the frequency was swept from 100 kHz to 1 Hz, with an AC signal amplitude of 10 mV around the open circuit potential. The diaphragm/membrane resistance was determined from the intercept of the real axis of the Nyquist plot at high frequencies. The durability of the AEL was assessed through CP measurements by fixing the current density at 1 A/cm^2^. The AEL station operated with separate electrolyte cycles, avoiding mixing of the anodic and cathodic electrolyte cycles of traditional AEL electrolysis, a practice recommended in previous reports^14^. This AEL operation management can limit the anodic hydrogen contamination, guaranteeing a safe operation without requiring extra measures (e.g., gas separating unit) to reduce the crossover or the hydrogen content within the anodic half-cell^14^. The energy efficiency of the AELs was calculated as below:

12 $${{{{{\rm{Energy\; efficiency}}}}}}=\frac{{{{{{{\rm{E}}}}}}}_{{{{{{\rm{output}}}}}}}}{{{{{{{\rm{E}}}}}}}_{{{{{{\rm{input}}}}}}}}=\frac{{{{{{{\rm{M}}}}}}}_{{{{{{\rm{H}}}}}}2}\times {{{{{\rm{HVV}}}}}}}{{{{{{{\rm{E}}}}}}}_{{{{{{\rm{input}}}}}}}}$$

In the expression above, M_H2_ is the hydrogen gas weight, HHV is the higher heating value of H_2_ (141.7 KJ/g H_2_), and E_input_ is the electric power consumed to produce the hydrogen, which can be calculated by multiplying the working power of electrolyzer by time. Details of calculations on H_2_ production rate, and its related cost can be found in the Supplementary Information. Though this efficiency metric is commonly used in literature for lab-scale electrolyzers, E_input_ neglects some energy input contributions, including energy consumption from water peristaltic pumps and thermal energy input. Therefore, our energy efficiency metric represents an approximated form of the real energy efficiency used for industrial electrolyzers (Note: despite our approximation used for energy efficiency metrics, the TEA used to estimate the cost of hydrogen include the thermal energy input, as detailed in Supplementary Information).

The voltage efficiency of the AELs was calculated as:

13 $${{{{{\rm{Voltage}}}}}}\; {{{{{\rm{efficiency}}}}}}=\frac{{{{{{\rm{Thermodynamic}}}}}}\; {{{{{\rm{voltage}}}}}}\, ({{{{{\rm{V}}}}}})}{{{{{{\rm{Operating}}}}}}\; {{{{{\rm{voltage}}}}}}\, ({{{{{\rm{V}}}}}})}$$

where thermodynamic voltage is the ideal voltage for liquid water splitting under our operating conditions (i.e., 80 ^o^C, 1 bar), which could be approximately computed using the following expressions^72^:

14 $${{{{{\rm{Thermodynamic}}}}}}\; {{{{{\rm{voltage}}}}}}\,({{{{{\rm{V}}}}}})=1.4736-0.8212\times {10}^{-3}\times {{{{{\rm{T}}}}}}({{{{{\rm{T}}}}}}\; {{{{{\rm{is}}}}}}\; {{{{{\rm{expressed}}}}}}\; {{{{{\rm{in}}}}}}\; {{{{{\rm{Kelvin}}}}}})$$

At 80 ^o^C (and 1 bar pressure), the thermodynamic voltage is calculated as 1.184 V.

The thermoneutral voltage for liquid water splitting is expressed as:

15 $${{{{{\rm{Thermoneutral}}}}}}\; {{{{{\rm{voltage}}}}}}\; ({{{{{\rm{V}}}}}})=1.5303-0.1646 \times {10}^{-3}\times {{{{{\rm{T}}}}}}({{{{{\rm{T}}}}}}\; {{{{{\rm{is}}}}}}\; {{{{{\rm{expressed}}}}}}\; {{{{{\rm{in}}}}}}\; {{{{{\rm{Kelvin}}}}}})$$

At 80 ^o^C (and 1 bar pressure), the thermoneutral voltage is calculated as 1.472 V.

### Technoeconomic analysis

The TEA of our AEL technology was performed to estimate the CAPEX, OPEX and H_2_ production cost of a single cell and of a corresponding ideal 1 MW-scale AEL plant. All calculations were run for each cathode-anode combination, depending on the data availability (as listed in Supplementary Table 13).

#### CAPEX of a single cell (US$/cm^2^)

The cost of the main components of the DEP were evaluated according to the standard commercial price of the raw materials composing each item (Supplementary Table 7). For cathodes and anodes, the final price of production also considers the manufacturing process; relevant operational parameters and related calculations can be found in the Supplementary Table 8. Additional cost of cell assembly was regressed from literature available data^51^, as described in the following.

#### CAPEX of an ideal 1 MW-scale electrolyzer (US$/MW)

The CAPEX of an ideal 1 MW-scale AEL was estimated starting from data provided by IRENA^51^ and reports on currently operating large-scale AEL plants^52^. Considering average cost of AEL stacks and the related cost breakdown reported by IRENA^51^, the price of each component of the AEL was retrieved, including cell manufacture and Balance of Plant entries. According to operative parameters of a 1 MW AEL plant from the Korea Institute of Energy Research (KIER), an ideal scale-up of our 5 × 5 cm^2^ to *~*700 cm^2^ single cells was carried out. For a fair comparison, the number of stacks (5) and cells (1000, 200 per stack) was kept constant, while the single cell active area was sized to meet the 1 MW effective power of the AEL plant. Then, assuming a linear proportionality between the cost of cathodes/anodes (including both raw materials and fabrication costs) with size and summing up the CAPEX of each up-scaled DEP component, the overall CAPEX of single DEPs was calculated for each tested cathode/anode combination. All data and calculations are reported in the Supplementary Information (Supplementary Tables 7–10). Annual H_2_ production estimation: the amount of yearly produced H_2_ (${{{{{{\rm{kg}}}}}}}_{{H}_{2}}$/year) by the 1 MW AEL plant was estimated from the data collected at the laboratory-scale, assuming the HER and OER performances measured for our AELs. The total current delivered by the system in 1 year (I) was calculated considering the number of cells/stacks and active electrode area obtained from the previously discussed CAPEX calculations:

16 $${{{{{\rm{I}}}}}}={{{{{\rm{j}}}}}}\times {{{{{{\rm{A}}}}}}}_{{{{{{\rm{el}}}}}}}\times {{{{{{\rm{n}}}}}}}_{{{{{{\rm{cells}}}}}}\; {{{{{\rm{per}}}}}}\; {{{{{\rm{stack}}}}}}}\times {{{{{{\rm{n}}}}}}}_{{{{{{\rm{stacks}}}}}}\; {{{{{\rm{per}}}}}}\; {{{{{\rm{system}}}}}}}$$

where *j* is the current density (A/cm^2^) and A_el_ is the single cell electrode area (cm^2^).

From the total current delivered, the amount of H_2_ produced by the AEL plant per year was calculated through Faraday’s laws:

17 $${{{{{\rm{Annual}}}}}}\, {{{{{{\rm{H}}}}}}}_{2}\, {{{{{\rm{production}}}}}}=\frac{{{{{{\rm{I}}}}}}\times {{{{{\rm{t}}}}}}\times {{{{{\rm{FE}}}}}}\times {{{{{{\rm{MM}}}}}}}_{{{{{{\rm{H}}}}}}2}}{{{{{{\rm{n}}}}}}\times {{{{{\rm{F}}}}}}}$$

where t is the time, FE is the Faradaic efficiency, MM_H2_ is the molecular mass of hydrogen (g/mol), n is the number of electrons transferred for each H_2_ molecule generated (mol_e-_/mol_H2_) and F is the Faraday’s constant (C/mol_e-_). Conversion factors are omitted in the formulas for the sake of conciseness.

#### OPEX estimation

Operational expenditures were estimated for the AEL plants considering the cost of electricity, labor, water consumption, maintenance and general ancillary costs. Following previously reported guidelines for the estimation of AEL plant OPEX, labor, maintenance and ancillary costs were evaluated in terms of percentages of initial CAPEX. Electricity and water-related costs were calculated according to the electrochemical performance of our AELs and the corresponding amount of produced hydrogen. Details of calculations are available in the Supplementary Information.

#### Calculation of H_2_ production cost

The production cost of hydrogen was evaluated according to the following equation:

18 $${{{{{{\rm{H}}}}}}}_{2}\, {{{{{\rm{production}}}}}}\; {{{{{\rm{cost}}}}}}\left({{{{{\rm{US\$}}}}}}/{{{{{{\rm{kg}}}}}}}_{{{{{{{\rm{H}}}}}}}_{2}}\right)=\frac{{{{{{\rm{Annual}}}}}}\; {{{{{\rm{CAPEX}}}}}}+{{{{{\rm{Annual}}}}}}\; {{{{{\rm{OPEX}}}}}}}{{{{{{\rm{Annual}}}}}}\, {{{{{{\rm{H}}}}}}}_{2}\, {{{{{\rm{production}}}}}}}$$

in which annual OPEX was calculated considering the current delivered by AEL plant in 1 year of operation, the annual CAPEX was calculated from overall CAPEX considering its depreciation through a capital recovery factor (CRF), i.e.:

19 $${{{{{\rm{CRF}}}}}}=\frac{{{{{{{\rm{i}}}}}}}_{{{{{{\rm{Rate}}}}}}}\times {(1+{{{{{{\rm{i}}}}}}}_{{{{{{\rm{Rate}}}}}}})}^{{{{{{\rm{n}}}}}}}}{{(1+{{{{{{\rm{i}}}}}}}_{{{{{{\rm{Rate}}}}}}})}^{{{{{{\rm{n}}}}}}}-1}$$

where i_Rate_ is the discount rate and n is the AEL plant lifetime.

#### Ru availability evaluation

We carried out a preliminary evaluation of the required Ru mass per deployed AEL power (g_Ru_/kW_AEL_) and, expanding from that, we roughly estimated the alleged Ru demand to cover the entire worldwide H_2_ production envisaged by IRENA’s Planned and Transforming Energy Scenarios^51^. Setting the bar at a global 15 TW power by 2050 (IRENA-Transforming Energy Scenario^51^) and assuming (i) a linear yearly increase in the water electrolyzers’ deployed power and (ii) the whole H_2_ production to be covered by AELs running on our technology.

### Supplementary information

Supplementary Information

Description of Additional Supplementary Files

Supplementary Data 1

Supplementary Data 2

### Source data

Source Data

## Data Availability

All data needed to support the findings in the paper are presented in the paper and the Supplementary Information (PDF and Excel files). Source data are available. Source data are provided with this paper.
