# Supplementary material for: High-performance alkaline water electrolyzers based on Ru-perturbed Cu nanoplatelets cathode
Source: Nat Commun. 2023 Aug 4;14:4680. doi: 10.1038/s41467-023-40319-5 (PMC10403570; doi:10.1038/s41467-023-40319-5)
Supplement: Supplementary file 1 — Supplementary Information [file 41467_2023_40319_MOESM1_ESM.pdf]

Supplementary Information

High-performance alkaline water electrolyzers based on Ru-perturbed Cu nanoplatelets cathode

Yong Zuo, Sebastiano Bellani, Michele Ferri, Gabriele Saleh, Dipak V. Shinde, Marilena Isabella Zappia, Rosaria Brescia, Mirko Prato, Luca De Trizio, Ivan Infante, Francesco Bonaccorso, and Liberato Manna

Contents

Abbreviations 2

Supplementary investigations of the Ru@Cu-TM electrodes 3

Experimental data on hydrogen adsorption free energy 15

Investigations of anodes 16

Supplementary investigations of AELs 21

Supplementary Table 1. Comparison between the HER activity of various PGM-based catalysts in 1 M KOH/NaOH reported in the past 5 years. 35

Supplementary Table 2. Free energies on hydrogen desorption on pure metals. 38

Supplementary Table 3. Water dissociation energies on Cu and Ru. 38

Supplementary Table 4. Comparisons between the OER activity of Ni- and/or Fe-based catalysts in 1 M KOH/NaOH reported in the past 5 years 39

Supplementary Table 5. Comparison between overall water splitting voltages at 10 mA/cm^2 measured for various catalysts in 1 M KOH/NaOH reported in the past 5 years. 40

Supplementary Table 6. Comparison between the water splitting performances of our AELs and those of ELs reported in the past 5 years. 41

Calculation of mass and price activities 48

Estimation of operating cost for H_2 production in our AELs 49

Techno-economic analysis of H_2 production at MW-scale AEL plant-level 50

Supplementary Table 7. Cost of raw materials composing the DEP. References for cost determination can be found in the supplemental Excel file. 52

Supplementary Table 8. Operational parameters for cathode and anode manufacturing and related cost of reagents and energy 53

Supplementary Table 9. Operational parameters of the KIER AEL plant reported by Lee et al. 54

Supplementary Table 10. Miscellaneous data used for the TEA 54

Supplementary Table 11. Unitary cost ($/cell) of a single cell of *ca.* 700 cm^2 for different cell configurations. 55

Supplementary Table 12. Potential (V) required by different single cells to deliver a 1 A/cm^2 current density 59

Supplementary Table 13. List of possible cathode/anode combination determining the DEPs 59

Supplementary Note 1. Comments on PGM-based electrocatalysts for HER 60

Supplementary Note 2. Comments on comparison between the water splitting performances of our AELs and those of ELs 60

Supplementary Note 3. Comments on the different trends of performance of SSM and NiFe@NF as anode in three-electrode configuration and AEL conditions 60

Abbreviations

The acronyms used in the text are defined here below.

AEL (alkaline water electrolyzer); **AEM-EL** (anion-exchange membrane electrolyzer); **BoP** (Balance of Plant); **CA** (chronoamperometry); **CAPEX** (capital expense); **CE** (counter electrode); **CFR** (capital recovery factor); **CP** (chronopotentiometry); **CPR GDL** (carbon paper gas diffusion layer); **CuO NPLs** (copper oxide nanoplatelets); **CV** (cyclic voltammetry); **DEP** (Diaphragm/electrodes package); **ECSA** (electrochemical surface area); **EDS** (energy dispersive X-ray spectroscopy); **EELS** (electron energy loss spectroscopy); **EIS** (electrochemical impedance spectroscopy); **EL** (electrolyzer) **EPDM** (Ethylene Propylene Diene Monomer) **GDL** (gas diffusion layer); **HAADF-STEM** (high-angle annular dark-field scanning transmission electron microscopy); **HER** (hydrogen evolution reaction); **ICP-OES** (inductively coupled plasma - optical emission spectroscopy); **LSV** (linear sweep voltammetry); **NF** (Ni foam); **NiFe@NF** (amorphous Ni-Fe hydroxide onto NF) **NM** (nickel mesh); **NPL** (nanoplate); **NPs** (nanoparticles); **NW** (nanowire) **OER** (oxygen evolution reaction); **OPEX** (operational expense); **PEM-EL** (proton-exchange membrane electrolyzer); **PGM** (platinum group metal); **PTFE** (polytetrafluoroethylene); **Pt@TFF** (platinized Ti fiber felt); **RHE** (reversible hydrogen electrode); **Ru@Cu-TM (**vertically oriented Cu NPL arrays grown on TM); **SEM** (scanning electron microscopy); **SSFF** (stainless steel fiber felt); **SSM** (stainless steel mesh); **TEA** (techno-economic analysis); **TEM** (transmission electron microscopy); **TM** (Ti mesh); **TOF** (turnover frequency); **TRL** (Technology Readiness Level); **xTi@NM** (NM coated by a sputtered Ti layer with a thickness of x nm), **xTi@SSM** (SSM coated by a sputtered Ti layer with a thickness of x nm); **XPS** (X-ray photoelectron spectroscopy); **XRD** (X-ray diffraction).

Supplementary investigations of the Ru@Cu-TM electrodes

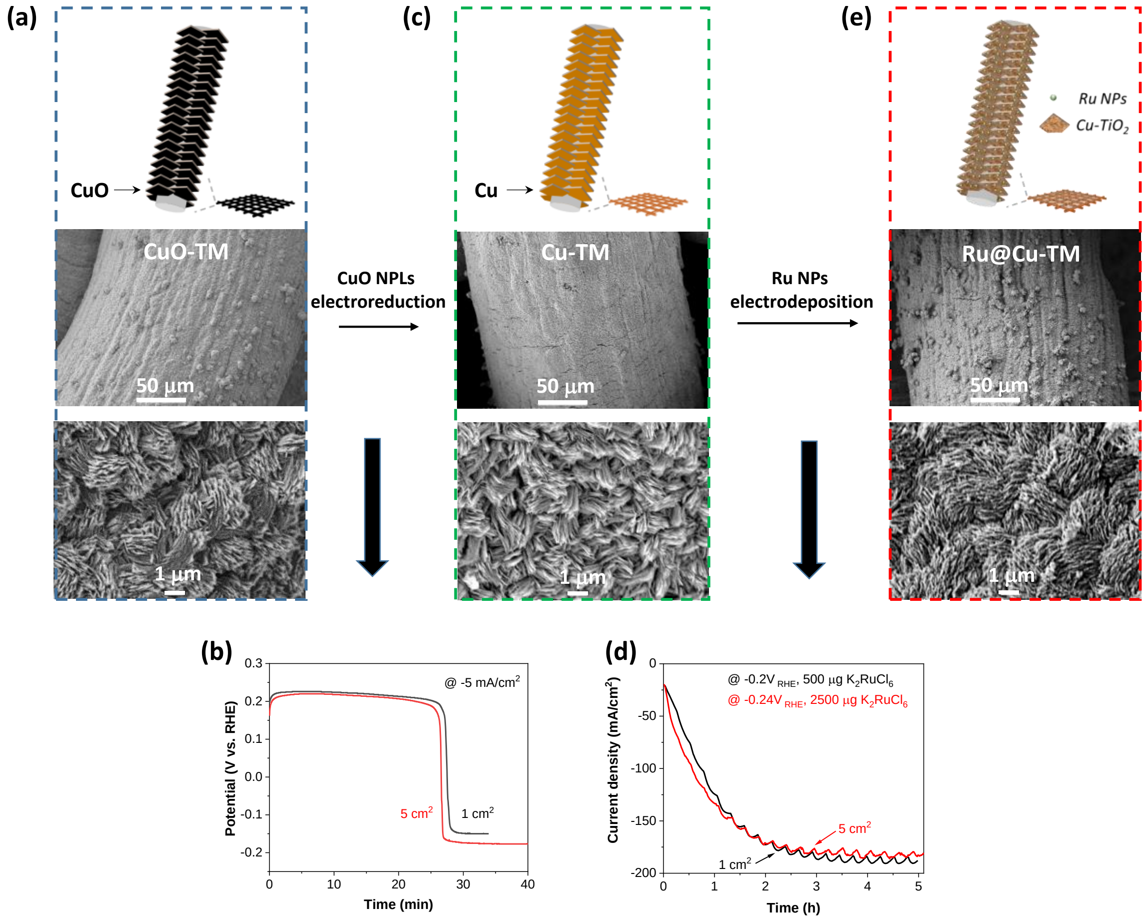

Supplementary Fig. 1. Synthesis of Cu-TM and Ru@Cu-TM. (a) Scheme and SEM image of the CuO NPLs on the TM substrate (CuO-TM) using chemical bath deposition; (b) Chronopotentiometric potential vs. time plots at -5 mA/cm^2, showing the electroreduction of CuO NPLs to Cu NPLs; (c) Scheme and SEM image of reduced Cu NPLs on the Cu substrate (Cu-TM); (d) Chronoamperometric current density as a function of time at applied potentials (-0.20 V vs. RHE and -0.24 V vs. RHE for 1 cm^2- and 5 cm^2-geometric area electrodes, respectively); (e) Scheme and SEM image of the Ru@Cu-TM electrode. Ru NPs deposition conditions for 1 cm^2 electrode: 25 mL electrolyte volume, 500 μg K_2RuCl_6 in the electrolyte bath. For 5 cm^2 electrode: 100 mL electrolyte volume, 2500 μg K_2RuCl_6 in the electrodepostion bath.

The amount of Cu NPLs onto TM surface from its CuO precursor can be calculated using the following equation:

$$\text{molar of Cu} = \left(\frac{j}{1000} \times t \times 60\right) / (F \times n),$$

where j, expressed in mA/cm^2, is the current density used to reduce CuO into Cu shown in the plot of **Supplementary Fig. 1b** (i.e., 5 mA/cm^2); t, expressed in min, is the time for CuO reduction to Cu; F is the Faraday's constant (96485 C/mol) and n is the number of transferred electrons that reduced one CuO molecule to Cu (i.e., n = 2).

For the 1 cm^2 electrode shown in **Supplementary Fig. 1b**, the completion of CuO reduction is around 30 min, therefore the mass of Cu NPLs on TM is:

$$\text{mass of Cu NPLs} = (\text{Mw of Cu}) \times (\text{Molar of Cu}) = 0.0988 \times t\ (\text{mg/cm}^2) = 0.0988 \times 28 = 2.77\ \text{mg/cm}^2$$

The mass of Cu NPLs calculated using the abovementioned equation is close to the ICP result, with an error of *ca.* 5%.

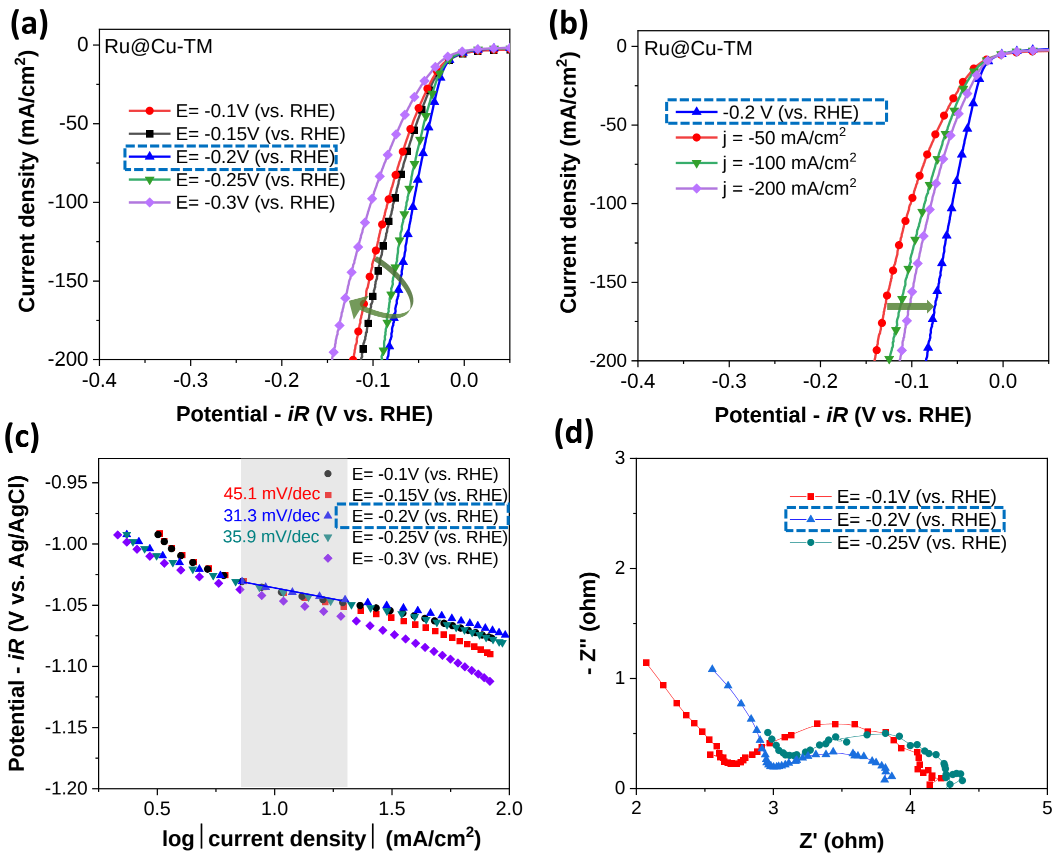

Supplementary Fig. 2. LSV curves with iR-correction measured for Ru@Cu-TM electrodes produced using different (a) potentials (E) and (b) current densities (j) for the electrodeposition of Ru NPs, and the comparison of their (c) Tafel plots and (d) EIS plots (acquired at -0.2 V vs. RHE). The dotted box in each Figure indicates the optimal parameter. Ru NPs deposition condition: 0.25 cm^2 electrode area, 50 mL electrolyte volume, 200 μg K_2RuCl_6 in the electrodeposition bath.

The Ru deposition in fabrication of the Ru@Cu-TM was carried out under different potentials (-0.1 V, -0.15 V, -0.2 V, -0.25 V, and -0.3 V vs. RHE, CA protocol) or current densities (-50 mA/cm^2, -100 mA/cm^2, and -200 mA/cm^2, CP protocol). The HER activity of the resulting Ru@Cu-TM electrodes has been evaluated by collecting LSV curves (**Supplementary Figs. 2a,b**), regressing Tafel plots (**Supplementary Fig. 2c**) and recording EIS spectra (**Supplementary Fig. 2d**). The most performant electrode was obtained by potentiostatic electrodeposition of Ru NPs at -0.2 V vs. RHE. Indeed, such electrode exhibited a HER overpotential (referred to 0 V vs. RHE) of 84 mV (56 mV) at current density of -200 mA/cm^2 (-100 mA/cm^2), a Tafel slope of 31.3 mV/dec, indicating fast HER kinetics determined by Tafel reaction, and a small semicircle (corresponding to a R_{ct} of 0.9 Ω) in EIS spectra (measured at -0.2 V vs. RHE), demonstrating the fastest charge transfer rate amongst the investigated electrodes.

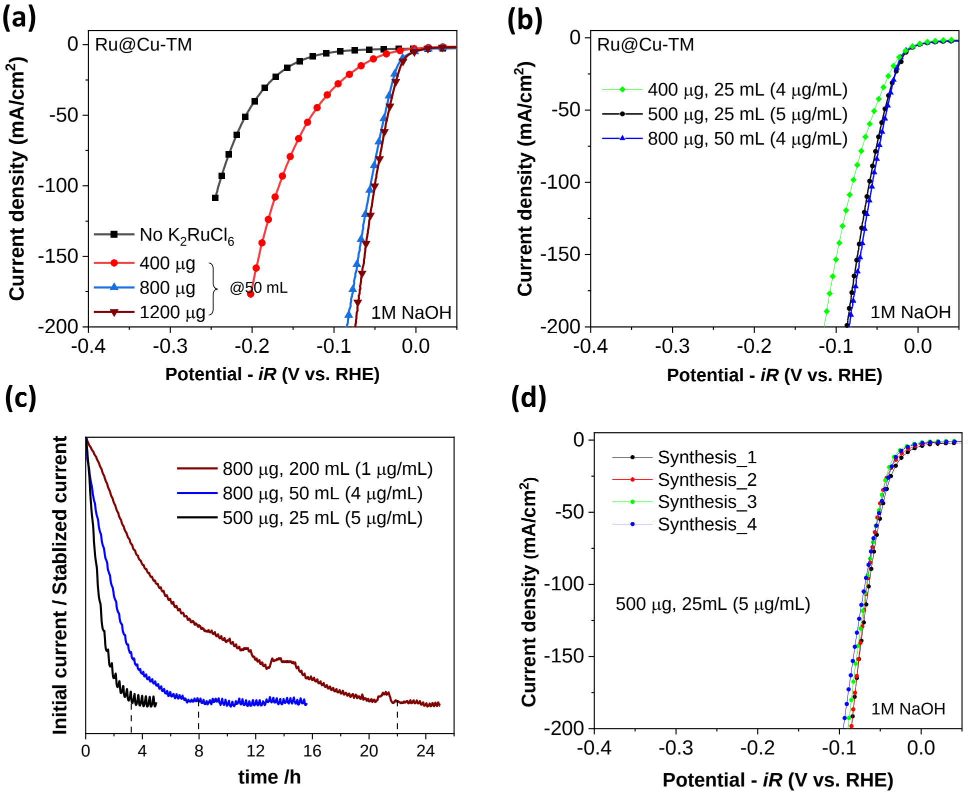

Supplementary Fig. 3. Optimization of the Ru precursor concentration in the electrolyte. (a) LSV curves with iR-correction measured for Ru@Cu-TM electrodes produced using different amounts of Ru precursor (K_2RuCl_6) in 50 mL of 1 M NaOH for the electrodeposition bath. (b) LSV curves with iR-correction measured for Ru@Cu-TM electrodes produced using different amounts of Ru precursor in 25 mL of 1 M NaOH electrodeposition bath, in comparison to the data obtained for the optimal electrodeposition condition found for a 50 mL bath volume. (c) CA plots showing the evolution of Ru deposition. The eventual stabilized current plateau demonstrates the completion of Ru deposition. (d) LSV curves with iR-correction measured on Ru@Cu-TM electrodes from four synthesis batches produced using 500 µg Ru precursor in 25 mL bath volume. The data have been acquired on electrode with an area of 1 cm^2. Values in brackets indicate the resulting concentration of Ru ion in electrolyte. The electrodeposition of Ru NPs was carried out at -0.2 V (vs. RHE).

Supplementary Figs. 3a,b show the iR-corrected LSV curves measured for Ru@Cu-TM electrodes produced by varying the amount of Ru precursor in the electrodeposition bath (50 mL and 25 mL of 1 M NaOH, respectively). As can be seen, the higher concentration of Ru leads to higher performance of the resulted electrode. Interestingly, **Supplementary Fig. 3b** demonstrated that by decreasing the volume of electrolyte (from 50 mL to 25 mL), the dosage of Ru precursor could be thus significantly decreased (from 800 µg to 500 µg), with the performance of resulting electrode being well-maintained.

Besides, **Supplementary Fig. 3c** shows that the higher concentration of Ru ions could result in a faster deposition procedure for Ru NPs. i.e., if 800 µg of K_2RuCl_6 is added to 50 mL electrolyte, corresponding to a 4 µg Ru ion/mL electrolyte, Ru deposition could be done in around 8 h, while 500 µg of K_2RuCl_6 in 25 mL electrolyte, corresponding to a 5 µg Ru ion/mL electrolyte, could be finished in only 3 h. Impressively, a similar catalytic performance towards HER have been seen on the produced electrodes. The above-presented protocol optimization on maximizing the concentration of Ru ion by minimizing the volume of electrolyte is beneficial in term of both electrode production rate and cost.

The LSV analysis in **Supplementary Fig. 3d** demonstrates the reproducibility of Ru@Cu-TM electrodes using our optimized protocol: -0.2 V (vs. RHE) for Ru deposition, 500 µg K_2RuCl_6 dosage, in 25 mL of 1 M NaOH electrolyte.

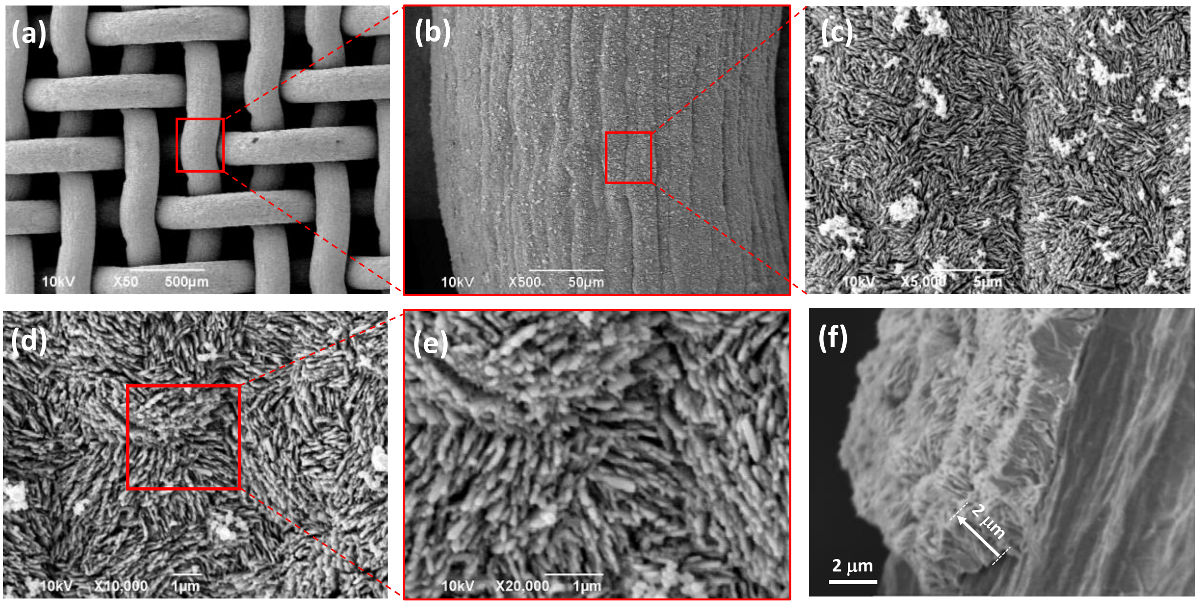

Supplementary Fig. 4. Additional SEM images of a representative Ru@Cu-TM. (a)-(e) Increasing magnification. (f) SEM image of tilted Ru@Cu-TM, showing the thickness of the catalyst layer (ca. 2 μm).

Supplementary Figs. 4a-e show the SEM images (at increasing magnification) of a representative Ru@Cu-TM, consisting of Ru NPs-decorated 3D nanostructured Cu NPL porous layer vertically grown onto the TM current collector. **Supplementary Fig. 4f** indicates that the thickness of the catalyst layer is *ca.* 2 μm.

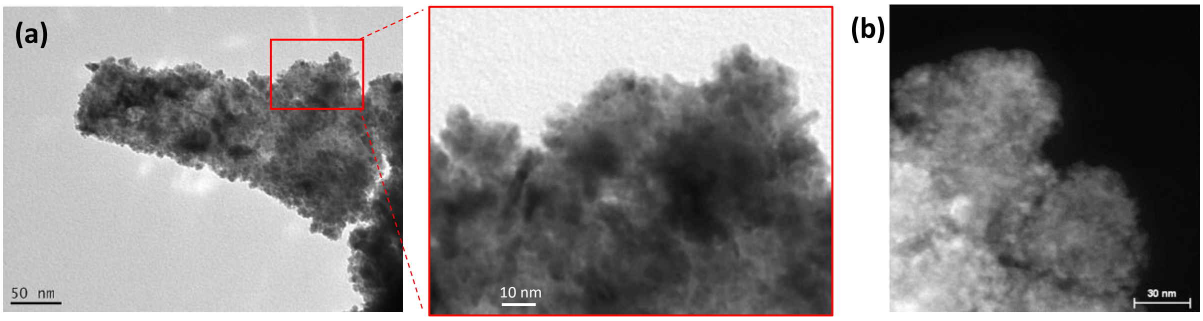

Supplementary Fig. 5. Ru@Cu catalyst powder removed and collected from TM substrate of Ru@Cu-TM. (a) TEM, (b) HAADF-STEM images

Supplementary Fig. 5a,b show the TEM images of the Ru@Cu catalyst powder, indicating the porous structure of the Cu matrix.

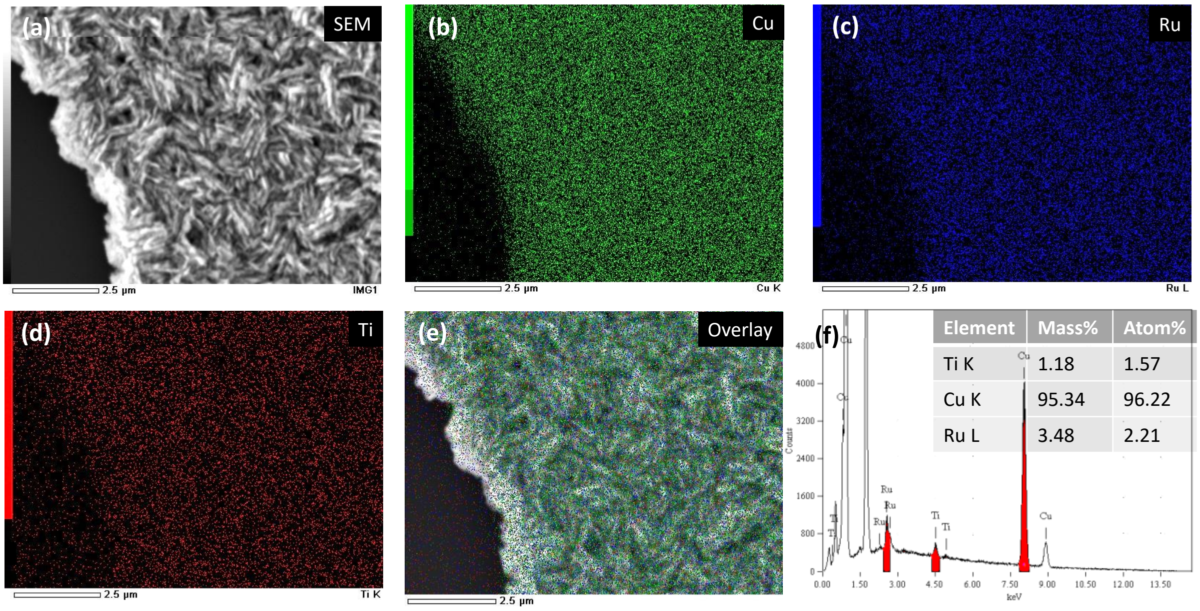

Supplementary Fig. 6. Determination of element distribution of fresh Ru@Cu catalyst powder collected from TM surface. (a) SEM and the corresponding EDS maps for (b) Cu, (c) Ru, (d) Ti and their (e) overlay. Ru@Cu catalyst powder was collected from TM substrate of Ru@Cu-TM and deposited on Si wafer. (f) EDS spectrum of the sample.

Supplementary Fig. 6 shows a SEM image of the Ru@Cu catalyst powder, together with its EDS maps for Cu, Ru and Ti, indicating a homogeneous element distribution, and the resulting EDS spectrum.

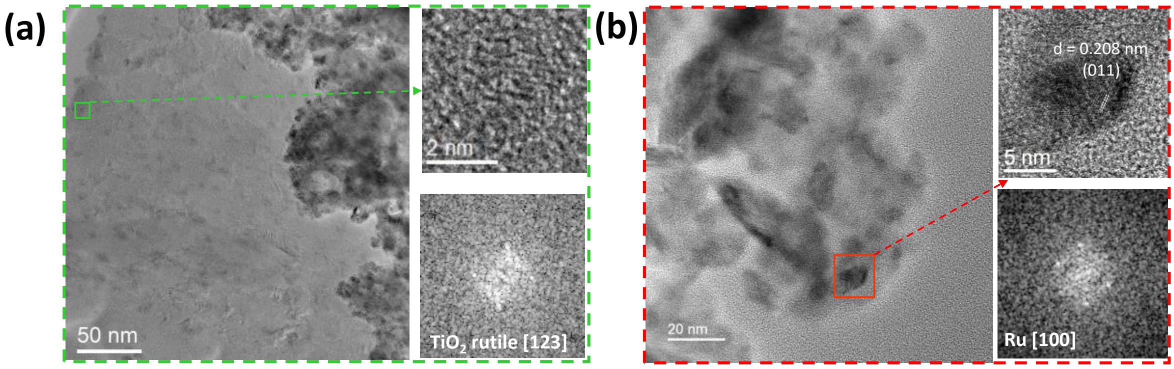

Supplementary Fig. 7. HRTEM characterization of Ru@Cu-TM. (a) HRTEM micrograph of a fragment of the electrode; magnification of the green-squared region and its fast Fourier transform (FFT), corresponding to the[123] zone axis of rutile TiO_2 (ICSD 16636). (b) HRTEM micrograph of a fragment with a Ru NP; detail of the red-squared region and corresponding FFT, corresponding to the [100] zone axis of Ru (ICSD 40354).

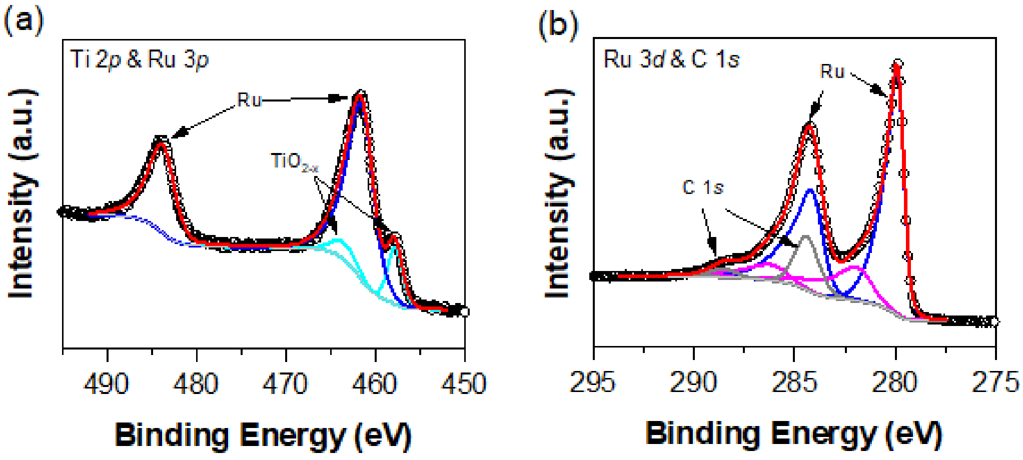

Supplementary Fig. 8. XPS characterization of the Ru@Cu-TM electrode focusing at energy ranges typical for (a) Ti 2*p* (overlaped with Ru 3*p*) and (b) Ru 3*d* (overlaped with C 1*s*) signals.

As shown in **Supplementary Fig. 8a**, Ti 2*p* peaks at 457.9 eV and 464.3 eV (cyan lines) were observed. Since the binding energy of these peaks is slightly lower than typical Ti(IV), the Ti species in our Ru@Cu-TM are likely attributed to TiO_{2-x}.[1] In **Supplementary Fig. 8b,** the the main peaks ascribed to metallic Ru (blue lines), while the presence of satellites are typically associated to RuO_2 specie (magenta lines). Notably, C1*s* peaks at 284.8 eV and 288 eV could be attributed to C-C, C-H and C=O groups, respectively.

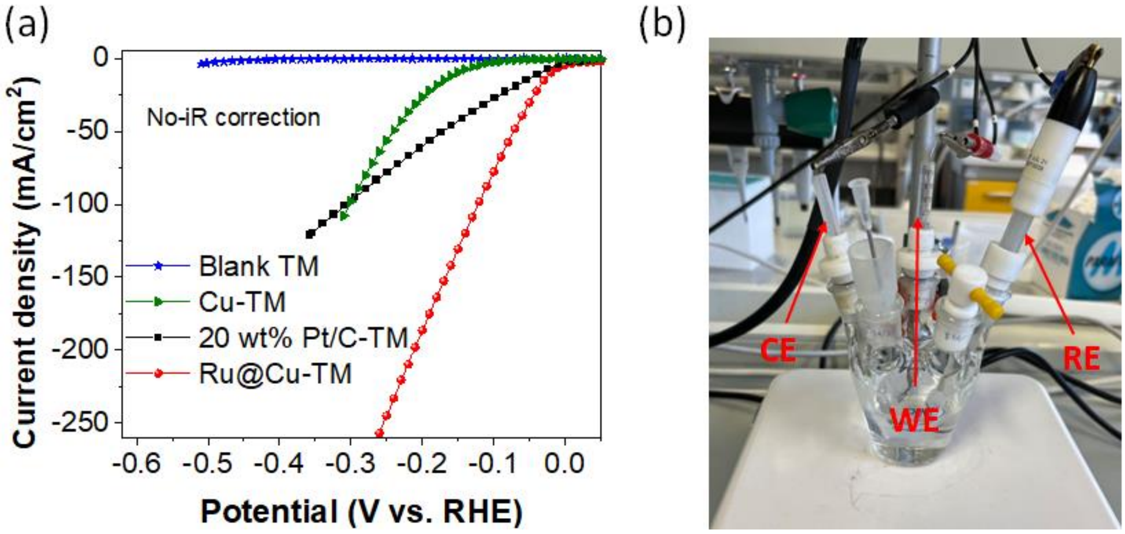

Supplementary Fig. 9. Recording of raw electrochemical data. (a) Non-iR corrected LSV curves measured for blank TM, Ru@Cu-TM and Pt/C-TM benchmark (100 $\mu g_{Pt}/cm^2$) deposited onto the same substrate (TM) in 1 M NaOH (electrode geometric area = 1 cm^2). (b) Set-up of three-electrode configuration used in this study. CE: counter electrode; WE: working electrode; RE: reference electrode.

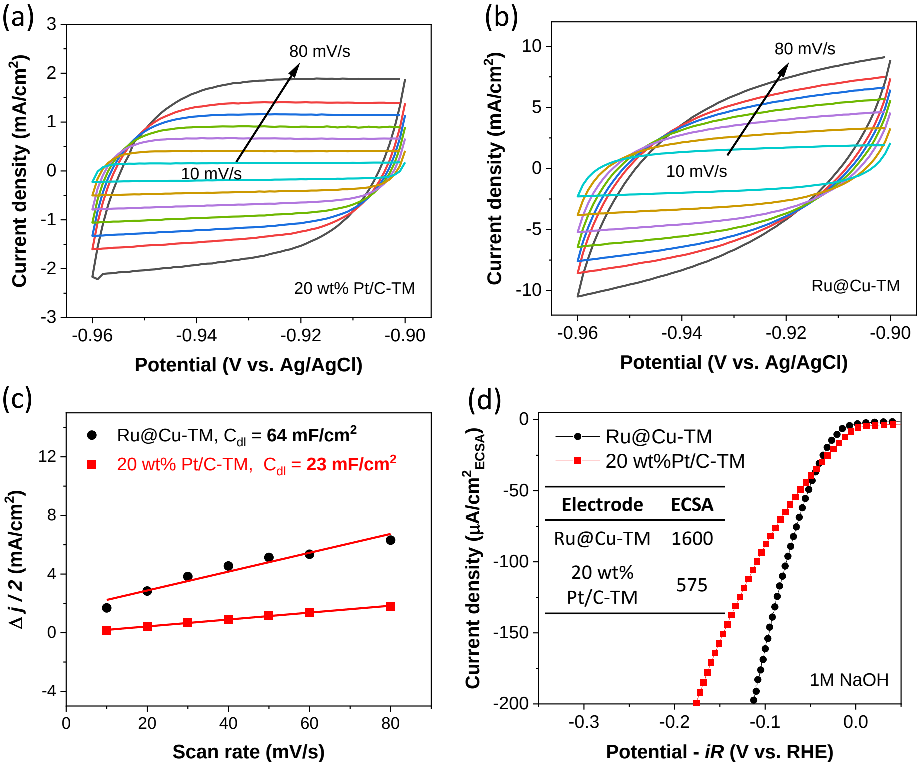

Supplementary Fig. 10. CV curves of (a) 20 wt% Pt/C-TM and (b) Ru@Cu-TM at scan rates of 10, 20, 30, 40, 50, 60, and 80 mV/s; (c) $\Delta j/2$ extracted from the corresponding CV curves at 0.93 V (vs. Ag/AgCl) as a function of the scan rates; (d) ECSA-normalized iR-corrected LSV curves.

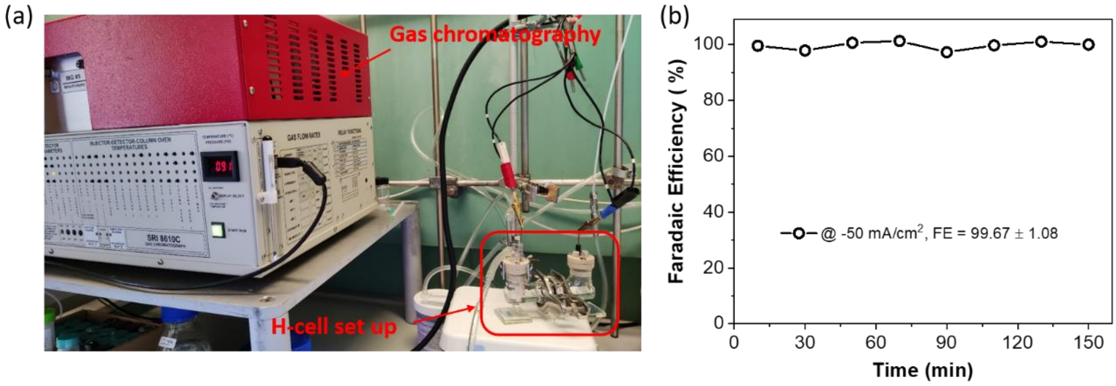

Supplementary Fig. 11. Operando quantification of Faradaic Efficiency of HER on Ru@Cu-TM electrode. (a) Overview of the apparatus, made of a gas chromatograph connected to the working electrode compartment side of a sealed H-cell. (b) Plot of the Faradaic Efficiency for the HER vs. time measured for Ru@Cu-TM operating at -50 mA/cm^2 current density. The average Faradaic efficiency is 99.67 %.

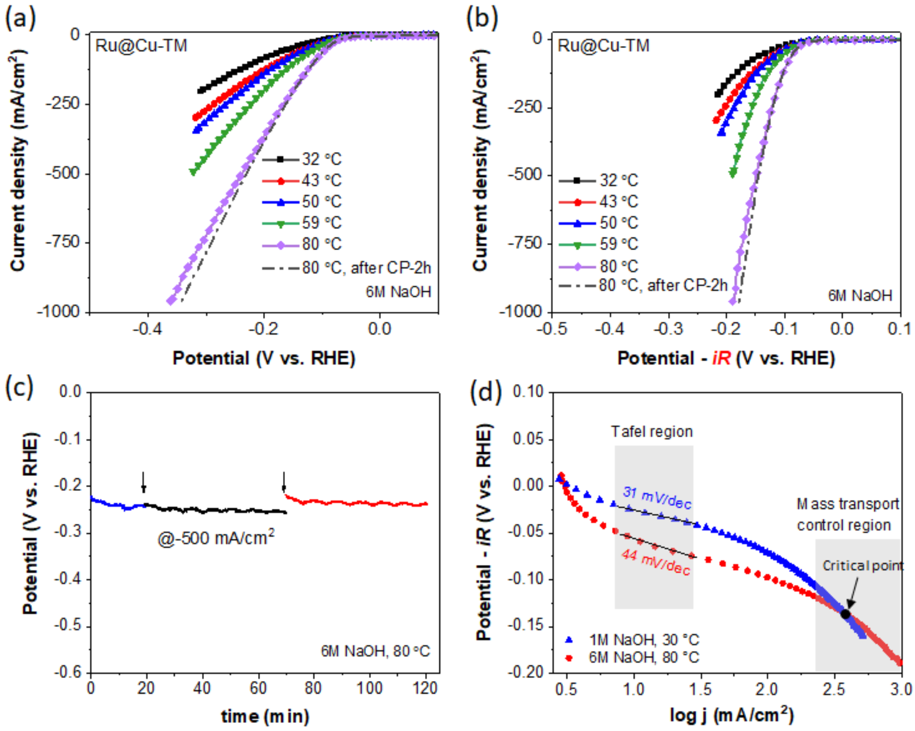

Supplementary Fig. 12. LSV curves with iR-correction measured for Ru@Cu-TM under simulated AEL conditions (6 M NaOH and temperature up to 80 ºC) (a) before and (b) after iR-correction. (c) CP measurements of Ru@Cu-TM operating at -500 mA/cm^2 in 6 M NaOH at 80 °C. Arrows indicate the time at which the water consumption is compensated. (d) Comparison between the Tafel curves measured for Ru@Cu-TM in 1 M NaOH at 30 °C and 6 M NaOH at 80 °C.

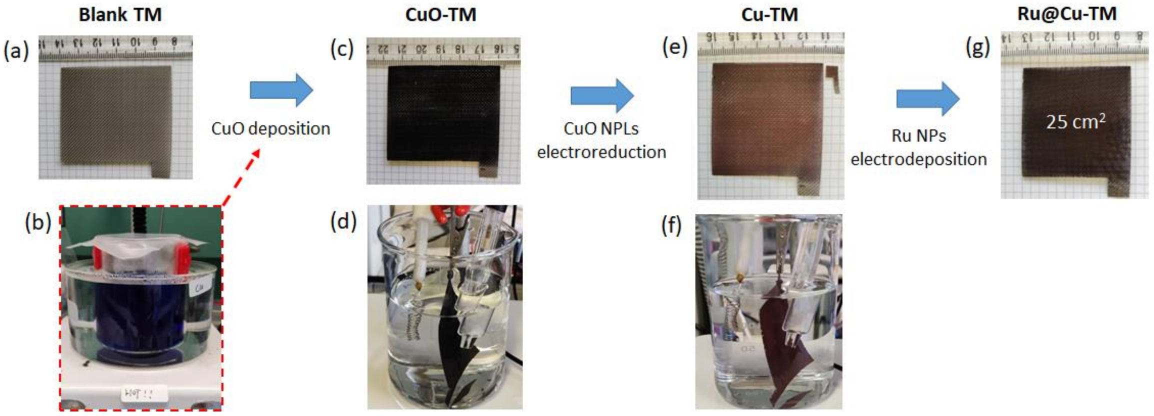

Supplementary Fig. 13. Scaling-up of the fabrication of Ru@Cu-TM up to a 25 cm^2 (geometric) area. The synthesis procedure is similar to the 1cm^2 size case. Typically, 10 mmol copper (II) chloride dehydrate was dissolved into 200 mL Milli-Q water in a beaker, and 10 mL ammonia solution (25 %) was then added dropwise to get a blue solution. After then, an "L" shape TM with a rectangular area of 6 cm × 5 cm (a) was vertically placed into the beaker, which was then immersed in a preheated water bath (stabilized at 90 ºC, as shown in b) for 3 h to obtain the CuO-TM electrode (c). The CuO-TM electrode was then immersed in 200 mL 1 M NaOH electrolyte and used as the working electrode in a three-electrode system (d), after which a negative current of -150 mA (-5 mA/cm^2) was applied until the electrode potential became stable, and bubbles (H_2) evolved from the electrode surface. A 1 cm × 5 cm area of the obtained Cu-TM electrode was cut and left for backup use (e). The remaining 5 cm × 5 cm Cu-TM was put back to the electrolyte and used as the working electrode again, and a multistep galvanostatic protocol was performed, shortly after adding 12.5 mL K_2RuCl_6 solution (1 mg/mL in water) into the electrolyte (f), to obtain Ru@Cu-TM (g). The currents employed in multistep galvanostatic were extracted uniformly at different time points from the chronoamperometric plot shown in Supplementary Fig. 1d (5 cm^2 case, displayed as current density). The selection covers its entire time period of 5 h.

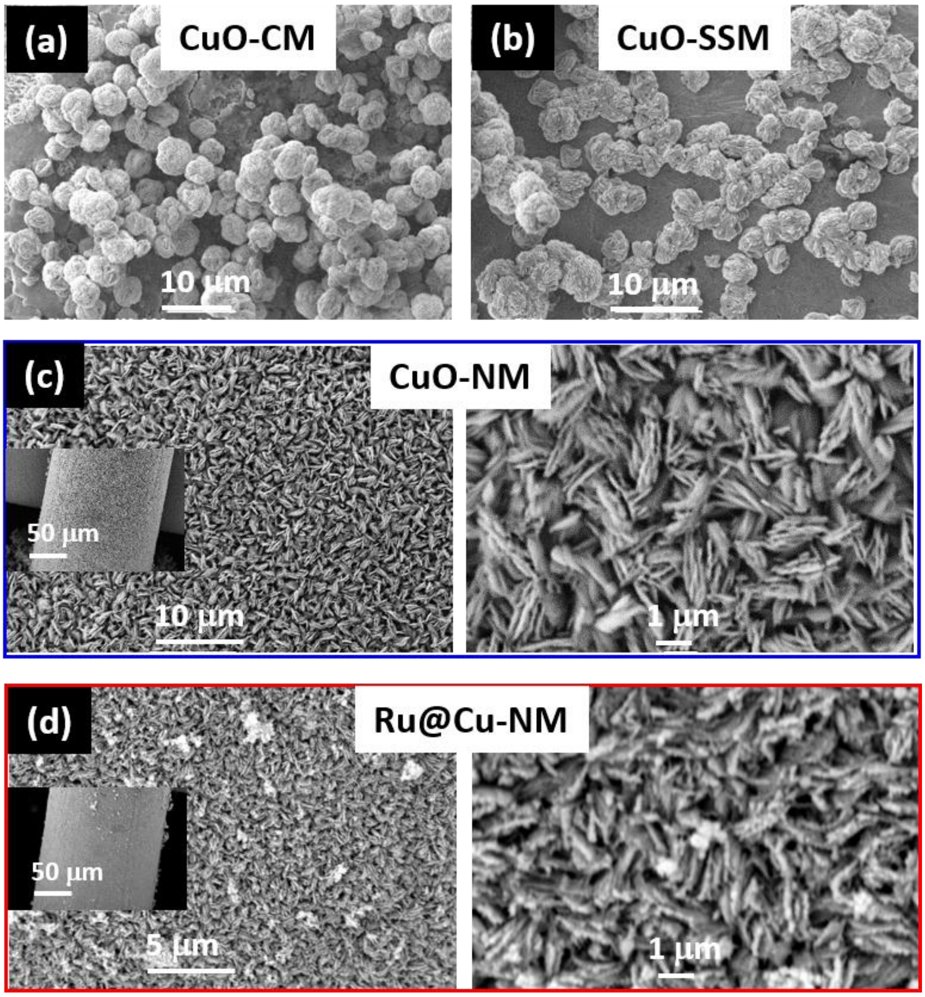

Supplementary Fig. 14. CuO NPLs grown onto (a) CM, (b) SSM, (c) NM *via* chemical bath deposition. (d) *in-situ* produced Ru@Cu-NM from CuO-NM. Only NM was coated with vertically aligned CuO NPLs as previously observed for TM substrate.

Both Cu mesh (CM) and stainless-steel mesh (SSM) resulted in the formation of separated CuO aggregates, leading to an insufficient substrate coverage. These results indicate that the CuO NPL growth is strongly affected by the chemistry (affinity) of the substrate surface.

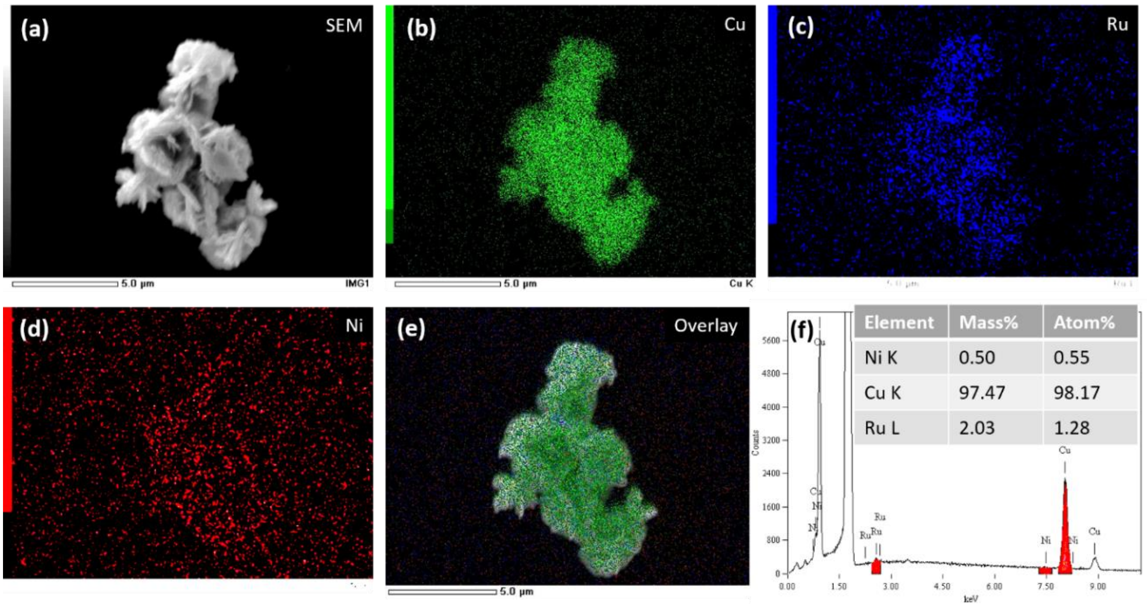

Supplementary Fig. 15. Determination of element distribution of fresh Ru@Cu catalyst powder collected from NM surface. (a) SEM image and the corresponding EDS map for (b) Cu, (c) Ru, (d) Ni and (e) and their overlay. Ru@Cu catalyst powder was collected using sonication and then deposited on Si wafer. (f) EDS spectrum of the sample.

Unlike Ti element being clearly observed in Ru@Cu catalyst grown on TM surface (**Supplementary Figs. 6,7**), the Ni element is hardly detected on Ru@Cu catalyst grown on NM, using SEM-EDS technique.

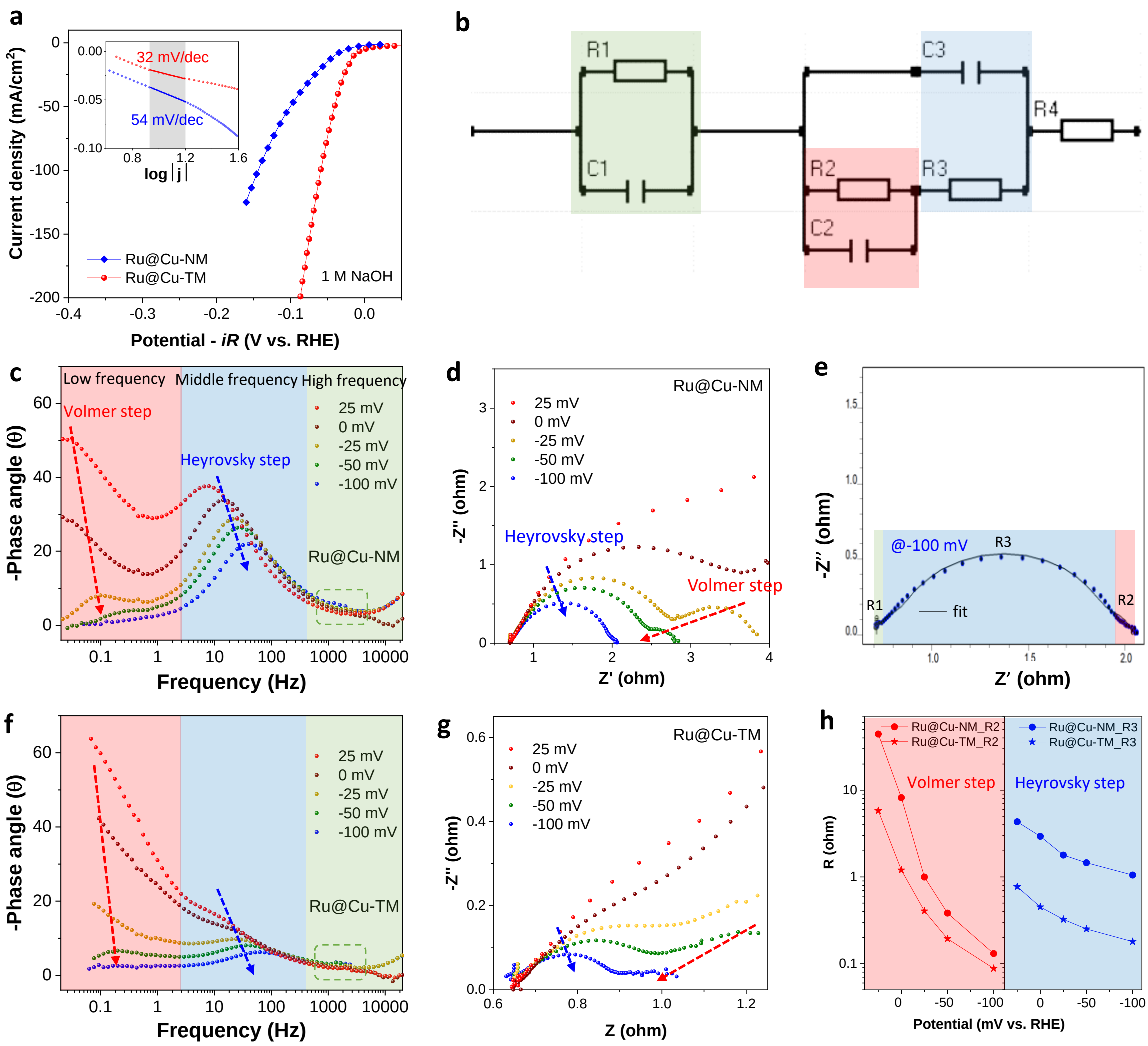

Supplementary Fig. 16. Investigation of HER kinetics on Ru@Cu-TM and Ru@Cu-NM cathodes. **a**, LSV curves with iR-correction. The Inset shows the corresponding Tafel plots. **b**, Equivalent electrical circuit used to fit EIS data measured for the cathode/electrolyte system. **c**, Bode plots and, **d**, corresponding Nyquist plots measured for Ru@Cu-NM at various overpotentials in 1 M NaOH. **e**, A fitting example extracted from (d). **f**, Bode plots and **g**, Nyquist plot measured for Ru@Cu-TM at various overpotentials in 1 M NaOH. **h**, Resistances (R2 and R3) of the equivalent electric circuit reported in (a) measured for Ru@Cu-NM and Ru@Cu-TM during operation in 1 M NaOH as a function of the potential vs. RHE.

As shown in **Supplementary Fig. 16a,** the Ru@Cu-NM cathode, showing similar structure of nanoplatelet arrays on the substrate surface (indicated in **Supplementary Fig. 14d)**, performed worse than the Ru@Cu-TM ones, which could be due to the incorporation of Ti species into catalyst. To understand the difference of HER kinetics of Ru@Cu based on TM and NM, EIS was performed under various overpotentials.

As is known, the HER proceeds through either Volmer-Heyrovsky or Volmer-Tafel mechanism or mix. The Volmer step ($H_2O + e^- \rightleftarrows H_{ads} + OH^-$, in which H_{ads} refer to adsorbed hydrogen) and Heyrovsky step ($H_2O + H_{ads} + e^- \rightleftarrows H_2 + OH^-$) involve one electron charge transfer, and therefore could be recorded by EIS technique, while no electron transfer occurs during Tafel step ($2H_{ads} \rightleftarrows H_2$). According to previous reports, the Volmer step (hydrogen adsorption at the catalyst surface) is associated to the low-frequency region. The medium/high (middle)-frequency region is related to the Heyrovsky step (charge transfer reaction at electrolyte-catalyst interface), and the high-frequency region is correlated to the electron transfer within catalyst inner-layer.[2,3] By referring to the electrical equivalent circuit for the cathode/electrolyte interface reported in **Supplementary Fig. 16b**, the resistance of the Volmer step

(R2), the resistance of the Heyrovsky step (R3) and the resistance of the electron transfer within the catalyst layer (R1) can be calculated as function of the HER overpotential. **Supplementary Fig. 16c,d** show the Bode plots and the corresponding Nyquist plots, respectively, measured for Ru@Cu-NM at various potentials in 1 M NaOH. The data are compared to those measured for Ru@Cu-TM (**Supplementary Fig. 16f,g**). As depicted in **Supplementary Fig. 16h**, compared to Ru@Cu-TM, Ru@Cu-NM displayed higher resistance for both the Volmer step (R2) and the Heyrovsky step (R3) within the investigated potential range (from 25 mV to -100 mV vs. RHE). This means that the TiO_2 species incorporated into the catalyst (see **Supplementary Figs**. **6-8**) can act as efficient water dissociation centers, in accordance with previous reports.[4,5] Regarding the Ru@Cu grown on NM, NiO is marginally incorporated into the catalysts (**Supplementary Fig. 15**) and does not actively participate in the Volmer step, as evidenced by the EIS analysis.

Experimental data on hydrogen adsorption free energy

Experimental adsorption energies and DFT overbinding. The heats of adsorption of H_2 (i.e. the adsorption enthalpies) were taken from ref. [6](Cu) and [7](Ru). To compare it with the DFT-calculated values, the zero-point energy correction (0.04 eV, as determined in ref. [8]) was subtracted, resulting in the following values for the adsorption energies: -0.17 eV (Cu) and -0.48 eV (Ru). The ε_{PBE} values (i.e., the DFT overbinding, see Methods section of the main text) were obtained as the difference between the abovementioned experimental values and the calculated ones (**Supplementary Table 2**). Note that for the hydrogen adsorption on Cu, a fairly wide range of experimental values was reported, depending on the structural details of the Cu film. The authors adopt as final values for the adsorption energies the range 40-50 kJ/mol (note that experimental values refer to the H_2 molecule, while the values discussed in our work are all normalized to a single H atom, i.e., the experimental values have to be halved to be compared to our simulations). We adopt the lower bound of that range because the experimental values obtained by other authors, discussed in ref. [6], are almost all lower than 40 kJ/mol.

Entropy of adsorption. While in the landmark paper by Nørskov et al.[8] the entropy of the adsorbed hydrogen was neglected, the analysis of experimental data in ref. [6] shows that that entropy reaches the considerable value of 60 J mol^{-1} K^{-1}, corresponding to an entropic contribution ($T\Delta S$) to the adsorption free energy at $T = 300$ K of 0.11 eV (normalized to ½ H_2, see previous paragraph) rather than 0.21 eV of ref. [8]. Note that, from ref. [6], we took the entropy value corresponding to the lowest hydrogen coverage, as the hydrogen coverage is low in our simulations as well (only one H atom is adsorbed in a large unit cell).

Investigations of anodes

To have a suitable anode benchmark for the evaluation of the AELs hereafter, we first developed an efficient OER catalyst taking inspiration from previous reports.[9,10] More in detail, the immersion of pre-cleaned NF in a solution of $Fe(NO_3)_3$ and $Ni(NO_3)_2$ at 80 °C was found to be a simple and effective method to grow OER-active Ni-Fe hydroxides with low crystallinity onto NF (NiFe@NF) (**Supplementary Figs. 17a,b**), capable to operate at current densities higher than 100 mA/cm^2 (see details of fabrication in Experimental Procedures). Various concentrations of Ni and Fe precursors, reaction temperature (50 °C and 80 °C) and treatment time (0.5 h, 1 h, 3 h and 6 h) were screened to evaluate their impact on the OER activity of the resulting electrodes. The latter are named NixFey_Xh@Y@NF, in which x and y indicate the Ni and Fe precursor concentrations, respectively, (expressed in mM), X refers to the treatment time (expressed in h), and Y indicates the reaction temperature (expressed in °C). As shown from the analysis of the OER overpotentials (referred to 1.23 V vs. RHE) at a fixed current density (**Supplementary Fig. 17c**), the Ni50Fe50_3h@80@NF was found to be the most performant anode. The SEM-EDS analysis demonstrates that Ni, Fe, and O elements were uniformly dispersed throughout the catalyst (**Supplementary Fig. 18**), with the atomic ratio of Ni/Fe being 2.17/1, nearly consistent with the results from ICP analysis (1.95/1). The XRD analysis, in turn, was characterized by a broad and weak diffraction peak at ~11.5° indicating the amorphous nature of the NiFe hydroxide (**Supplementary Fig. 19**).

Electrochemical measurements indicated that our optimized anode (hereafter simply named NiFe@NF) exhibited a current density of 200 mA/cm^2 at an overpotential as low as 267 mV, and worked at such current density for 200 h with only ~25 mV overpotential increase (**Supplementary Fig. 17d,e**). Notably, NiFe@NF did not show any significant morphology change after the CP test, further proving its electrochemical stability under high-current density operating conditions (**Supplementary Fig. 17f**). In addition, the inset SEM in **Supplementary Fig. 17f** indicated that the thickness of the NiFe layer on NF was ~2 µm. After the CP test, the XRD pattern of the NiFe powder collected from the NF surface (**Supplementary Fig. 19**) was analogous to that of the starting catalyst. Meanwhile, the EDS analysis revealed a partial Fe loss (~11 %), with the atomic ratio of Ni/Fe slightly increased to 2.42/1 (**Supplementary Fig. 20**), in agreement with the data obtained by the ICP analysis (2.27/1). A similar Fe leaching from the catalyst during OER operation was also reported in previous works, investigating the stability of NiFe-(oxy)hydroxide anodes in industrially relevant environments.[11] Notably, the NiFe@NF changed its color from the initial brownish to black after performing the OER (**Supplementary Fig. 21a**). We associate this effect to the oxidation of NiFe hydroxide to NiFe (oxy)hydroxide.[12]

The performance of our NiFe@NF anode was then compared with those of NF, single or stacked SSM, as well as with the literature and/or commercially viable benchmarks, i.e., hierarchically structured Ni-Fe on NF[13] and $NiFe_2O_4$ particles on a 316L sintered stainless steel fiber felt ($NiFe_2O_4$@SSFF)[14]. In addition, NiFe grown on NM by replacing NF was also evaluated to understand the effect from substrate structure. As shown in **Supplementary Figs. 17g,h**, our optimized NiFe@NF had the highest OER activity amongst the investigated anodes, and could outperform most of previously reported Ni-Fe-based OER electrocatalysts, especially at current densities higher than 100 mA/cm^2 (**Supplementary Fig. 17i**, **Supplementary Table 4**). Lastly, we demonstrated that the upscaling procedure does not affect the geometric activity of the NiFe@NF anode (**Supplementary Fig. 21b**), confirming its suitability as benchmark anode for AELs.

When compared to cathode/anode combinations reported previously for water electrolysis, the Ru@Cu-TM / NiFe@NF pair is expected to reach current densities of 10 and 200 mA/cm^2 at cell voltages of 1.45 and 1.58 V, respectively, in 1 M NaOH and at ~ 27 °C. Even if not of practical interest for AELs (whose characterization is reported hereafter), the cell voltage at 10 mA/cm^2 current density is one of the smallest ones among those reported in literature for cathode/anode pairs potentially considered for the design of high-efficiency AELs (**Supplementary Fig. 22** and **Supplementary Table 5**).

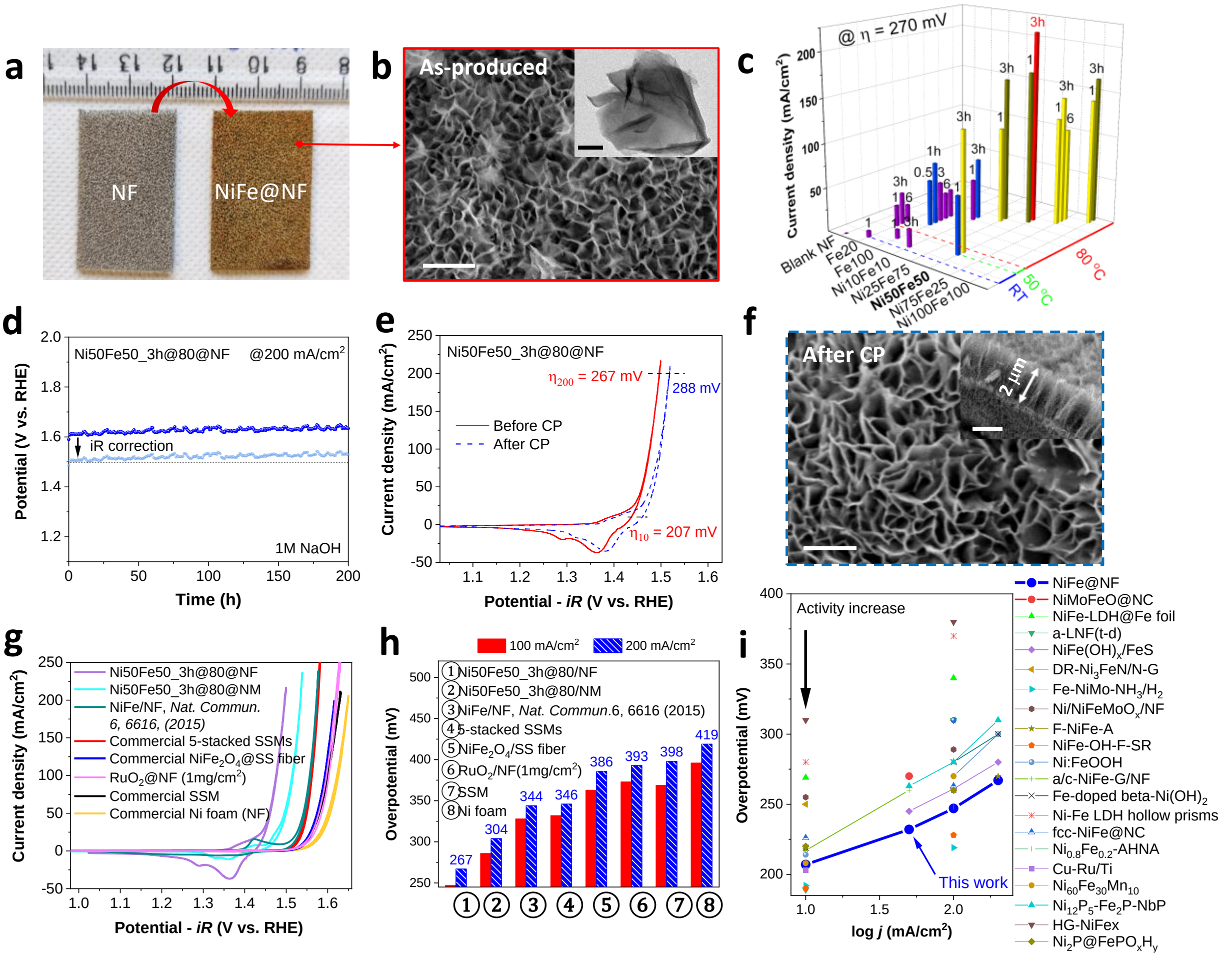

Supplementary Fig. 17. Characterization of the anode at the electrode-level. (a), Photograph showing the fabrication of NiFe@NF from blank NF. (b), SEM and TEM images of produced Ni50Fe50_3h@80@NF. Scale bars: 2 μm (main panel), 50 nm (inset panel). (c), Current density at overpotential equal to 270 mV measured for our anodes. The data have been extracted from the iR-corrected CV curves measured for anodes produced from different concentration ratios of Ni and Fe precursors, reaction temperature and treatment time; (d), Chronopotentiometric potential vs. time plot measured for Ni50Fe50_3h@80@NF at 200 mA/cm^2; (e), CV curves with iR-correction recorded from Ni50Fe50_3h@80@NF before and after the CP test at a potential scan rate of 2 mV/s; (f), SEM image of a representative Ni50Fe50_3h@80@NF after the CP test. The inset panel shows a SEM images of the tilted anode, indicating a thickness of the catalyst layer around 2 μm. Scale bars: 2 μm. (g), CV curves with iR-correction measured for our optimized NiFe@NF anode (i.e., Ni50Fe50_3h@80@NF) in comparison to those measured for commercial single and stacked SSM, NF, commerically viable $NiFe_2O_4$@SSFF[14], RuO_2 deposited onto NF (prepared by us), and literature benchmark (replicated by us) consisting of hierarchically structured Ni-Fe on NF.[13] (h), Overpotential of the investigated anodes for the OER at 100 mA/cm^2 (red hystograms) and 200 mA/cm^2 (blue hystograms). (i), Comparison between the OER activities of the Ni50Fe50_3h@80@NF and other Ni-Fe-based electrocatalysts for OER under 1 M KOH/NaOH reported in literature, as listed in **Supplementary Table 4**.

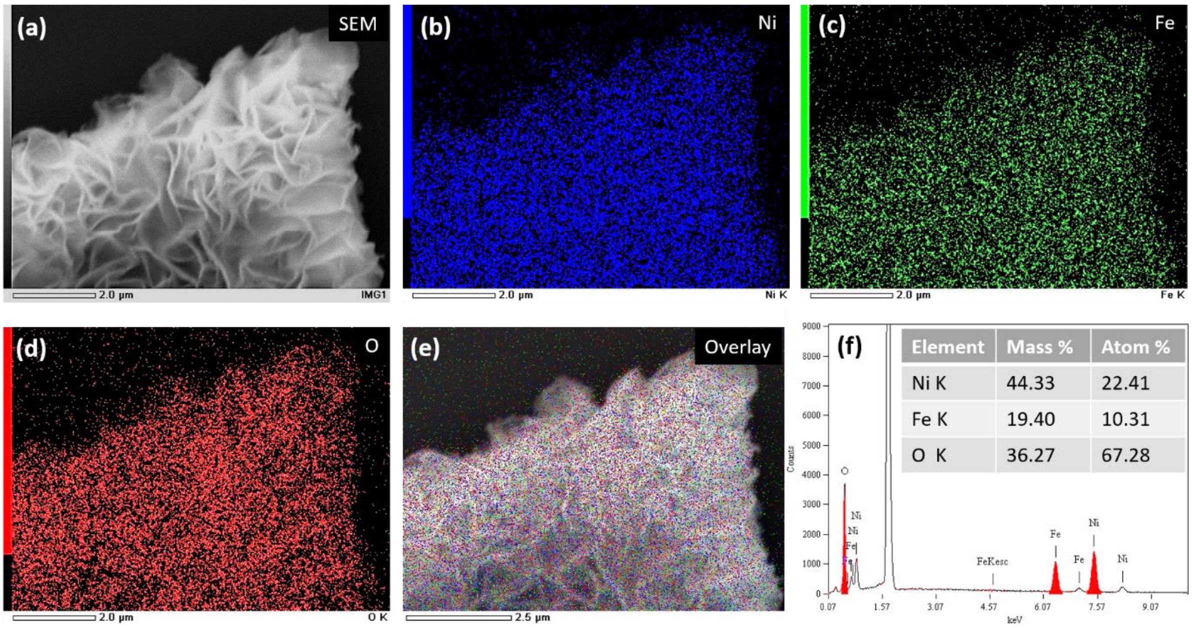

Element	Mass %	Atom %
Ni K	44.33	22.41
Fe K	19.40	10.31
O K	36.27	67.28

Supplementary Fig. 18. Determination of element distribution of fresh Ni50Fe50_3h@80C catalyst powder collected from NF surface. (a) SEM image and the corresponding EDS maps for (b) Ni, (c) Fe, (d) O and (e) overlay. Ni50Fe50_3h@80C catalyst powder was removed from NF surface using sonication and then deposited on Si wafer. (f) EDS spectrum of the sample.

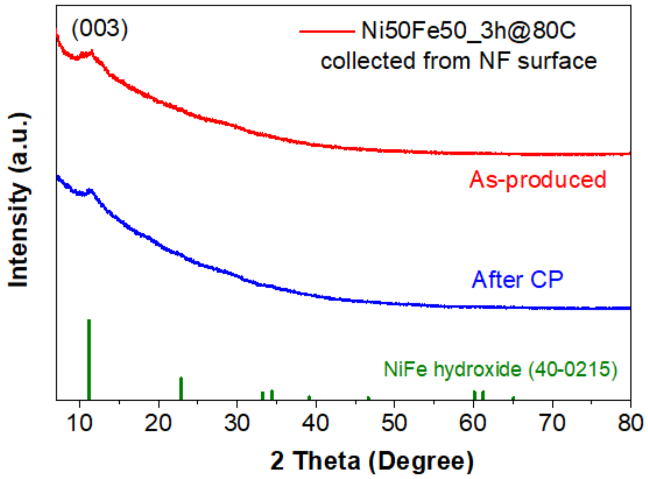

Supplementary Fig. 19. XRD pattern of Ni50Fe50_3h@80C catalyst powder removed from NF surface using sonication before and after CP test at 200 mA/cm^2 for 200 h. The broad peak at around 11.5° (associated to NiFe hydroxide) indicates the poor crystallinity of the sample.

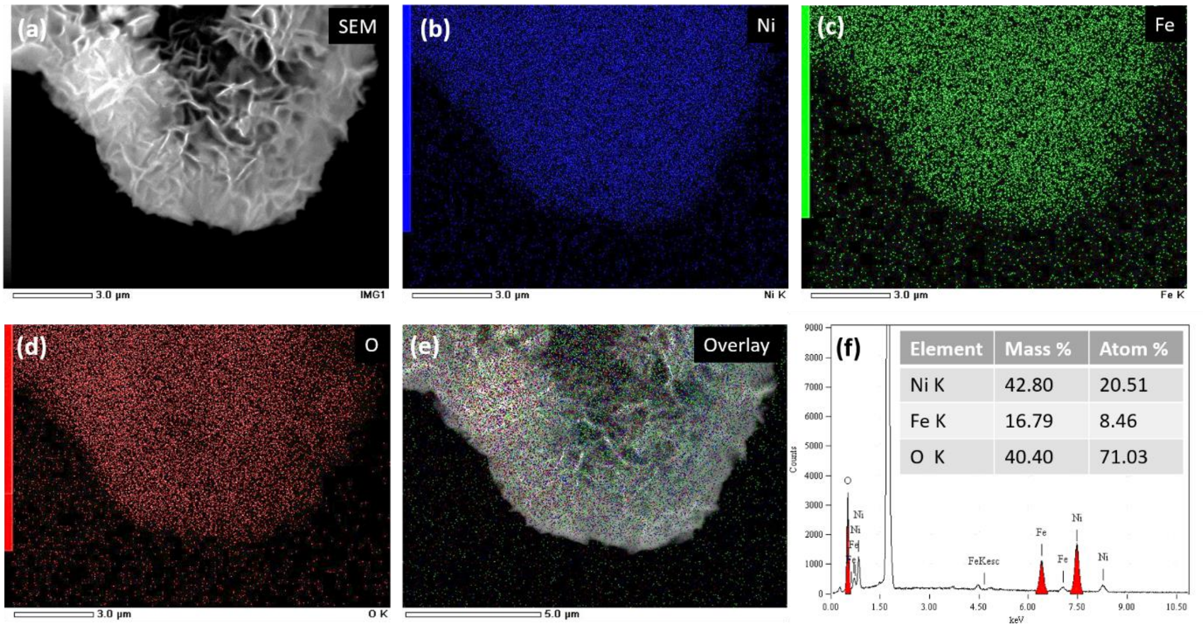

Supplementary Fig. 20. Determination of element distribution of tested Ni50Fe50_3h@80C catalyst powder collected from NF surface. (a) SEM images and the corresponding EDS maps for (b) Ni, (c) Fe, (d) O and (e) overlay. The tested Ni50Fe50_3h@80C catalyst powder was collected from NF surface after CP test at 200 mA/cm^2 for 200 h. (f) EDS spectrum of the sample.

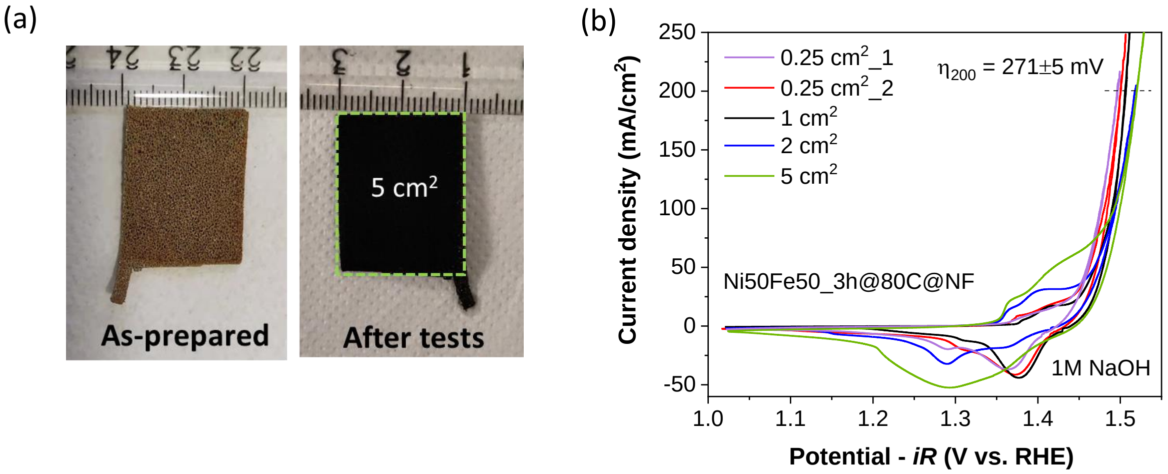

Supplementary Fig. 21. (a) Photographs of a representative 5 cm^2 NiFe@NF anode before and after its electrochemical characterization for the OER. (b) CV curves with iR-correction measured for NiFe@NF anodes with different (geometric) areas and produced from different batches.

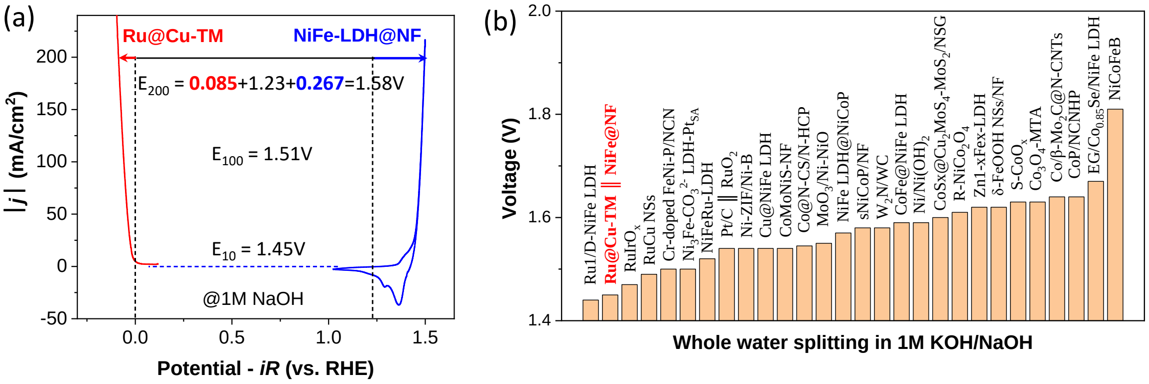

Supplementary Fig. 22. Whole water splitting. (a) Combination of Ru@Cu-TM cathode and NiFe@NF anode for whole water splitting; (b) Comparison between the performance of anode/cathode pairs (named as cathode || anode) for whole water splitting in 1M KOH/NaOH working at 10 mA/cm^2 (data taken from refs. shown in **Supplementary Table 5**). When anode and cathode are equal (bifunctional catalysts), only the name of the bifunctional electrode is reported.

Although Ru$_1$/D-NiFe LDH || Ru$_1$/D-NiFe LDH electrodes reported by Hou group in *Nat. Commun*. 12, 4587 (2021) demonstrates water splitting performance better those of our Ru@Cu-TM || NiFe@NF at 10 mA/cm^2 (at which ohmic losses in corresponding zero-gap AEL configurations should be considered negligible), the details regarding their cell configuration are not reported, making impossible a fair comparison with our technology.

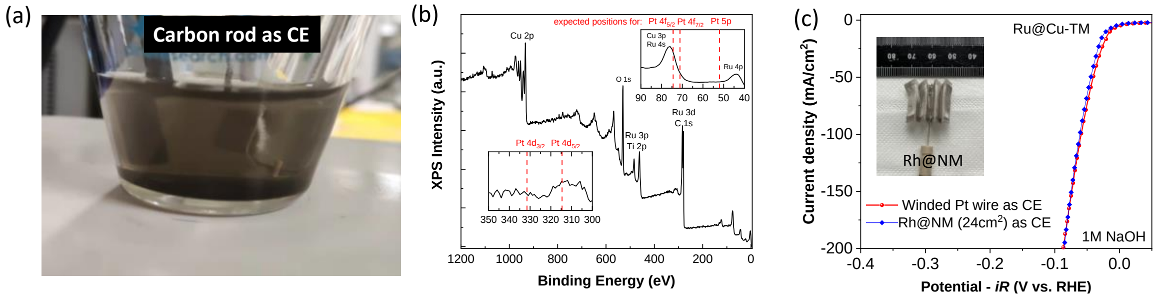

Supplementary Fig. 23. Investigation on counter electrode. (a) Photographs of three-electrode cell configurations based on carbon rod as the counter electrode (CE) after CP test for 5 h under -200 mA/cm^2. (b) Pt 4d, Pt 4f and Pt 5p XPS spectra acquired on the as-prepared Ru@Cu-TM using Pt wire as CE. No traces of Pt were found. (c) LSV curves of the electrodes synthesized using winded Pt wire and Ni mesh with thin Rh layer deposition (Rh@NM, 24 cm^2) as counter electrode. The inset image shows the photo of Rh@NM counter electrode.

Although carbon rod is a recommended CE for the HER, it is not stable due to its low oxidation potential being 0.207 V vs. RHE. Consequently, its oxidation becomes severe and causes the release of carbon ash under high working current (**Supplementary Fig. 23a**). Hence, we are keen to use winded Pt wire as CE, while the absence of XPS peaks at the positions, marked in the **Supplementary Fig. 23b,** in Pt 4d, Pt 4f and Pt 5p spectra acquired on the as-produced Ru@Cu-TM excluded the presence of Pt.

Besides, we later replaced the winded Pt wire by Rh deposited Ni mesh (Rh@NM) with a much bigger geometric size of 24 cm^2 (see the inset photo in **Supplementary Fig. 23c)**. On the one hand, the Rh provides a more robust stability than Pt in terms of corrosion. On the other hand, the much larger CE surface (compared to WE) would avoid/minimize the corrosion of CE and could therefore become appropriate for HER study in alkaline.[15] As can be seen, the produced electrodes using winded Pt wire and Rh@NM (24 cm^2) demonstrate similar activity towards alkaline HER, supporting that the use of Pt wire is not the reason for the good performance of our produced electrode.

In short, we acknowledge that the use of Pt is not a perfect choice, and the selection of an appropriate CE for HER research under high operating current conditions remains an unsolved problem.[16]

Supplementary investigations of AELs

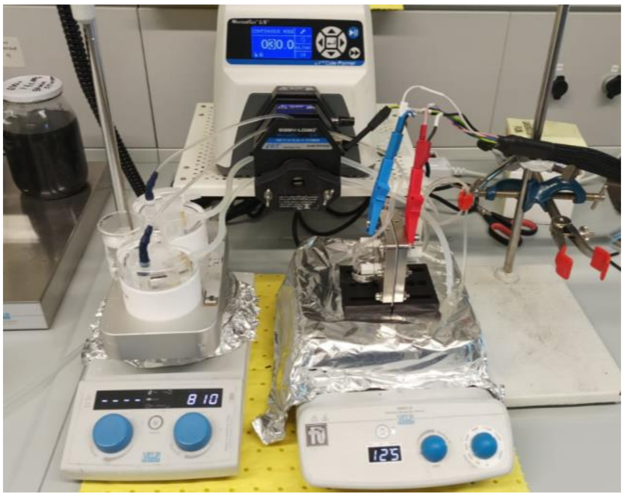

Supplementary Fig. 24. Photograph of the set-up used in this work to test AELs. During the test, a peristaltic pump (Masterflex L/S Series) was used to continuously supply the anodic and cathodic half-cells with a 30 wt% KOH solution at a flow rate of 30 mL/min per cm^2 of electrode area. Two containers (PTFE type) containing the anolyte and catholyte were loaded in an aluminum block heated by a hot-plate, to control the temperature of electrolyte to be 80 °C (controlled with a proportional-integral-derivative controller). The test was carried out under atmospheric (1 bar) system pressure.

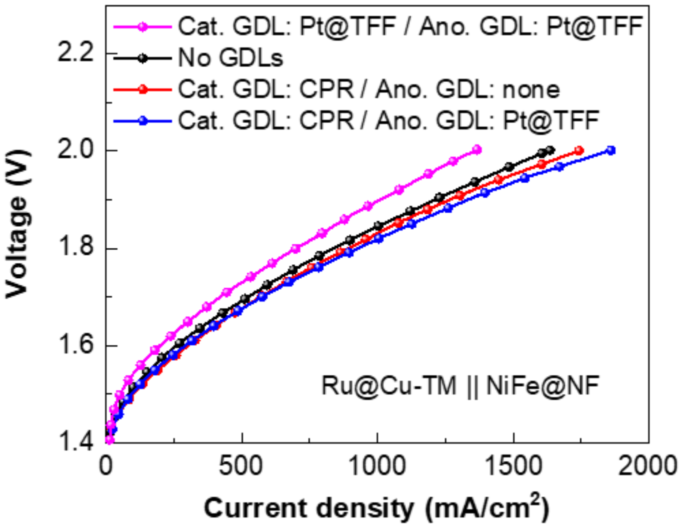

Supplementary Fig. 25. Effect of GDLs on the AEL performance: comparison between the polarization curves measured on zero-gap AELs using different GDL combination at cathode side (Cat.) and anode side (Ano.). Test conditions: Zirfon Perl UTP 500+ diaphragm; cathode: Ru@Cu-TM; anode: NiFe@NF; diaphragm: Zirfon Perl UTP 500+; electrolyte: 30 wt% KOH; temperature: 80 °C.

As shown in **Supplementary Fig. 25**, the addition of extra GDLs (e.g., carbon paper -CPR- GDL at the cathode side and platinized Ti fiber felt -Pt@TFF- GDL at the anode sides) increases the AEL performance (1 A/cm^2 at 1.82 V; 1.86 A/cm^2 at 2 V) compared with GDL-free AEL (e.g., 1 A/cm^2 at 1.84 V; 1.63 A/cm^2 at 2 V). Notably, CPR GDL was not evaluated at the anode side because of the corrosion of carbonaceous materials at potential higher than 1.23 V vs. RHE necessary for the OER (equilibrium potential of carbon = 0.207 V vs. RHE).[17] Meanwhile, Pt@TFF was initially considered

as performance-ideal GDL candidate because the Pt coating can prevent the oxidation of Ti during OER conditions, ensuring optimal electrocatalysts/GDL/bipolar plate electrical contact for long-term AEL performance. Indeed, CPR GDL at the cathode side and Pt@TFF at the anode side lead to a slight increase of the AEL performance (e.g., 1 A/cm^2 at 1.82 V; 1.86 A/cm^2 at 2 V) compared with only CPR GDL at the cathode side (e.g., 1 A/cm^2 at 1.83 V; 1.74 A/cm^2 at 2 V). Noteworthy, Pt@TFF at the cathode side deteriorates the GDL-free AEL performance. Similar effects have been previously observed for Ti fiber felts GDL in PEM AELs.[18] Despite the further addition of Pt@TFF GDL at anode could slightly increase its performance, its high cost, due to the presence of Pt (as well as Ti), motivated us to remove it for the subsequent tests.

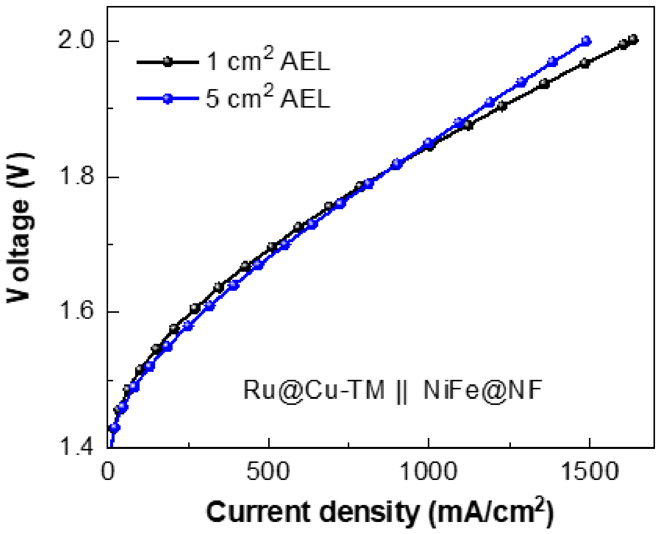

Supplementary Fig. 26. Polarization curve of zero-gap Ru@Cu-TM || NiFe@NF AELs with different electrode geometric areas, 1 and 5 cm^2. Test conditions: anode: NiFe@NF; diaphragm: Zirfon Perl UTP 500+; GDLs: CPR at Cat. and none at Ano.; electrolyte: 30 wt% KOH; temperature: 80 °C.

As can be observed in **Supplementary Fig. 26**, the AEL performances were approximately preserved with upscaling the electrode active area from 1 cm^2 to 5 cm^2, obtaining 0.5 A/cm^2 at 1.68 V, 1 A/cm^2 at 1.85 V.

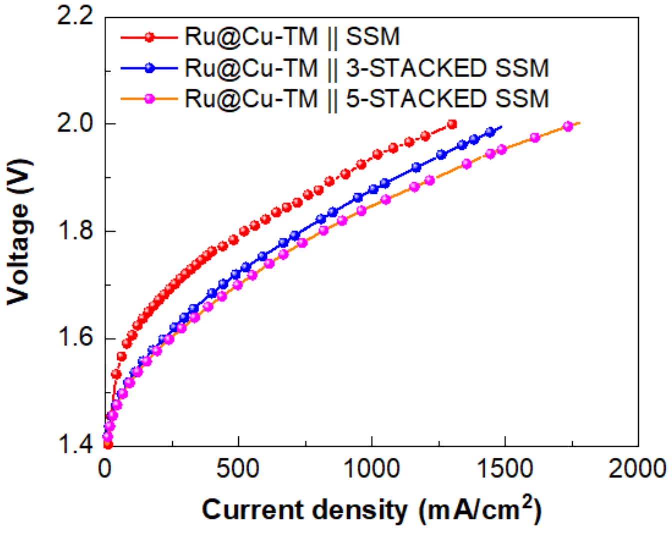

Supplementary Fig. 27. Comparison between the polarization curves measured on zero-gap AELs based on Ru@Cu-TM cathode and single or stacked SSMs as the anode. Test conditions: Zirfon Perl UTP 500+ diaphragm; GDLs: CPR GDL at Cat. and none at Ano.; electrolyte: 30 wt% KOH; temperature: 80 °C.

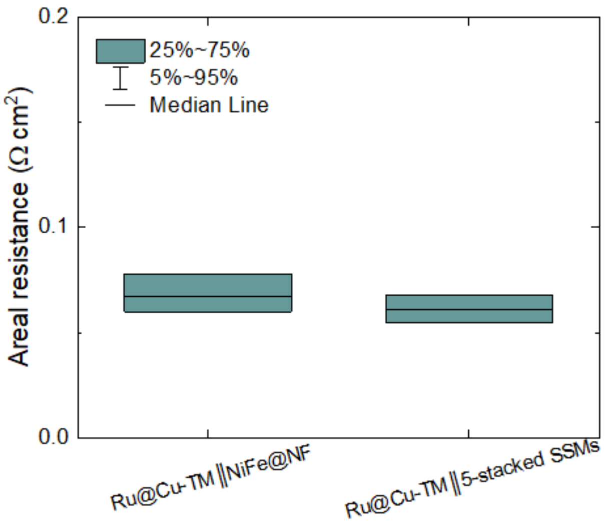

Supplementary Fig. 28. Statistic distribution of HFR recorded for two different AELs, i.e., Ru@Cu-TM || NiFe@NF and Ru@Cu-TM || 5-stacked SSMs. The resistance data were obtained by means of EIS measurements performed at the open circuit potential. The resistance data were then determined from the intercept of the real axis of the Nyquist plot at high frequencies.

The high-frequency-resistance (HFR) recorded on the Ru@Cu-TM cathode, thinner Zirfon Perl UTP220, but with different anodes (namely, NiFe@NF and 5-stacked SSMs) demonstrated a very similar ohmic resistance (average HFR: 0.067 Ω cm^2 vs. 0.061 Ω cm^2).

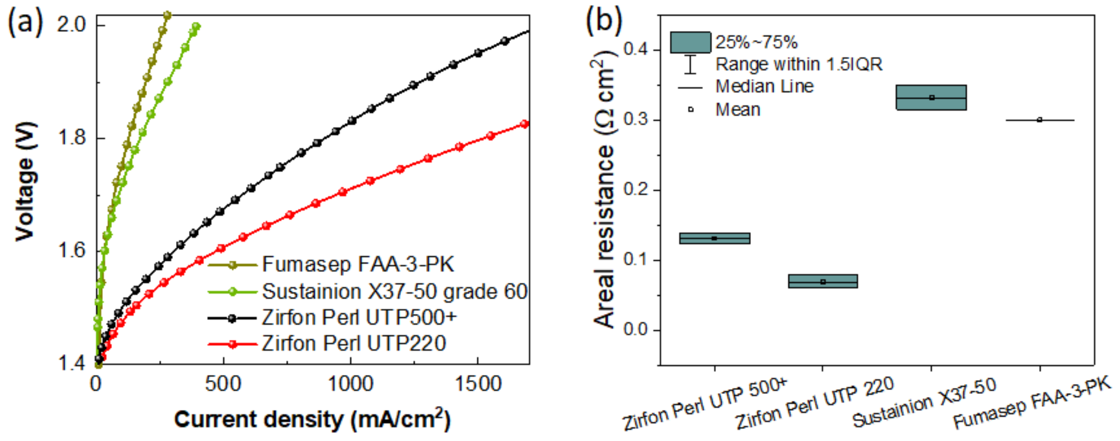

Supplementary Fig. 29. Effect of the separator on the EL performance: (a) comparison between the polarization curves measured for zero-gap AEM-ELs/AELs using different separators (AEMs or diaphragm). Test conditions: Zirfon Perl UTP 220 diaphragm; cathode: Ru@Cu-TM; anode: NiFe@NF; GDLs: CPR at Cat. and none at Ano.; electrolyte: 30 wt% KOH for AELs and 1 M KOH for AEM-ELs; temperature: 80 °C. (b) Statistic distribution of high-frequency-resistance (HFR) recorded on different separators. The diaphragm/membrane resistance was obtained by means of EIS measurements performed at the open circuit potential. The diaphragm/membrane resistance was determined from the intercept of the real axis of the Nyquist plot at high frequencies.

Along with our AELs, we assembled also two AEM-ELs using commercially viable AEMs, i.e., Sustainion X37-50 grade 60 and Fumasep FAA-3-PK. However, in the investigated electrolyte (1 M KOH) at which AEM commonly operates,[14] the assembled AEM-ELs performed significantly worse than our AELs based on Zirfon diaphragm (**Supplementary Fig. 29**). Noteworthy, for the case of Sustainion X37-50 grade 60 AEM, the assembly process typically resulted in damaging the AEMs, causing the short circuit of cells. To be able to evaluate the AEM-ELs, we specially reduced the electrode stack compression against the AEM, and this may have caused insufficient electrical contact between electrodes and bipolar plates.

Moving to the HFR recorded on different separators, the thinner Zirfon Perl UTP220 indeed demonstrated a much lower resistance (average HFR: 0.067 Ω cm^2), compared to either its thick counterpart of Zirfon Perl UTP500+ (average HFR: 0.130 Ω cm^2, similar as previously reported value[19]) or Fumasep FAA-3-PK (HFR: 0.300 Ω cm^2). Notably, the Sustainion X37-35 AEM displayed much higher resistance (average HFR: 0.330 Ω cm^2) than that of Zirfon diaphragm. We note that special precaution was taken at handling Sustainion X37-35 membranes, since the sharp features of NiFe-NF often led to the break of this AEM. Therefore, we have increased the thickness of the spacers, which however could have led to an insufficient compression of the cell stack, causing large resistances.

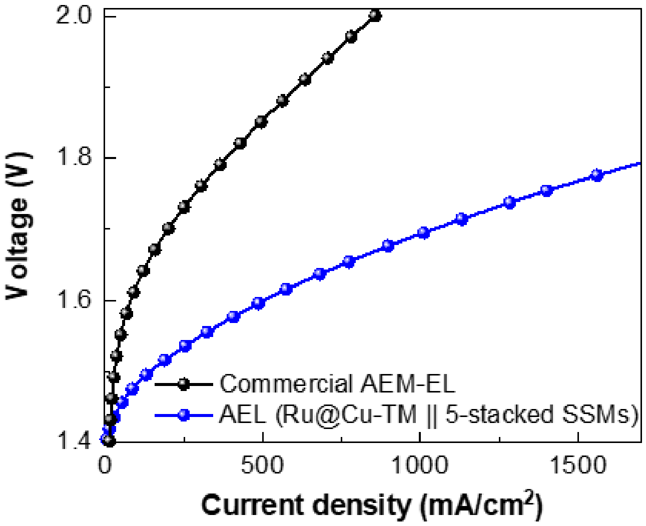

Supplementary Fig. 30. Comparison between the polarization curves measured for zero-gap commercial AEM-EL and our AEL. Test conditions for AEM-EL: AEM: Sustainion® 37-50; cathode: CPR GDL/ NiFeCo, anode: stainless steel fiber cloth GDL/$NiFe_2O_4$. Test conditions for our AEL: Zirfon Perl UTP 220 diaphragm, cathode: Ru@Cu-TM; anode: 5-stacked SSMs; CPR at Cat. and none at Ano. The ELs were tested with 30 wt% KOH at 80 ^oC.

A commercially available dual-feed AEM-EL (Dioxide Materials, NiFeCo || $NiFe_2O_4$, Sustainion® AEM, 5 cm^2) was also investigated to benchmark our AELs. The performance of the commercial AEM-ELs in 1 M KOH (1 A/cm^2 at 1.9 V) has been reported in previous works,[14] and is inferior to those of our AEL (1 A/cm^2 at 1.69 V). In our operating conditions, our AEL outperformed the AEM-EL (**Supplementary Fig. 30**), which, at 1.85 V, operated with only 0.5 A/cm^2. However, it should be mentioned that the AEM used by the AEM-EL may not be chemically stable in 30 wt% KOH at 80 °C.

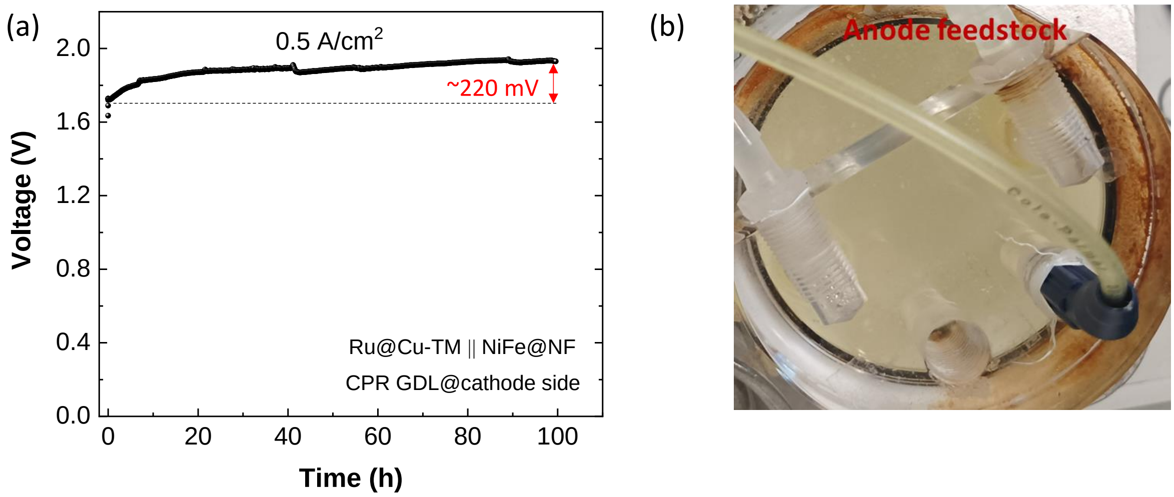

Supplementary Fig. 31. Continuous stability test for Ru@Cu-TM || 5-stacked SSMs AEL. (a) Stability measured for Ru@Cu-TM || NiFe@NF AELs using Zirfon Perl UTP 500+ diaphragm over 100 h of continuous operation at 0.5 A/cm^2. (b) Photograph showing the brown residuals associated to Fe species in the anode feedstock. Test conditions: CPR GDL at Cat. and none at Ano.; electrolyte: 30 wt% KOH; temperature: 80 °C.

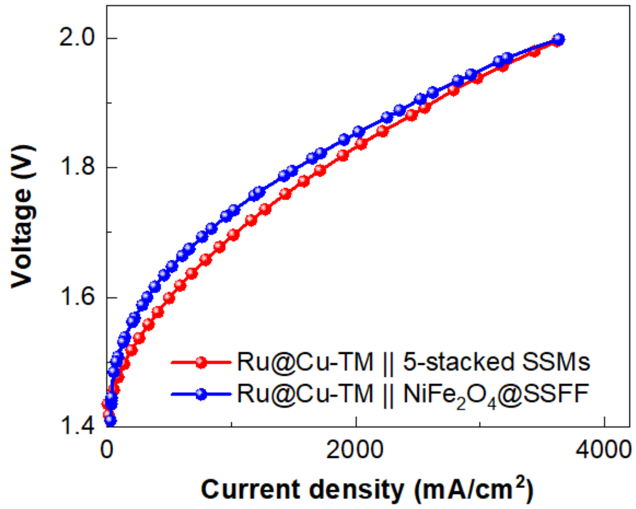

Supplementary Fig. 32. Polarization curves measured for zero-gap AELs based on Ru@Cu-TM cathode and using 5-stacked SSMs or commercial $NiFe_2O_4$@SSFF anodes. Test conditions: Zirfon Perl UTP 220 diaphragm; CPR GDL at Cat. and none at Ano.; electrolyte: 30 wt% KOH; temperature: 80 °C.

At 2.0 V, the AEL based on $NiFe_2O_4$@SSFF anode reached the same current density (3.6 A/cm^2) of our optimized AELs based on 5-stacked SSMs anode. Nevertheless, it displayed lower performance at lower cell voltage (i.e., 0.8 A/cm^2 vs. 1 A/cm^2 at 1.69 V).

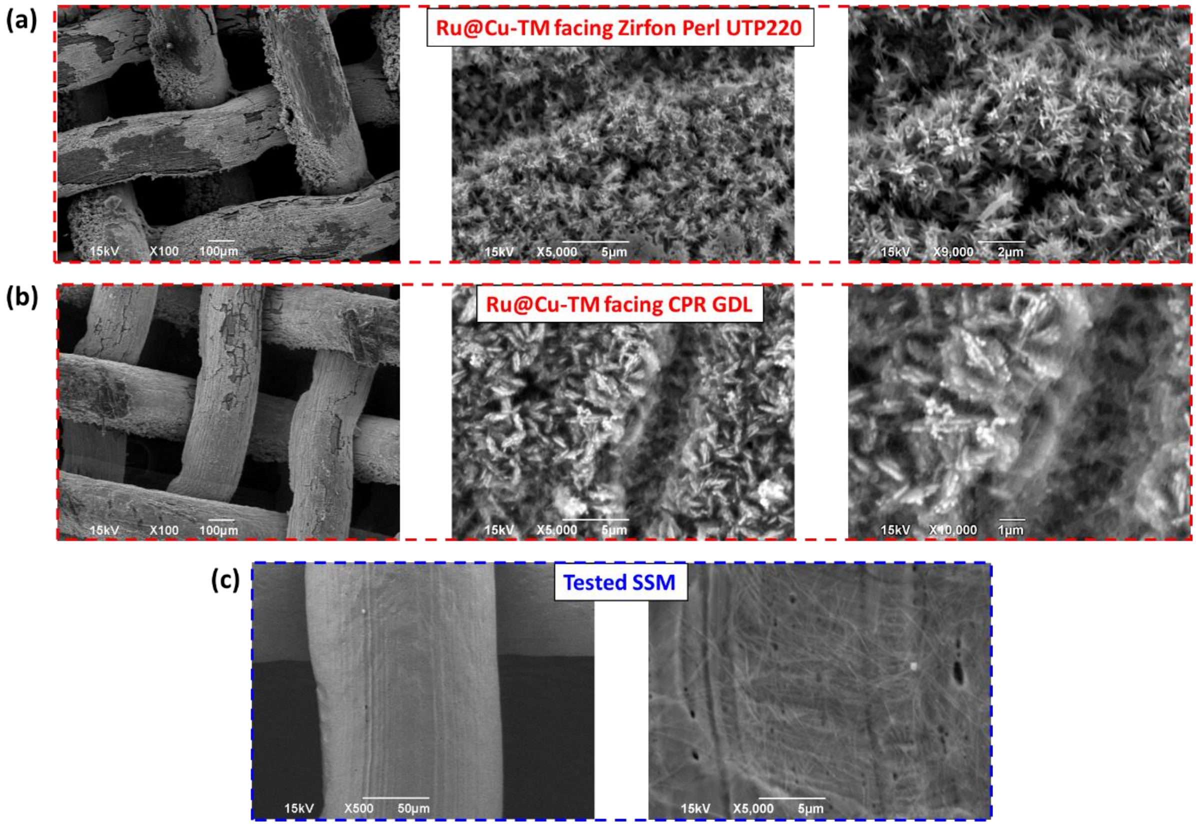

Supplementary Fig. 33. Electrodes morphology characterization after the AST for 24 h of the corresponding AEL (Ru@Cu-TM || 5-stacked SSMs): SEM images, at increasing magnification moving from left to right,

showing the Ru@Cu-TM surface facing the (a) Zirfon Perl UTP220 diaphragm and (b) the CPR GDL. (c) SEM images, at increasing magnification moving from left to right, of a SSM of the anode of tested AEL.

As shown in **Supplementary Fig. 33a**, after the AST of the Ru@Cu-TM || 5-stacked SSMs AEL for 24 h, the surface of the Ru@Cu-TM cathode facing Zirfon Perl UTP220 diaphragm displayed morphology made of Cu NWs. Instead, the surface of the Ru@Cu-TM cathode facing CPR GDL exhibited an intermediate morphology made of Cu NWs mixed with Cu NPLs (**Supplementary Fig. 33b**). After the AST, the SSMs of our AEL anode did not show any relevant morphological change compared to fresh SSMs. Additional morphological and chemical analyses of anode SSMs after long-term (1000 h) AEL operation at 1 A/cm^2 is reported later in **Supplementary Fig. 41**.

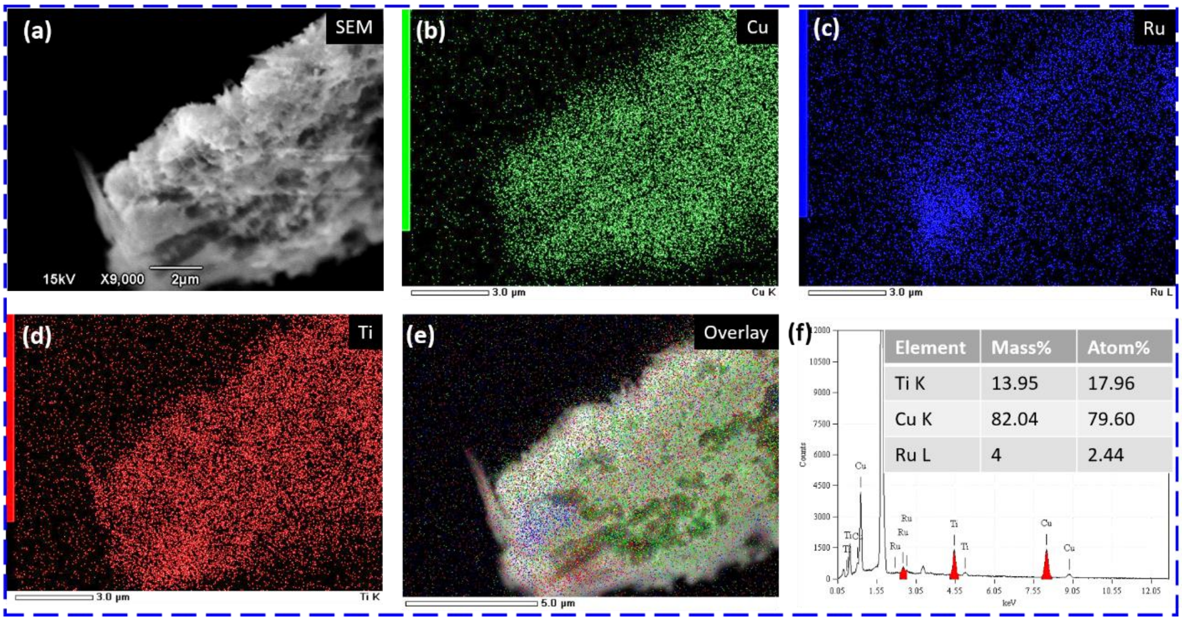

Supplementary Fig. 34. SEM-EDS characterization of the catalyst powder collected from the substrate of Ru@Cu-TM cathode and deposited on Si wafer, after 24 h-AST of the corresponding AEL (Ru@Cu-TM || 5-stacked SSMs): (a) SEM images and the corresponding EDS maps for (b) Cu, (c) Ru, (d) Ti and (e) overlay. (f) EDS spectrum of the sample.

The EDS maps acquired on the Ru@Cu-TM catalyst collected from the electrode substrate after AST procedure indicated a Ru content (2.97 atomic percentage -at%-, based on total amount of Ru and Cu) (**Supplementary Fig. 34**), which is still comparable to the fresh sample (2.25 at%) (**Supplementary Fig. 6**). As previously discussed, the formation of soluble CuOH species in harsh alkaline media and their subsequent redeposition under negative potential (-0.2V vs. RHE in our case), causing the transformation of Cu NPLs into Cu NWs, as well as the dissolution/redeposition of Ti species, may explain the slight change of EDS-detected Ru at% of the cathode surface.

(a)

(b)

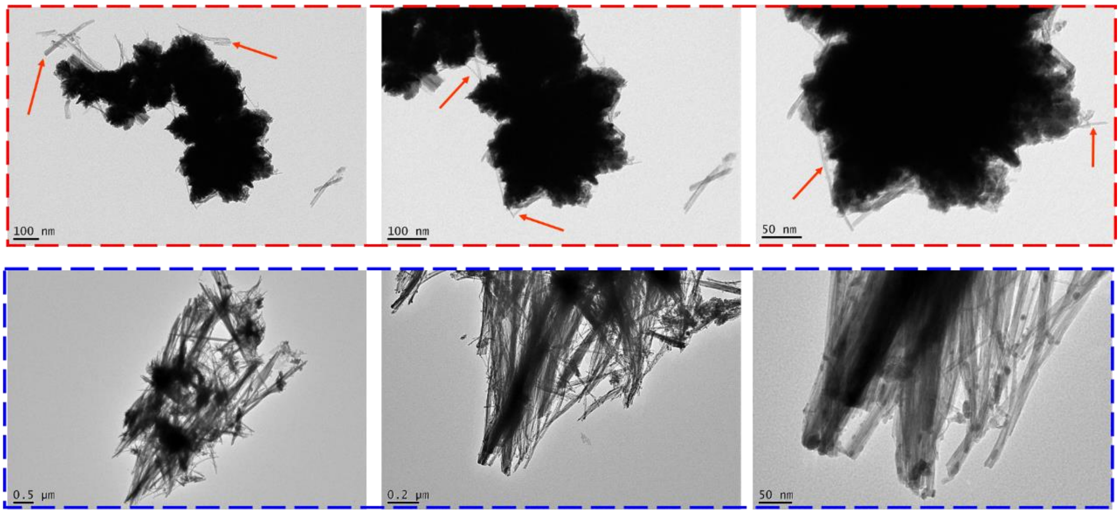

Supplementary Fig. 35. TEM characterization of the catalyst powder collected from TM substrate of Ru@Cu-TM cathode and deposited onto a carbon-coated Cu grid after the 24 h-ASTof the corresponding AEL (Ru@Cu-TM || 5-stacked SSMs). (a) TEM image showing a sample of the collected catalysts powder mainly made of NPLs, together with the occasional presence of NWs (indicated by red arrows) (b) TEM image showing a sample of the collected catalyst powder mainly made of nanowires.

As shown in **Supplementary Fig. 35**, the TEM images of the catalyst powder collected from Ru@Cu-TM cathode after the 24 h-AST indicated the presence of two different morphologies. The morphology shown in **Supplementary Fig. 35a** is mainly made of NPLs, together with the occasional presence of NW (previously recognized as NWs from SEM imaging), as observed on the surface of the cathode facing the CPR GDL (see SEM analysis of **Supplementary Fig. 33b**). The morphology of NPLs is similar to that observed for NPLs in the fresh cathode (see **Supplementary Fig. 5**). **Supplementary Fig. 35b**, instead, shows a TEM image evidencing the main presence of NWs, as observed on the surface of the cathode facing the Zirfon Perl UTP220 diaphragm (see **Supplementary Fig. 33a**).

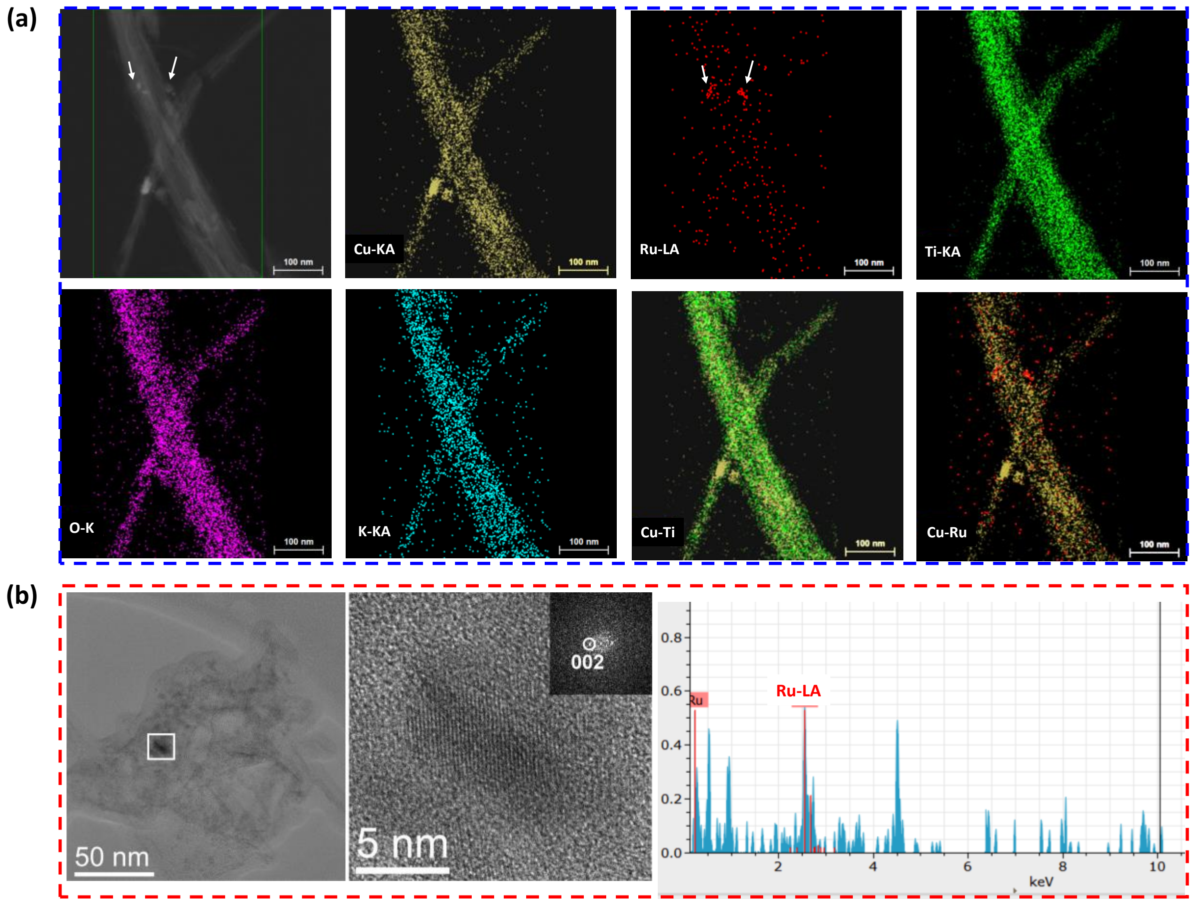

Supplementary Fig. 36. STEM-EDS chemical composition maps coupled with HAADF-STEM micrograph of Ru@Cu-TM catalyst after the 24 h-AST. (a) HAADF-STEM image, corresponding EDS maps for Cu, Ru, Ti, O, K and the overlapped maps for Cu-Ti, Cu-Ru are given. White arrows indicate the Ru NPs. (b) HRTEM micrographs of a Ru NP. The right micrograph shows the detail of the squared region in the left one, including a single-crystal Ru NP, as evidenced by the corresponding FFT. The corresponding STEM-EDS spectrum is also reported.

As shown in **Supplementary Fig. 36a**, the HAADF-STEM and EDS-mapping analysis of Ru@Cu-TM catalyst after the AST indicated that Cu, Ru, Ti, O and K are present along the nanowire structure, while Ru NPs is still present atop the NW, without aggregating. As explained in **Supplementary Fig. 34**, the initial Cu NPLs could transform into Cu NWs due to formation of soluble CuOH species in alkaline media and their subsequent redeposition under negative working potentials. Meanwhile, the etching of Ti substrate and redeposition of dissolved Ti ions onto Cu can also take place, leading to the growth of TiO_2 on the Cu NWs. Indeed, from the EDS data, the Ti amount increase after the AST compared to the fresh sample. The significant amount of K along with the NW structure, is attributed to the K contamination resulting from the AEL electrolyte (30 wt% KOH). **Supplementary Fig. 36b** shows the HRTEM images of a Ru NP, together with the corresponding STEM-EDS spectrum, evidencing that Ru NPs remain incorporated into the catalysts during AEL operation, without obvious morphological changes (**Supplementary Fig. 7**).

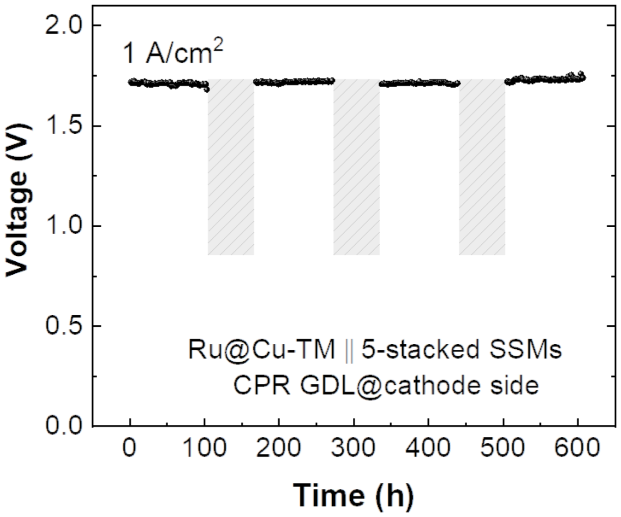

Supplementary Fig. 37. Stability test of Ru@Cu-TM || 5-stacked SSMs AEL using diaphragm of Zirfon Perl UTP 220, operating in quasi-continuous mode at 1 A/cm^2 for 608 h. The stripe part with gray shading indicates the shut-off of the AELs, kept at an operating current density of 0.05 A/cm^2, during weekend days.

The optimized AEL stably operated for about four weeks (608 h) at 1.0 A/cm^2 and 80 °C (shutting down the system at 0.05 A/cm^2 at room temperature during the weekend (quasi-continuous operation mode).

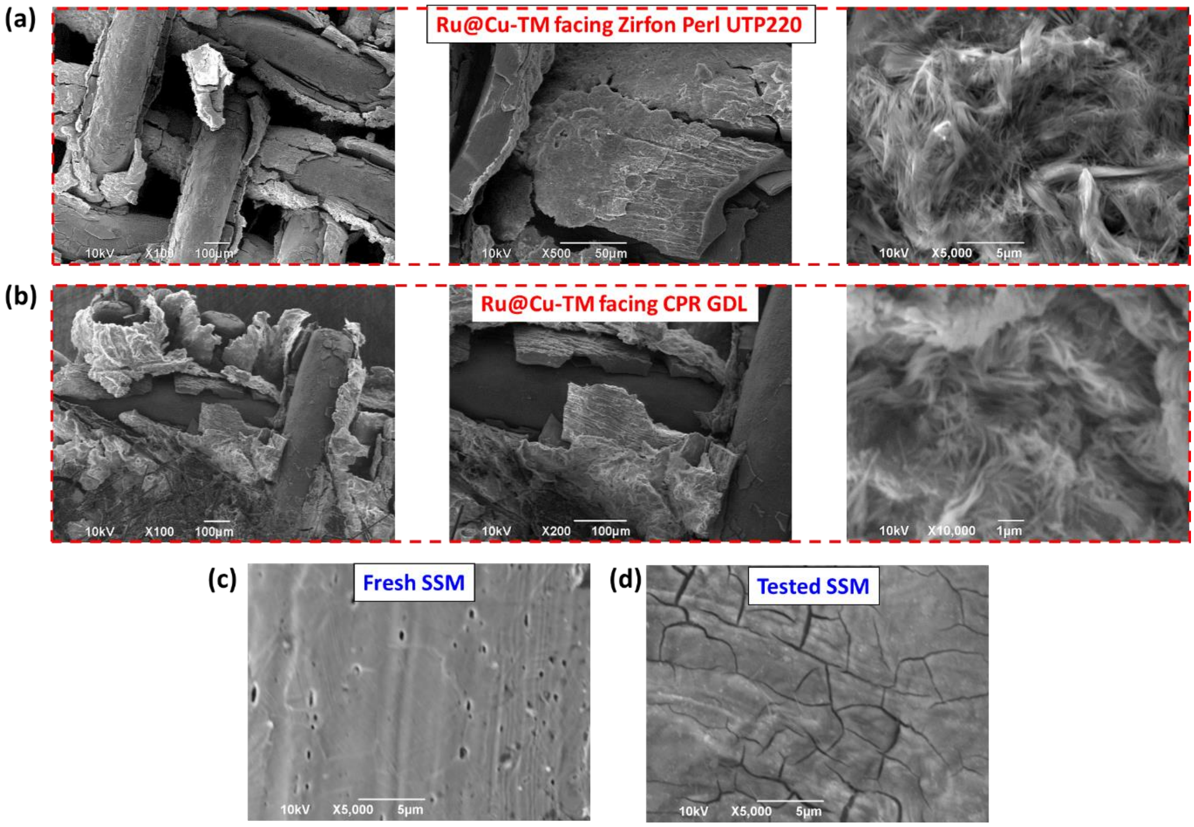

Supplementary Fig. 38. Electrodes morphology characterization after the quasi-continuous stability test for 608 h of the corresponding AEL (Ru@Cu-TM || 5-stacked SSMs): SEM images, at increasing magnification moving from left to right, showing the Ru@Cu-TM surface facing the (a) Zirfon Perl UTP220 diaphragm and (b) the CPR GDL. (c) SEM image of a fresh SSM, compared to (d) SEM image of a SSM of the anode of tested AEL. In some

part of the cathode, the peel-off of catalyst layer has been caused by its mechanical breakage during the disassembly operation of the AEL.

Bundle-like Cu NWs were observed on Ru@Cu-TM after 608 h quasi-continuous stability operation (**Supplementary Fig. 38a,b**). In the manuscript, we ascribed such morphological transformation to the *in-situ* dynamical nanostructuring of the Cu NPLs during operation at large current densities in harsh alkaline media. Indeed, the presence of CuOH species on Cu electrode has been evidenced up to -1.3 V vs. SCE (*ca.* -0.3 V vs. RHE) in 0.5 M NaOH, while no formation of oxidized Cu was found at -1.4 V vs. SCE.[20] Also, Cu adatoms can undergo oxidation to form CuOH even under more negative potentials in 1 M NaOH.[21] Thus, the change of the Ru@Cu-TM morphology can be associated to a complex kinetic competition between the oxidation of metallic Cu under alkaline conditions and the reduction of Cu species under the application of a cathodic potential.

Regarding the anode, the SSMs composing our anode (i.e., 5-stacked SSMs) exhibited some surface cracks after 608 h of quasi-continuous operation (**Supplementary Fig. 38c,d**). Such cracks can be ascribed to the oxidation/corrosion phenomena leading to the progressive formation of active FeO, Fe_2O_3 and NiO, which act as the active species for the OER.[22,23]

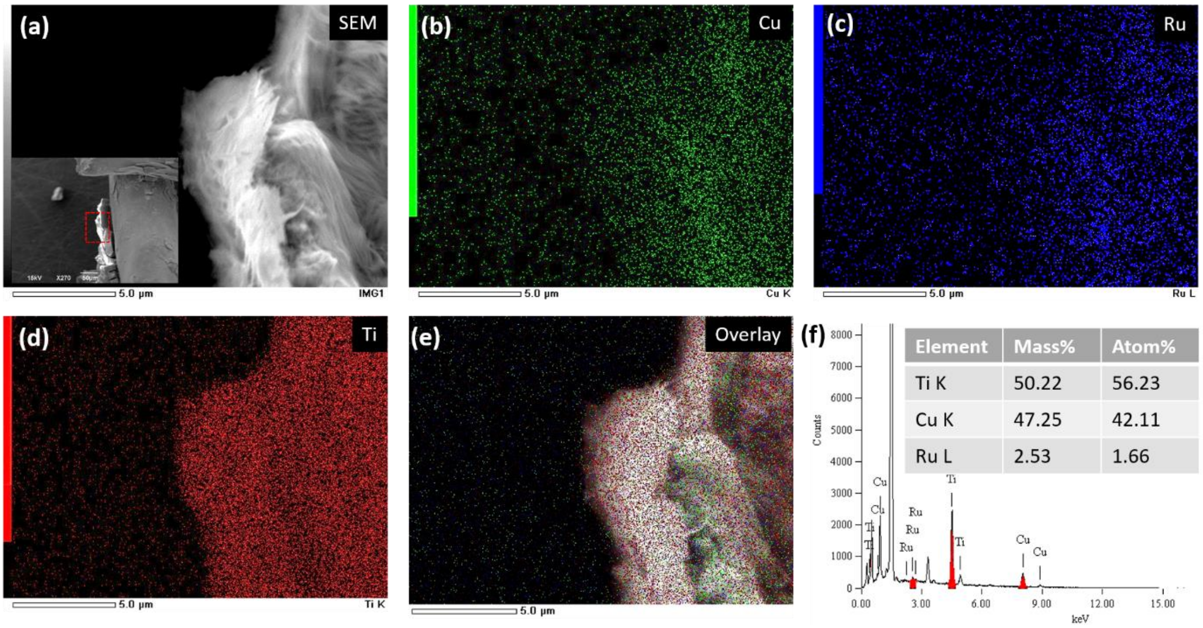

Supplementary Fig. 39. SEM-EDS characterization of Ru@Cu-TM cathode after the quasi-continuous stability test for 608 h of the corresponding AEL (Ru@Cu-TM || 5-stacked SSMs): (a) SEM image of the Ru@Cu-TM surface facing Zirfon Perl UTP220 diaphragm, and the corresponding EDS maps for (b) Cu, (c) Ru, (d) Ti and (e) overlay. (f) EDS spectrum of the sample.

The EDS maps shown in **Supplementary Fig. 39** acquired on the Ru@Cu-TM surface still evidenced the presence of Ru and Cu, together with increased Ti amount compared to fresh sample (**Supplementary Fig 6**), which could be ascribed to the dissolution (i.e., TM etching) and redisposition of Ti species on the surface of Cu NWs in harsh alkaline media (also see in **Supplementary Fig. 40**).

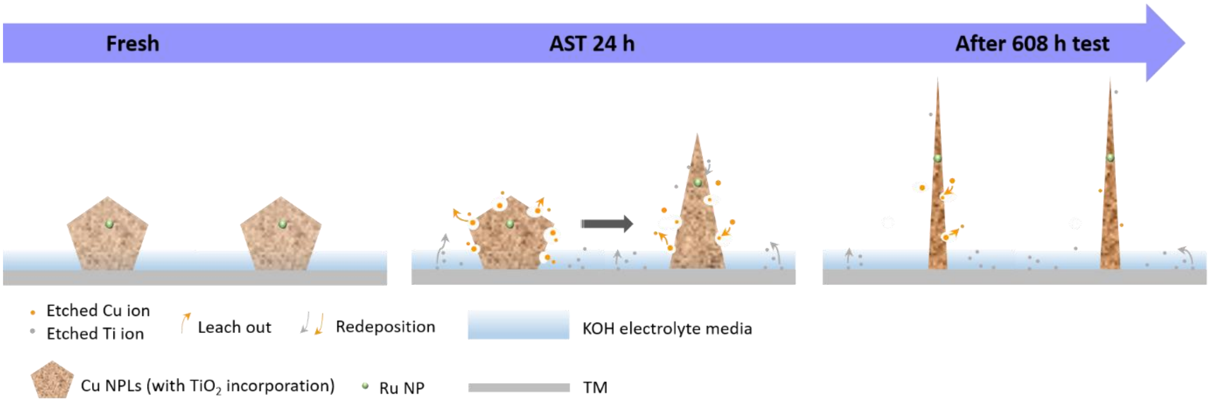

Supplementary Fig. 40. Proposed morphology evolution of Ru@Cu-TM cathode during AEL operation.

As shown in **Supplementary Fig. 40** and discussed in the manuscript, the Cu NPLs gradually change to Cu NWs due to formation of soluble CuOH species in alkaline media and their subsequent redeposition under negative working potentials, during which dissolved Ti ions (e.g., $HTiO_3^-$) originated by the etching of TM can redeposit onto Cu NPL/NW surface.

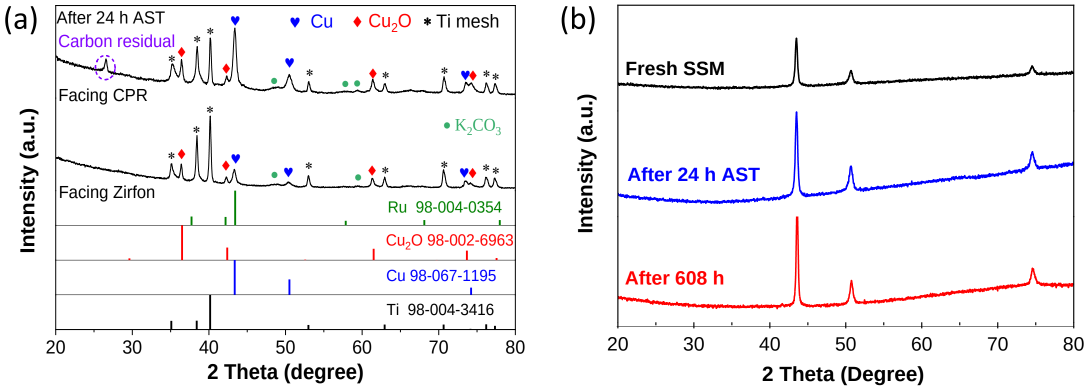

Supplementary Fig. 41. XRD patterens of (a) Ru@Cu-TM cathode side facing Zirfon Perl UTP220 diaphragm and carbon paper (CPR), respectively, after 24 h-AST. (b) XRD patterns of fresh SSM, and SSMs after AST and 608 h of quasi-continuous stability test of the corresponding AELs..

As shown in **Supplementary Fig. 41a**, the XRD pattern of Ru@Cu-TM cathode facing the CPR GDL showed intense peak for the phases of Cu and and Cu_2O after 24 h-AST, which is in accordance with the SEM results (see **Supplementary Fig. 33a,b)**, where the cathode surface facing CPR GDL retained the catalyst deposits after AEL disassembling. Contrary, detachment of catalyst layer from cathode surface and transfer to adhesive Zirfon Perl UTP220 diaphragm was observed for the cathode surface facing the diaphragm, resulting in less intense peaks ascribed to Cu species. The catalysts detachment from the cathode surface to the diaphragm was determined by the pressure applied to the (quasi) zero-gap AEL stack, as well as by the adhesivity of the Zirfon Perl UTP220, and does not represent an issue for the AEL operation.

The peak at 2θ of ~26.5° on cathode surface facing CPR GDL was ascribed to the carbonaceous residual of CPR. Some additional peaks could be assigned to K_2CO_3, which is formed by the reaction between residual electrolyte of KOH and CO_2 from air.

Lastly, no extra peaks were observed on the SSM anode after the stability tests of the corresponding AELs (24 h-AST and 608 h of quasi-continuous operation at 1 A/cm^2), compared to the fresh one (**Supplementary Fig. 41b**).

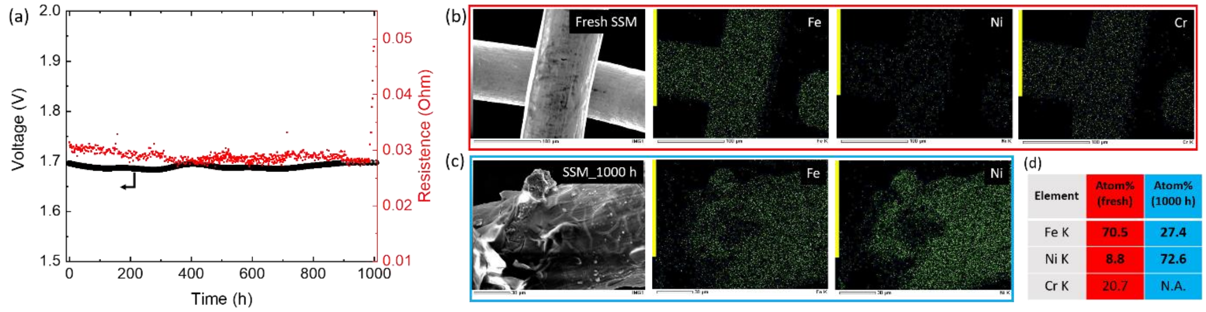

Supplementary Fig. 42. Stability measured for benchmark Pt/C-CPR || 5-stacked SSMs AEL using diaphragm of Zirfon Perl UTP 220. (a) Continuous operation at 1 A/cm^2 for 1000 h. SEM image and the corresponding EDS maps for (b) fresh, and (c)1000 h tested SSM. (d) EDS results of the sample (only the major elements are listed).

The Pt/C cathode was prepared through spray coating of inks of 20 wt% Pt/C in water:isopropanol (75:25), which were produced with a Pt/C concentration of 1 mg/mL and adding an amount ofa Nafion dispersion (10 wt%) corresponding to a 25 wt% Nafion content relatively to the solid content (i.e., Pt/C + Nafion). This cathode recipe was optimized in ref. [24]. The inks were sonicated in an Ultrasonic Bath USC–THD (WVR) for 1 h to get homogeneous dispersion. The so-produced inks were hand sprayed on CPR mounted on a hot plate pre-heated at 140°C, and the catalyst mass loading, i.e., m_{Pt}, was set to 150 μg/cm^2 by adjusting the amount of the sprayed ink.

As shown in **Supplementary Fig. 42a**, the benchmark Pt/C-CPR || 5-stacked SSMs AEL, based on the same configuration as Ru@Cu-TM || 5-stacked SSMs AEL, demonstrated a stable performance for 1000 h operation at 1 A/cm^2. However, we pointed out that after stability test, the composition of SSM surface changed compared to its initial state, thus progressively improving its catalytic activity towards the OER. In particular, Ni was progressively exposed at the SSM surface, suggesting the leaching of Fe and Cr (**Supplementary Figs. 42b-d)**. Thus, SSM represent a promising commercially available and cost-effective anode for high-performance AELs operating with 30 wt% KOH electrolyte at 80 °C, as also indicated by recent studies.[22,23]

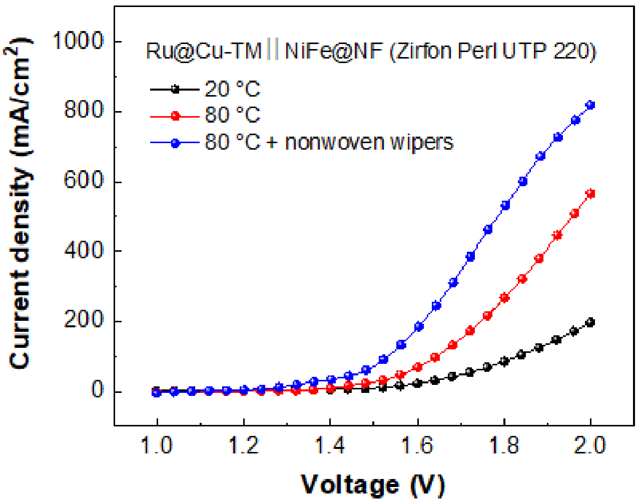

Supplementary Fig. 43. Polarization curves measured for capillarity-fed Ru@Cu-TM || NiFe@NF AELs at room temperature and at 80 °C, and the AELs including nonwoven wipers as additional spacers. Test conditions: CPR GDL at Cat. and none at Ano.; electrolyte: 30 wt% KOH.

The room temperature electrode-level characterization of the cathodes (see **Supplementary Figs. 2a,f** in the main text) intrinsically proves their potential for E-TAC systems. Indeed, the HER process is the same as in alkaline electrolysis, except that it occurs at ambient temperature instead of high temperature (e.g., 80 °C as for the case of the proposed AELs). Thus, the designed cathodes can represent advantageous alternatives to traditional cathodes, e.g., Raney-Ni[25] or NiMo electrodes and other HER electrocatalysts, e.g., platinized Ni-coated SSM[26]. Based on this rationale, a preliminary study on capillarity-fed AELs was also carried out using Ru@Cu-TM || NiFe@NF AEL configuration (**Supplementary Fig. 43**). At room temperature, the proposed capillarity-fed AELs reached ~0.20 A/cm^2 at 2.0 V. By increasing the temperature up to 80 °C, the cell reached 0.56 A/cm^2 at 2.0 V. Lastly, two pieces of nonwoven wipers were added as extra spacers, providing additional pathways for the capillarity-induced transport of the electrolyte beyond the one given by Zirfon Perl UTP 220 diaphragm. Thus, at 80 °C, the as-modified capillarity-fed AELs reached a current density as high as 0.82 A/cm^2 at 2.0 V.

Supplementary Table 1. Comparison between the HER activity of various PGM-based catalysts in 1 M KOH/NaOH reported in the past 5 years.

**Sample**	**Electrode binder**	**PGM loading**	**Tafel slope (mV/dec)**	**η10 (mV)** [a]	**η50 (mV)**	**η100 (mV)**	**η200 (mV)**	**η500 (mV)**	**Mass activity (A/mg)** [c]	**Reference**
**Ru@Cu-TM**	**Self-supporting**	**53 μg/cm^2 (ICP), ~1.8 wt%**	**32**	**17**	**44**	**59**	**85**	**144**	**4.87 @ 100 mV<br>2.58 @ 70 mV<br>0.28 @ 20 mV**	**This work**
Au-Ru NWs	Nafion	/	30.8	50	/	/	/	/	/	*Nat. Chem.* 10, 456–461 (2018)
Ru@C_2N	Nafion	~285 μg/cm^2	38	17	/	/	/	/	~0.035 @ 17 mV [d]	*Nat. Nanotech.* 12, 441–446 (2017)
Ru_1/D-NiFe LDH *	Self-supporting	1.2 wt% of Ru	29	18	/	61	/	/	14.65 @ 100mV	*Nat. Commun.* 12, 4587 (2021)
Pt_{SA}-NiO/Ni @Ag NWs*	Self-supporting	/	27.07	26	~60 [b]	85	/	/	20.6 @ 100 mV	*Nat. Commun.* 12, 3783 (2021)
Pt_{SA}-C_1N_1 *	Nafion	~2.5 wt% (ICP)	36.8	46	~120	~200	/	/	/	*Nat. Commun.* 11, 1029 (2020)
Ru@MWCNT	Nafion	/	27	17	/	/	/	/	0.186 @ 20 mV	*Nat. Commun.* 11, 1278 (2020)
Sr_2RuO_4	Nafion	/	61	51	~130	/	/	/	0.4 @ 100 mV	*Nat. Commun.* 10, 149 (2019)
Co-substituted Ru	Nafion	153 μg/cm^2	29	13	~30	/	/	/	~0.196 @ 30 mV [d]	*Nat. Commun.* 9, 4958 (2018)
Hexagonal Pt-Ni alloy	Nafion	11.5 at%, **~18.3 wt%**	74	65	/	/	/	/	/	*Nat. Commun.* 8, 15131 (2017)
RuCo@N-C	Nafion	3.58 wt%	31	28	~130	218	/	/	/	*Nat. Commun.* 8, 14969 (2017)
Pt_3Ni_2-NWs-S/C	Nafion	15 μg/cm^2	/	42	/	/	/	/	~0.667 @ 42 mV [d]	*Nat. Commun.* 8, 14580 (2017)
Ru/RuO_2	Nafion	/	35	17	~40	~70	/	/	/	*Energy Environ. Sci.* 14, 5433-5443 (2021)

Pt-Ni NTAs	Self-supporting	16 μg/cm^2	38	23	~45	71	/	/	4.27 @ 50 mV	*Energy Environ. Sci.* 14, 1594-1601 (2021)
Pt_{SA}–$Co(OH)_2$@Ag NW *	Self-supporting	~2.8 wt% determined by ICP	35.72	29	77	104	/	/	1.6 @ 100mV	*Energy Environ. Sci.* 13, 3082-3092 (2020)
Ni_5P_4-Ru	Nafion	/	52	54	/	/	/	/	/	*Adv. Mater.* 32, 1906972 (2020)
RuP (L-RP/C)	Nafion	21.4 wt%	34	18	~55	71	/	/	/	*Adv. Mater.* 30, 1800047 (2018)
Pt-Ni ASs	Nafion	17 μg/cm^2	27	27.7	~70	/	/	/	2.8 @ 70 mV	*Adv. Mater.* 30, 1801741 (2018)
$Nb_{0.23}RhO_x$/GDY	Self-supporting	0.3049 wt%	42	14	42	/	/	/	/	*Adv. Sci.* 9, 2104706 (2022)
NiRu-MOF/NF	Self-supporting	Ru/Ni = 5.4/94.6	90	51	~110	156	/	/	/	*ACS Appl. Mater. Interfaces* 12, 34728 (2020)
Pt/Ni_3S_2/NF	Self-supporting	53 μg/cm^2	73	10	/	~90	~120	207	5.52 @ 150 mV	*ACS Appl. Mater. Interfaces* 12, 39163 (2020)
Pt_{at}–CoP MNSs/CFC *	Self-supporting	5.89 μg/cm^2	30.28	~13	~30	/	/	/	3 @ 70 mV	*J. Mater. Chem. A* 8, 11246 - 11254 (2020)
Cu-Ru/Ti	Self-supporting	63 μg/cm^2	34	23	65	99	140	/	~1.59 @ 99 mV [d]	*J. Mater. Chem. A* 8, 10787-10795 (2020)
$PtNi_5$-0.3	Nafion	/	19.2	26.8	/	~90	/	/	2.36 @ 70 mV	*Adv. Funct. Mater.* 31, 2008298 (2021)
pAu_3Pt/NF	Self-supporting	/	42.3	/	40.1	~55	/	/	/	*Sustainable Energy Fuels* 4, 4878-4883 (2020)
Ru NCs/BNG	Nafion	17.17 wt% Ru	28.9	14	50	/	/	/	/	*Nano Energy* 68, 104301 (2020)
Ru-MoS_2/CC	Self-supporting	Ru in Ru-MoS_2: 0.37 wt%	114	41	~110	171	/	/	/	*Appl. Catal. B: Environ.* 249, 91-97 (2019)
RuNi-NSs@PANI	Self-supporting	7.2wt%	38.54	21.9	/	~160	/	/	/	*J. Catal.* 375, 249-256 (2019)

PtCo–Co/TiM	Self-supporting	/	35	28	~60	~100	/	/	/	*Nanoscale* 10, 12302-12307 (2018)
Pt–Co(OH)$_2$/CC	Self-supporting	5.7 wt %	70	32	~75	122	/	/	/	*ACS Catal.* 7, 7131 (2017)
RuP$_2$@NPC	Nafion	/	69	52	~110	/	/	/	/	*Angew. Chem. Int. Ed.* 56, 11559–11564 (2017)

Note: [a] Due to the possible current contribution from the substrate for *in-situ* fabricated electrodes, we suggest comparing their performances at current densities higher than 50 mA/cm^2 when reliable evaluation practices are not indicated. [b] The overpotential values with the symbol (~) are extracted from their corresponding LSV plots. [c] Mass activity is obtained by dividing the current density by the corresponding loading amount of PGM at a certain overpotential. [d] Calculated value.

Acronyms: NWs - nanowires, SA, at and * - single atom, NF - nickel foam, CNT - carbon nanotube, GDY – graphdiyne, CFC - carbon fiber cloth.

Supplementary Table 2. Free energies on hydrogen desorption on pure metals.

**Metal**	**ΔG_{H^*} (eV)**
Cu	0.23
Ru	0.62

3-layer slabs were adopted that measured 12.8 × 13.3 Å (Cu) and 13.6 × 14.1 Å (Ru). 3×3×1 gamma-centered k-points grids were adopted. All other settings and approximations are as described in the Methods section of the main text.

Supplementary Table 3. Water dissociation energies on Cu and Ru.

**Metal**	**ΔE_{diss}^{H2O} (eV)**
Cu	+0.004
Ru	-0.452

Computational settings as in Supplementary Table 2, except that the plane wave energy cutoff was set to 500 eV for the simulations presented in this table. The water dissociation energy was calculated as $\Delta E_{diss}^{H2O} = E_{slab}(H^*) + E_{slab}(OH^*) - E_{slab}(H_2O^*) - E_{slab}$, where E_{slab} (H*) indicates the energy of the metal slab with hydrogen adsorbed, and the same goes for OH* and H_2O*. E_{slab} is the energy of the clean metal slab. Note that the most stable adsorption sites were considered, namely hollow (fcc on Cu) for H and OH and atop for H_2O

Supplementary Table 4. Comparisons between the OER activity of Ni- and/or Fe-based catalysts in 1 M KOH/NaOH reported in the past 5 years

Sample	η10 (mV)	η50 (mV)	η100 (mV)	η200 (mV)	Reference
**NiFe@NF**	**207**	**232**	**247**	**267**	**This work**
NiMoFeO@NC	/	270	/	/	*Matter* 3, 2124-2137 (2020)
Ni(Fe)OOH–FeS_x	/	~300	/	/	*Nat. Commun.* 11, 5075 (2020)
NiFe-LDH@Fe foil	269	/	~340	/	*Nat. Commun. 9, 2609 (2018)*
a-LNF(t-d)	189	/	310	/	*Adv. Mater.* 31, 1900883 (2019)
NiTe/NiS	/	/	257	/	*Adv. Mater.* 31, 1900430 (2019)
$NiFe(OH)_x$/FeS	/	245	261	~280	*Adv. Funct. Mater.* 29, 1902180 (2019)
DR-Ni_3FeN/N-G	250	/	310	/	*Adv. Funct. Mater.* 28, 1706018 (2018)
Fe-NiMo-NH_3/H_2	192	/	219	/	*Adv. Energy Mater.* 39, 2002285 (2020)
Ni/$NiFeMoO_x$/NF	255	/	289	/	*Adv. Sci.* 7, 1902034 (2020)
FeOOH(Se)	/	/	310	/	*J. Am. Chem. Soc.* 141, 7005-7013 (2019)
F-NiFe-A	218	/	/	~270	*Nano Lett.* 21, 492−499 (2021)
NiFe-OH-F-SR	~190	/	228	/	*Nano Lett.* 19, 530–537 (2019)
NF-AC-NiOx-Fe	215	/	248	/	*ACS Cent. Sci.* 5, 558–568 (2019)
Ni:FeOOH	214	/	270	~300	*ACS Catal.* 9, 5025–5034 (2019)
a/c-NiFe−G/NF	217	~260	-	-	*ACS Catal. 11, 12284−12292 (2021)*
Fe-doped β-$Ni(OH)_2$	219	/	280	~300	*ACS Energy Lett.* 4, 622–628 (2019)
Ni-Fe LDH hollow prisms	280	/	370	/	*Angew. Chem. Int. Ed.* 172–176 (2018)
fcc-NiFe@NC	226	/	263	/	*Angew. Chem. Int. Ed.* 58, 6099–6103 (2019)
$Ni_{0.8}Fe_{0.2}$-AHNA	190	/	248	/	*Energy Environ. Sci.* 13, 86–95 (2020)
EG/$Co_{0.85}Se$/NiFe LDH	203	/	260	/	*Energy Environ. Sci.* 9, 478–483 (2016)
$Ni_{60}Fe_{30}Mn_{10}$	208	/	270	/	*Energy Environ. Sci.* 9, 540–549 (2016)
$Ni_{12}P_5$-Fe_2P-NbP	/	263	280	~310	*J. Mater. Chem. A* 9, 9918-9926 (2021)
HG-$NiFe_x$	310	/	380	/	*Sci. Adv.* 4, 7970 (2018)
Ni_2P@$FePO_xH_y$	220	/	260	/	*Appl. Catal. B: Environ. 306, 121127 (2022)*

Note: The overpotential values with the symbol (~) are extracted from their corresponding LSV plots. Due to the possible current contribution from the substrate and/or oxidation of catalysts, we suggest comparing their performances at current densities higher than 50 mA/cm^2 when reliable evaluation practices are not indicated.

Supplementary Table 5. Comparison between overall water splitting voltages at 10 mA/cm^2 measured for various catalysts in 1 M KOH/NaOH reported in the past 5 years.

**Catalysts**	**Cell voltage at 10 mA/cm^2 (V)**	**Reference**
NiCoFeB	1.81	*Small* 15, 1804212 (2019)
EG/$Co_{0.85}$Se/NiFe LDH	1.67	*Energy Environ. Sci.* 9, 478-483 (2016)
CoP/NCNHP	1.64	*J. Am. Chem. Soc.* 140, 2610 (2018)
Co/β-Mo_2C@N-CNTs	1.64	*Angew. Chem. Int. Ed.* 58, 4923 (2019)
Co_3O_4-MTA	1.63	*Angew. Chem. Int. Ed.* 56, 1324 (2017)
S-CoO_x	1.63	*Nano Energy* 71, 104652 (2020)
VOOH/NF	1.62	*Angew. Chem. Int. Ed.* 56, 573 (2017)
δ-FeOOH NSs/NF	1.62	*Adv. Mater.* 30, 1803144 (2018)
$Zn_{1-x}Fe_x$-LDH	1.62	*Small* 14, 1803638 (2018)
R-$NiCo_2O_4$	1.61	*J. Am. Chem. Soc.* 140, 13644 (2018)
CoS_x@Cu_2MoS_4-MoS_2/NSG	1.6	*Adv. Energy Mater.* 10, 1903289 (2020)
Ni/$Ni(OH)_2$	1.59	*Adv. Mater.* 32, 1906915 (2020)
CoFe@NiFe LDH	1.59	*Appl. Catal. B: Environ.* 253, 131 (2019)
W_2N/WC	1.58	*Adv. Mater. 32*, 1905679 (2020)
sNiCoP/NF	1.58	*Nano Lett.* 16, 7718-7725 (2016)
NiFe LDH@NiCoP	1.57	*Adv. Funct. Mater.* 28, 1706847 (2018)
Mo-Co_9S_8@C	1.56	*Adv. Energy Mater.* 10, 1903137 (2019)
MoO_3/Ni-NiO	1.55	*Adv. Mater.* 32, 2003414 (2020)
Co@N-CS/N-HCP	1.545	*Adv. Energy Mater.* 9, 1803918 (2019)
CoMoNiS-NF	1.54	*J. Am. Chem. Soc.* 141, 10417-10430 (2019)
Cu@NiFe LDH	1.54	*Energy Environ. Sci.* 10, 1820-1827 (2017)
Ni-ZIF/Ni-B	1.54	*Adv. Energy Mater.* 10, 1902714 (2019)
Pt/C ‖ RuO_2	1.54	*Adv. Mater.* 31, 1900178 (2019)
NiFeRu-LDH	1.52	*Adv. Mater.* 30, 1706279 (2018)
Cr-doped FeNi-P/NCN	1.5	*Adv. Mater.* 31, 1900178 (2019)
Ni_3Fe-CO_3^{2-} LDH-Pt_{SA}	1.5	*Energy Environ. Sci.* 14, 6428-6440 (2021)
RuCu NSs	1.49	*Angew. Chem. Int. Ed.* 58, 13983 (2019)
$RuIrO_x$	1.47	*Nat. Commun.* 10, 4875 (2019)
NiFe NPs	1.47	*Nat. Commun.* 10, 5599 (2019)
**Ru@Cu-TM ‖ NiFe@NF**	**1.45**	**This work**
Ru_1/D-NiFe LDH	1.44	*Nat. Commun.* 12, 4587 (2021)

Supplementary Table 6. Comparison between the water splitting performances of our AELs and those of ELs reported in the past 5 years.

Electrolyzer type	Temperature (°C), Pressure	Cathode (mg/cm^2)	Anode (mg/cm^2)	Diaphragm/Membrane	@0.5 A/cm^2 (V)	@1 A/cm^2 (V)	@2 A/cm^2 (V)	Current density at 2V (A/cm^2)	Stability tested	Reference
**AEL**	**80 (30 wt% KOH)**	**Ru@Cu-TM (Ru loading: 0.053)**	**5-stacked SS mesh**	**Zirfon Perl UPT 220**	**1.60**	**1.69**	**1.83**	**3.63**	**608 h (ON-OFF) at 1 A/cm^2, Stable at ~1.71 ± 0.01 V; 300 h (Continuous) at 1A/cm^2, Stable at ~1.74 V**	**This work**
			NiFe@NF	Zirfon Perl UPT 220	1.61	1.71	1.87	2.98	N.A	
			NiFe@NF	Zirfon Perl UPT 500	1.67	1.83	/	1.74	100 h at 0.5 A/cm^2 (~2.2 mV/h decay)	
AEL	80 (30 wt% KOH)	Ni plate	Ni plate	Zirfon Perl UPT 500	~2	/	/	0.5	N.A	*ACS Sustainable Chem. Eng.* 6, 4829−4837 (2018)
AEL	80 (30 wt% KOH)	Raney-Ni	NiFe-LDH	Zirfon Perl UPT 500	~1.75	~1.95	~2.4	~1.1	N.A	*Chem. Eng. J.* 428, 131149 (2022)
	80 (30 wt% KOH)	Raney-Ni	NiFe-LDH	80 wt% ZrO_2/5 wt% CNCs (~ 465 ± 5μm)	~1.62	~1.75	~1.95	/	N.A	

	80 (10 wt% KOH)	Raney-Ni	NiFe-LDH	80 wt% ZrO_2/5 wt% CNCs (~ 465 ± 5μm)	~1.75	1.9	2.3	~1.2	300 h at 0.6 A/cm^2, stable between 1.81 V and 1.84 V	
AEL	80 (24 wt% KOH)	Raney-NiMo	Raney-Ni	Zirfon Perl UPT 500	~1.73	~1.95	~2.4	~1.1	N.A	*Energy Environ. Sci.*, 12, 3313-3318 (2019)
AEL	80 (30 wt% KOH)	Raney-NiMo	Raney-Ni	Zirfon Perl UPT 500	/	~1.9	~2.3	~1.3	N.A	*J. Electrochem. Soc.* 163, F3197 (2016)
AEL	80 (30 wt% KOH)	Raney-Ni	NiFe-LDH	300 μm-thick Z80 separator (ZrO_2 and PPSU-based film)	/	~1.8	~2.1	~1.8	300 h at 1 A/cm^2, approximately stable between 1.80 V and 1.83 V	*J. Membrane Sci.* 616, 118541 (2020)
AEL	80 (7 M, *ca.* 30 wt%, KOH)	hot-dip galvanizing treated Ni mesh (Zn dealloyed)	hot-dip galvanizing treated Ni mesh (Zn dealloyed)	140 μm-thick polyethersulfone ultrafiltration membrane (Pall Corporation, Supor®-200)	~1.69	~1.78	~1.93	~2.5	N.A	*Int. J. Hydrogen Energy* 43, 11932–11938 (2018)
PEM	50 (0.5 M H_2SO_4)	20 wt% Pt/C (0.2)	$Ta_{0.1}Tm_{0.1}Ir_{0.8}O_{2-\delta}$ (0.2)	Nafion 117	~1.61	1.766	1.935	/	500 h at 1.5 A/cm^2 *(~0.2 mV/h decay)*	*Nat. Nanotechnol.* 16, 1371–1377 (2021)
PEM	80 (H_2O)	Pt black (3)	$IrRuO_x$ (3)	Nafion 115	/	~1.67	1.84	/	N.A	*Nano Energy* 47, 434–441 (2018)
PEM	60 (H_2O)	Pt/C (1.5)	$Y_{1.75}Ca_{0.25}Ru_2O_7$ (4.1)	Nafion 212	~1.58	~1.67	/	/	16.7 h at 0.2 A/cm^2 *(~9 mV/h decay)*	*Appl. Catal. B: Environ.* 260,

PEM*	60 (H_2O)	Pt/C (1.5)	IrO_2 (3)	Nafion 212	~1.64	~1.75	/	/	N.A	118176 (2020)
PEM*	80 (H_2O)	Pt/C (2)	IrO_2 (2.2)	Nafion 115	~1.55	1.64	1.83	/	N.A	*ACS Catal.* 6, 2626 (2016)
PEM	80 (H_2O)	Pyrite FeS_2 (5)	IrO_2 (2.2)	Nafion 115	~2	2.1	2.23	0.5	N.A	
PEM	80 (H_2O)	FeMoS(mw) (4)	Ir black (2)	Nafion 212	1.77	1.85	/	/	24 h at cycling between 0.05 and 0.5 A/cm^2 (*~1.9 mV/h decay*)	*ACS Catal.* 10, 14336−14348 (2020)
PEM	80 (H_2O)	Pt/C (0.5)	$Ir_{0.7}Ru_{0.3}O_x$ (1.8)	Nafion 115	~1.54	1.66	~1.84	/	4 h at 0.2 A/cm^2	*Adv. Energy Mater.* 9, 1802136 (2018)
PEM	38 (H_2O)	Pt	Ir	Nafion 212	~1.7	~1.8	1.97	/	N.A	*Energy Environ. Sci.* 10, 2521-2533 (2017)
PEM	80 (H_2O)	40 wt% Pt/C (1)	IrO_x-Ir (1)	Nafion 212	~1.57	~1.64	~1.77	/	100 h at 2 A/cm^2, fluctuated between 1.78-1.8 V	*Angew. Chem. Int. Ed.* 55, 742-746 (2016)
PEM	80 (H_2O)	60 wt% Pt/C (0.5)	IrO_2@TiO_2 (Ir-loading: 0.4)	Nafion N212 or N115	~1.6	1.67	~1.78	~4.3	N.A	*Appl. Catal. B: Environ.* 269, 118762, (2020)
PEM	80 (H_2O)	46.7 wt% Pt/C (~0.35 mg Pt)	IrO_2/TiO_2 (75 wt% iridium) (~2 mg Ir)	Nafion 212 (50 μm)	~1.52	1.57	~1.65	>6	N.A	*J. Electrochem.* Soc. 163, F3179 (2016)

AEM	50 (H_2O)	Pt black (3)	NiCoOx:Fe (3)	FAA-3 (FumaTech)	~2.1	~2.45	/	~0.37	3 h at 0.2 A/cm^2 *(~150 mV/h decay)*	*ACS Catal.* 9, 7–15, (2019)
AEM	42-45 (1M KOH)	Pt/C (1)	$Ni_{0.75}Fe_{2.25}O_4$	Sustainion X37-50	~1.65	1.75	1.9	/	21 h at 0.5 A/cm^2 *(~9.5 mV/h decay)*	*Chem. Eng. J.* 420, 127670 (2021)
AEM*	42-45 (1M KOH)	Pt/C (1)	IrO_2 (4)	Sustainion X37-50	~1.67	1.84	/	/	N.A	
AEM	50 (1M KOH)	Pt/C	NiFeV LDH (3.95)	Sustainion X37-50	~1.56	~1.65	~1.79	/	100 h at 0.5 A/cm^2 *(~2 mV/h decay)*	*Small* 17, 2100639 (2021)
AEM	30 (0.1M KOH)	40 wt% Pt/C (1)	$Cu_{0.81}Co_{2.19}O_4$	Fumasep FAA-3-PE-30	~1.87	/	/	/	100 h at 0.1 A/cm^2 *(~0.2 mV/h decay)*	*ACS Appl. Mater. Interfaces* 10, 38663-38668 (2018)
AEM	45 (1M KOH)	40 wt% Pt/C (1)	IrO_x (4)	Sustainion X37-50	/	~1.75	~1.87	/	64 h at 0.5 A/cm^2	*Appl. Catal. B: Environ.* 278, 119276 (2020)
AEM	50 (1M KOH)	40 wt% Pt/C (1)	$Cu_{0.5}Co_{2.5}O_4$ (10)	Sustainion X37-50	~1.65	~1.74	/	/	100 h at 0.5 A/cm^2 *(~0.6 mV/h decay)*	*J. Mater. Chem. A* 8, 4290–4299 (2020)
AEM	80 (1M KOH)	Pt/C (1.3)	NiFe-LDH (2.5)	Sustainion X37-50	~1.52	1.59	/	/	6 h at 1 A/cm^2 *(~3.3 mV/h decay)*	*ACS Catal.* 10, 1886-1893 (2020)
AEM*	60 (1M KOH)	Pt/C (1.2)	IrO_x (1.9)	Sustainion X37-50	~1.55	1.67	/	/	6 h at 1 A/cm^2 *(~10 mV/h decay)*	
AEM	60 (1M KOH)	$MoNi_4/MoO_2$	Ni_2P @ $FePO_xH_y$	Sustainion X37-50	~1.75	1.84	/	/	72 h at 1.75 V, decrease from 0.5 to ~0.45 A/cm^2	*Appl. Catal. B: Environ.* 306, 121127 (2022)

AEM	50 (1 M KOH)	Raney NiMo (25.2)	$Ni(OH)_2$-Fe (4)	Tokuyama A201 (28 μm)	~1.7	~1.85	2.046	~1.8	486 h at 1 A/cm^2, approximately stable between 1.92 V and 1.85 V	*ACS Appl. Energy Mater.* 5, 2221–2230 (2022)
AEM	60 (H_2O)	PtRu/C (Pt, Ru loading: 2,2)	IrO_2 (~4)	HTMA-DAPP*(~50 μm)	~1.65	1.79	/	/	24 h at 0.2 A/cm^2, stable for the first 8 h, then increase by 100 mV	*ACS Appl. Mater. Interfaces* 13, 50957−50964 (2021)
AEM	90 (6M KOH)	Pt/C (1.5)	IrO_2 (1.5)	NPBI^ ion-solvating membrane	~1.57	~1.69	~1.9	~2.5	298 h at 0.5 A/cm^2, fluctuated between 1.66-2 V	*J. Membrane Sci.* 643, 120042 (2022)
AEM	50 (0.3 M KOH)	PtNi (3.0 ± 0.1)	IrO_x (3.0 ± 0.1)	XION™ Composite-72–10CL-30 μm	/	1.79	/	/	720 h at 1A/cm^2, almost stable at 1.8 ± 0.05 V	*Electrochim. Acta* 409, 140001 (2022)
AEM	80 (1 M KOH)	46.6% Pt/C (0.5), PFBP-14 ionomer	IrO_2 (2), PFTP-8 ionomer	PFTP-13 ♦	/	~1.6	~1.67	7.68	N.A	*Energy Environ. Sci.*, 14, 6338 (2021)
AEM	60 (1 M KOH)	46.6% Pt/C (0.5), PFBP-14 ionomer	IrO_2 (2), PFTP-8 ionomer	PFTP-13	~1.61	~1.67	~1.78	~5.2	1100 h at 0.5A/cm^2, fast increased to ~2.3V, then fluctuated between 2.2-2.0 V	
AEM	70 (1 M KOH)	60 wt.% Pt/C (0.4 mg Pt)	60 wt.% PtRu/C (0.4 mg Pt-Ru metal)	AEM based on quaternised poly-carbazole (QPC-TMA) (50 μm)	~1.55	~1.62	~1.74	4.5	10000 s at 1.6 V cell voltage, current density decreased by ~50 %	*Energy Environ. Sci.*, 13, 3633-3645 (2020)

AEM	20 (1 M KOH)	NiMo-NH_3/H_2 (3)	Fe-NiMo-NH_3/H_2 (3)	Sustainion X37-50	~1.66	1.77	/	/	25 h at 0.5 A/cm^2, Increased from ~1.66V to ~1.72V	*Adv. Energy Mater.* 10, 2002285 (2020)
AEM	80 (1 M KOH)	NiMo-NH_3/H_2 (3)	Fe-NiMo-NH_3/H_2 (3)	Sustainion X37-50	1.52	1.57 V	/	/	N.A	
AEM	60 (1 M KOH)	NiFeCo (2)	$NiFe_2O_4$ (2)	Sustainion 37-50	~1.8	1.9	/	~1.7	1950 h at 1 A/cm^2, almost stable at 1.9-1.92 V	*Int. J. Hydrogen Energy* 42, 29661–29665 (2017)
AEM	90 (H_2O)	47% Pt/C (0.94 mg Pt)	fluoride-incorporated Fe_xNi_yOOH on Ni foam (4.8)	poly(aryl piperidinium) hydroxide exchange membrane (PAP-TP-85, 20 μm)	~1.65	~1.8	/	/	70 h at 0.5 A/cm^2 *(~1.81 mV/h decay)*; 160 h at 0.2 A/cm^2 *(~0.56 mV/h decay)*	*ACS Catal.* 11, 264−270 (2021)
AEM	85 (H_2O)	PtRu/C (50wt% Pt and 25wt% Ru) (2)	Ni_2Fe_1 (3)	HTMA-DAPP*(26 μm)	~1.54	~1.61	~1.71	~3.5	10 h at 0.2 A/cm^2, rapidly increased to ~2.3 V	*Nat. Energy* 5, 378–385 (2020)
AEM	60 (1 M KOH)	PtRu/C (50wt% Pt and 25wt% Ru) (2)	IrO_2 (2.5)	HTMA-DAPP*(26 μm)	~1.8	~1.95	/	~1.15	100 h at 0.2 A/cm^2, rapidly increase to 2.1 V within initial 3 h, then become stable	

Note: * Commercial PGM (e.g., Pt, Ir and Ru)-based ELs; † ccm: Cubic Centimeter per Minute (cm^3/min, equals mL/min); ⁕hexamethyl trimethyl ammonium-functionalized Diels-Alder polyphenylene; ∧ Poly[2,2'-(1,4-naphthalene)-5,5'-bibenzimidazole]; ♦ poly(fluorenyl-co-terphenyl piperidinium-13).

N.A.: Not available (Authors doesn't provide test data).

Carbon paper (CP) was adopted as GDL at cathode side in our AEL assembly, while no GDL was used at anode side.

The cell voltage values with the symbol (~) are extracted from their corresponding polarization plots.

The operating pressure of ELs is not always mentioned.

Calculation of mass and price activities

Mass activity

The mass activities of the cathodes were calculated at the overpotential of -100 mV (vs. RHE) by dividing the current density recorded by the amount of PGM metals (Ru or Pt):

$$j_{mass}^{Ru@Cu-TM} = \frac{258\ (mA/cm^2)}{0.053\ (mg/cm^2)} = 4.87\ A/mg\ ;$$

$$j_{mass}^{Pt/C-TM} = \frac{51\ (mA/cm^2)}{0.1\ (mg/cm^2)} = 0.51\ A/mg$$

Price activity

The price activities of the cathodes were calculated by dividing their mass activity, by the price of Ru or Pt:[27]

$$j_{price}^{Ru@Cu-TM} = \frac{4.87 * 1000\ (A/g)}{6.88\ (\$/g)} = 707.8\ A/\$$$

$$j_{price}^{Pt/C-TM} = \frac{0.51 * 1000\ (A/g)}{34.36\ (\$/g)} = 14.8\ A/\$$$

Estimation of operating cost for H_2 production in our AELs

In this section, the operating cost for the H_2 production in our AELs were explicitly calculated by using the experimental data acquired during the stability test of our Ru@Cu-TM || 5-stacked SSMs AEL using Zirfon Perl UTP220 diaphragm. In particular, an average voltage of 1.71 V was measured for the AEL operating at 1 A/cm^2 for 500 h.

Therefore:

Rate of H_2 generation (*a*) on 1 cm^2 electrode at 1 A/cm^2

$$a = \frac{j}{F \times n} \times N_A$$

$$= \frac{(Current\ density)}{F \times 2} \times 6.022 * 10^{23}$$

$$= \frac{1}{96485 \times 2} \times 6.022 * 10^{23}$$

$$= 3.12 \times 10^{18}\ \text{H}_2\ \text{molecules/(cm}^2 \times \text{s)}$$

For 500 h, 5 cm^2 AEL can produce the following H_2 mass and volume:

$$\text{Mass (H}_2) = \frac{a \times S_{electrolyzer} \times t}{N_A} \times M_w$$

$$= \frac{(H2\ production\ rate) \times Electrolyzer\ area \times Time}{6.022 * 10^{23}} \times \text{(Molar mass of H}_2)$$

$$= \frac{3.12 * 10^{18} \times 5 \times (500 * 3600)}{6.022 * 10^{23}} \times 2.016$$

$$= 93.26\ \text{g H}_2$$

(*Note: Water consumption ~40 mL/day @5 cm^2*)

Considering only the electricity consumed by electrolyzer component, the operating cost of H_2 per kilogram of H_2 is (see TEA analysis in the subsequent section):

Operating cost (H_2/kg) = energy consumption × electricity cost

$$= \frac{1\ \text{A/cm}^2 \times 5\ \text{cm}^2 \times 1.71\ \text{V} \times 500\ \text{h}}{1000 \times 0.09326\ \text{kg}} \times \$0.02/\text{Kw h}$$

$$= 45.84\ \text{Kw h / kg H}_2 \times \$0.02/\text{Kw h}$$

$$= \$\ 0.92\ /\ \text{kg H}_2$$

Techno-economic analysis of H_2 production at MW-scale AEL plant-level

The following section, reported as a bullet list for the sake of clarity, aims at guiding the reader in the navigation and understanding of the attached Excel file, in which all the calculations related to the TEA of our AEL technology are gathered.

Sheet "Unitary cost of DEP components": evaluation of the unitary cost of the cell components, including:

1. determination of the unitary prices of raw materials composing each component of the DEP (**Supplementary Table 7**). In particular, for cathodes and anodes, determination of the mass loading (by ICP-OES) of the elements found in the catalytic layer;
2. from the data collected in the previous point, calculation of the raw materials cost associated to each synthesized/fabricated cathode/anode, by multiplying the unitary price of the metal by its mass loading;
3. evaluation of the fabrication cost of cathodes and anodes according to the synthetic/fabrication procedure (refer to the Experimental, section 4 of the main text) and the related parameters and costs (**Supplementary Table 8**). In the following, the equations used for calculating each single contribution to the overall cost of manufacture are reported:

$$C_{\mathrm{CuO\ chem\ dep}} = (m_{\mathrm{bath}} \times C_p \times \Delta T) \times C_{\mathrm{Electricity}} + V_{\mathrm{NH4OH}} \times C_{\mathrm{NH4OH}}$$

$$C_{\mathrm{CuO \rightarrow\ Cu^0\ reduction}} = j \times E_{\mathrm{Applied}} \times t_{\mathrm{Reduction}} \times C_{\mathrm{Electricity}}$$

$$C_{\mathrm{Ru\ electrodeposition}} = j \times E_{\mathrm{Applied}} \times t_{\mathrm{Electrodeposition}} \times C_{\mathrm{Electricity}}$$

$$C_{\mathrm{NiFe\ chem\ dep}} = (m_{\mathrm{Chemical\ bath}} \times C_p \times \Delta T) \times C_{\mathrm{Electricity}}$$

where C_X are the calculated or standardized costs of the x procedure/input (i.e., CuO chem dep = synthesis of CuO NPLs; CuO → Cu^0 redution = electroreduction of Cu NPLs ; Ru electrodeposotion = electrodeposition of Ru NPs; NiFe chem dep = chemical deposition of Ni-Fe hydroxides with low crystallinity), $m_{\mathrm{Chemical\ bath}}$ is the mass of the chemical bath solution, C_p is the heat capacity of the solution (approximated to that of pure water, **Supplementary Table 8,10**), V_X are the volumes of x reactants, j, E_{Applied} and t are the current density, applied voltage and time of electrochemical reduction/deposition procedures;

4. sum of raw materials and manufacturing costs for each possible cathode and/or anode, as to obtain the unitary cost ($/$cm^2$) of each component of the DEP.

Sheet "CAPEX - 1 MW ideal AEL": Evaluation of the CAPEX of a 1 MW-scale AEL plant based on our single cell technology:

1. starting from the average cost ($ kW^{-1}) of a 1+ MW AEL plant and considering the cost breakdown for a 1 MW AEL plant reported by IRENA[28], calculation of the average CAPEX for the deployment of a standard 1 MW AEL plant;
2. considering the average CAPEX of a standard 1 MW AEL plant (*ca*. 600000 $) obtained in the previous point, calculation of the actual cost of each AEL plant component, considering the percentage cost breakdown reported by IRENA;
3. benchmarking of the cost of a single cell (single DEP), considering the performance of the Korean Institute of Energy Research (KIER) AEL plant reported by Lee et al. as a reference case (main operational parameters are reported in **Supplementary Table 9**);
4. calculation of the actual power of our single cell lab-scale AEL (5 cm^2) ($P_{\mathrm{Lab\text{-}scale\ AEL}}$) and calculation of the electrode area of our AEL needed to achieve the same performance reported for the KIER AEL plant (assuming the same number of stacks and cells per stack) ($A_{\mathrm{Single\ cell\text{-}Ideal\ 1\ MW\text{-}scale\ AEL}}$);

$$P_{\mathrm{Lab-scale\ AEL}} = E_{\mathrm{Cell}} \times J \times A_{\mathrm{Lab-scale\ AEL}}$$

$$A_{\text{Single cell}-1\text{ MW}-\text{scale AEL}} = \frac{P_{\text{Single cell}-\text{KIER}}}{P_{\text{Lab}-\text{scale AEL}}/A_{\text{Lab}-\text{scale AEL}}}$$

where P_X indicates the power of the x single cell AEL system (our or KIER ones) and A_X is their electrodes' area;

5. calculation of the overall DEP cost, multiplying the unitary cost of each cathode/anode/diaphragm by $A_{\text{Single cell-Ideal 1 MW-scale AEL}}$;
6. postulating a constant cost of cell manufacture and replacing the CAPEX associated to diaphragm cathode and anode, calculation of the CAPEX of a single DEP based on different electrode combinations of the here presented cathodes and anodes;
7. assuming constant all other CAPEX contributions (i.e., costs of system components related to the stack and BoP), evaluation of the CAPEX of a 1 MW AEL plant based on different cathode/anode combinations reported in this work;
8. Evaluation of the annual CAPEX for the 1 MW AEL plant described in the previous point by applying a CRF, calculated as described in the Experimental, section 4 of the main text. The discount rate (i_{Rate}) and plant lifetime (n) has been set at 4.5% and 10 years, respectively.

Sheet "OPEX - 1 MW ideal AEL": evaluation of the OPEX of a 1 MW AEL plant based on our single cell technology:

1. according to standardized procedures for OPEX calculation, splitting of the annual OPEX in different entries, namely electricity, water, labor, maintenance and ancillary expenses;
2. calculation of electricity-related expenses, according to the following equations:

$$I_{\text{Tot}} = j \times A_{\text{Single cell}-\text{Ideal 1 MW}-\text{scale AEL}} \times n_{\text{Cells per stack}} \times n_{\text{Stacks per system}}$$

$$P_{\text{AEL (gross)}} = I_{\text{Tot}} \times E_{\text{Cell}} \times t_{\text{Annual AEL operation}}$$

$$\text{OPEX}_{\text{Electricity}} = P_{\text{AEL (gross)}} \times C_{\text{Electricity}}$$

where I_{Tot} is the total current delivered by the AEL, $n_{\text{Cells per stack}}$ is the number of cells per stack, $n_{\text{Stacks per system}}$ is the number of stacks per 1 MW AEL plant, and $t_{\text{Annual AEL operation}}$ is the time of actual annual operation of the AEL plant, set at 84,000 h (i.e., 350 day per year, **Supplementary Table 10**). $P_{\text{AEL (gross)}}$ is the power fed to the AEL plant to carry out the water splitting reactions; $\text{OPEX}_{\text{Electricity}}$ is the AEL plant OPEX contribution of the electricity fed to the AEL plant to carry out the water splitting process. $C_{\text{Electricty}}$ is the cost of the electricity

According to reports on currently operative AEL plants,[28] the energy consumption for the water splitting process accounts only for the 50% of the overall energy fed to the whole AEL plant, with BoP auxiliaries (e.g., gas and liquid circulation, and gas compression) requiring a similar energy fed. Doubling the $\text{OPEX}_{\text{Electricity}}$ allows to obtain an overall OPEX more adherent to a real case scenario.

NOTE: the AEL's gross power calculations have been run considering a parallel electrical configuration of the cells. Although this might not be representative of the real configuration of a MW-scale AEL plant, it anyway returns a reliable value in terms of power fed to the AEL plant;

3. calculation of the expense related to the water consumption (OPEX_{H2O}):

$$m_{\text{H2O consumed (per year)}} = m_{\text{Produced H2 (per year)}} \times m_{\text{Average H2O consumption per kg of H2}}$$

$$\text{OPEX}_{\text{H2O}} = m_{\text{H2O consumed (per year)}} \times C_{\text{H2O}}$$

where $m_{\text{H2O consumed (per year)}}$ is the mass of the water consumed per year, the average water consumption per kg of produced H_2, $m_{\text{(Average per kg of H2)}}$ is set at 10 L kg^{-1} and the average cost of water (C_{H2O}) is set at 0.0014 \$ L^{-1} (**Supplementary Table 10**);

4. labor, maintenance and other ancillary expenses are instead calculated as standardized percentages of the initial CAPEX of the whole system. Specifically, the annual OPEX

associated to labor, maintenance and ancillary activities are estimated to equal the 0.3, 2.5 and 1% of the initial CAPEX, respectively.

Sheet "Annual H_2 productivity at 1 MW":

Refer to the dedicated paragraph in the Experimental, section 4 of the main text.

Sheet "H_2 production cost":

Refer to the dedicated paragraph in the Experimental, section 4 of the main text.

Sheet "Ru availability": reports a rough assessment of the sustainability of the technology in terms of raw materials worldwide availability versus the deployed power envisaged according to IRENA's Energy Scenarios:[28]

1. Starting from the determined Ru mass loading and considering the electrode area, the number of cells per stack and the number of stacks per electrolyzer (all these parameters have been calculated/assumed in the sheets "CAPEX - 1 MW ideal AEL" and "OPEX - 1 MW ideal AEL"), calculation of the Ru content per deployed power according to the formula:

$$m_{Ru}/P_{AEL\,(Gross)} = \frac{Loading_{Ru} \times \mathrm{A_{Single\ cell}} \times \mathrm{n_{Cells\ per\ stack}} \times \mathrm{n_{Stacks\ per\ system}}}{P_{AEL\,(Gross)}}$$

   where $\mathrm{Loading_{Ru}}$ is the Ru mass loading assessed by ICP-OES on our electrodes (*ca.* 53 µg cm^{-2}) while all the other parameters retain the above- described meaning.

2. Considering the cost and the worldwide production/reserves of Ru ("Additional data") and taking into account the total electrolyzers' power to be deployed globally by 2030 and/or 2050 according to IRENA's Planned/Transforming Energy Scenarios,[28] calculation of the mass of Ru demand related to our technology.
3. As with Ru, calculation of the demand/supply ratio of Pt when considering PEMELs as the technology of reference for the deployment of the electrolysis power envisage by IRENA's Planned/Transforming Energy Scenarios

Ni-based commercial AEL system: The above reported description applies also to the Excel file dedicated to the commercial Ni-based system (1 Cell Alkaline Electrolysis Stack with 12 cm^2 active size - Fuel Cell Corp.).[29] As the electrochemical performance of the system has been recorded up to a current density of 500 mA/cm^2, the cell voltage at 1 A/cm^2 has been linearly extrapolated from the trend reported in **Supplementary Fig. 44**, green line.

Supplementary Table 7. Cost of raw materials composing the DEP. References for cost determination can be found in the supplemental Excel file.

**Cathodes**		**Cost**	
Supports	TM – Titanium mesh	100	\$/m^2
	NM – Nickel mesh	48.50	\$/m^2
	SSM – Stainless steel mesh	11.20	\$/m^2
Catalyst layer	Ti	28	\$/kg
	Cu	10.50	\$/kg
	Ru	18.50	\$/g

Anodes			
Supports	NF – Nickel foam	30.97	$/m^2
	SSM – Stainless steel mesh	11.20	$/m^2
Catalyst layer	Ni	26.50	$/kg
	Fe	0.15	$/kg
**Diaphragms**			
	Zirfon Perl UTP220	380	$/m^2

Supplementary Table 8. Operational parameters for cathode and anode manufacturing and related cost of reagents and energy. References for cost determination can be found in the supplemental Excel file.

Cathodes fabrication		Parameters		
CuO chemical deposition	*Heating*^{a}	Temperature	90	°C
		Time	2	h
		$V_{Chemical\ bath}$	8	mL/cm^2
		Electricity cost	0.02	$/kWh
	NH_4OH	Volume	0.4	mL/cm^2
		Cost	26.80	$/L
CuO to Cu^0 electroreduction	*1 M NaOH*	Volume	25	mL/cm^2
		Cost	101.7	$/kg[1]
	Electrochemical CuO to Cu^0 reduction	Current density	10	mA/cm^2
		Average $E_{Applied}$	-0.81	V vs. Ag/AgCl
		CP time	0.67	h
Ru electrodeposition		$E_{Applied}$	1.22	V vs. Ag/AgCl
		Current density	100	mA/cm^2
		CP time	3	h
**Anodes fabrication**				
Ni and Fe chemical deposition	*Heating*^{a}	Temperature	80	°C
		Time	3	h
		$V_{Chemical\ bath}$	4	mL/cm^2
		Electricity cost	0.02	$/kWh

[a] The cost of the heating of the chemical baths was calculated considering the energy required to heat the baths from room temperature up to the desired temperature, as $\Delta Q = m_{Chemical\ bath} \times C_p \times \Delta T$, where ΔQ is the heat energy, ΔT is the temperature change, $m_{Chemical\ bath}$ is the mass of the chemical bath solution and C_p is the heat capacity of the solution (approximated to that of pure water, C_{pH2O}). A perfect thermal insulation of the bath itself was postulated in the calculation of ΔQ. C_{pH2O} = 4.187 J g^{-1} °C^{-1}, while the water density (d_{H2O} = 1 mg mL^{-1}) was used to calculated $m_{Chemical\ bath}$ from the bath volume.

Supplementary Table 9. Operational parameters of the KIER AEL plant reported by Lee et al.[30] Data used to benchmark the cost of a 1 MW AEL plant based on our single cell technology

**Operational parameter**		
Cell voltage	1.8	V
Current density	1	A/cm^2
Electrode area	700	cm^2
Cell power	1.8 (1260)	W/cm^2 (W)
Cells per stack	200	units
Stacks in the system	5	units
Gross system power	1.26	MW
Stack (cell) energy efficiency	82	% (HHV)
Stack power	1.03	MW

Supplementary Table 10. Miscellaneous data used for the TEA

**Parameter**		
Prices and financial assumptions		
Cost of electricity (www.irena.org/publi cations)	0.02	\$/kWh
Cost of process water (https://doi.org/10.1016/j.xcrp.2020.100209)	0.0014	\$/L_{H2O}
Discount rate [31]	4.5	%
Plant lifetime [31]	10	Year
Capital recovery factor[a]	0.126	
Stack CAPEX of the 1 MW AEL plant [28]	270	\$/kW
1 MW AEL plant CAPEX[b]	600000	\$
Additional AEL's operative parameters		
H_2 Faradaic efficiency[c]	100	%
Annual operative time	8400 (350)	h (day)
Process water usage [32]	10	L/kg_{H2}
Miscellaneous constants		
Water heat capacity	4.187	J/(g×°C)
H_2 molecular mass	2.016	g/mol
Faraday's constant	96485	C/mol_{e-}
Number of exchanged electrons for the HER	2	mol_{e-}/mol_{H2}

[a] Calculated from the discount rate and the forecasted plant lifetime according to the equation reported in the Experimental, section 4 of the main text;

[b] Calculated according to point 1 of "Sheet "CAPEX - 1 MW ideal AEL" procedure;

[c] Approximated to 100% in all calculation, in accordance with the GC results discussed in the main text (**Supplementary Fig. 11**)

Supplementary Table 11. Unitary cost ($/cell) of a single cell of *ca.* 700 cm^2 for different cell configurations.

Cathode \ Anode	Case 1: NiFe@NF	Case 2: 5-stacked SSMs
Case 1: Ru@Cu-TM	157.13	158.95
Case 2: Ru@Cu-Ti@NM	150.54	-
Case 3: Ru@Cu-Ti@SSM	-	152.42
IRENA/KIER single cell[a]	153.90	

[a] Both cathode and anode are Ni-coated perforated stainless-steel foils

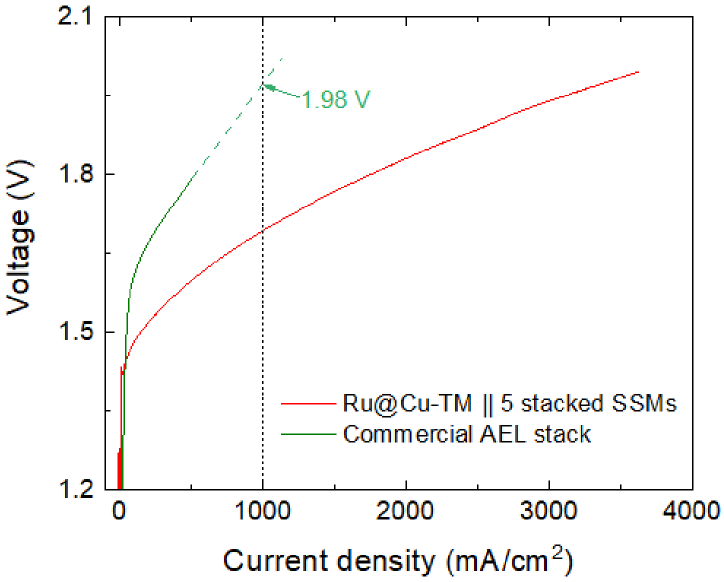

Supplementary Fig. 44. Polarization curves measured for a commercial-like AEL (Ni-based electrodes) and Ru@Cu-TM || 5-stacked SSMs (this work). The commercial AEL (purchased from Fuel Cell Corp.) has a big working area of 12 cm^2, which exceeds the maximum current limit of our potentiostat if tested up to 1 A/cm^2. We therefore used the experiment data (solid line) to extrapolate data (dashed line) for the TEA calculations. Details about the calculations could be found in the Supplementary Information and Data 2.

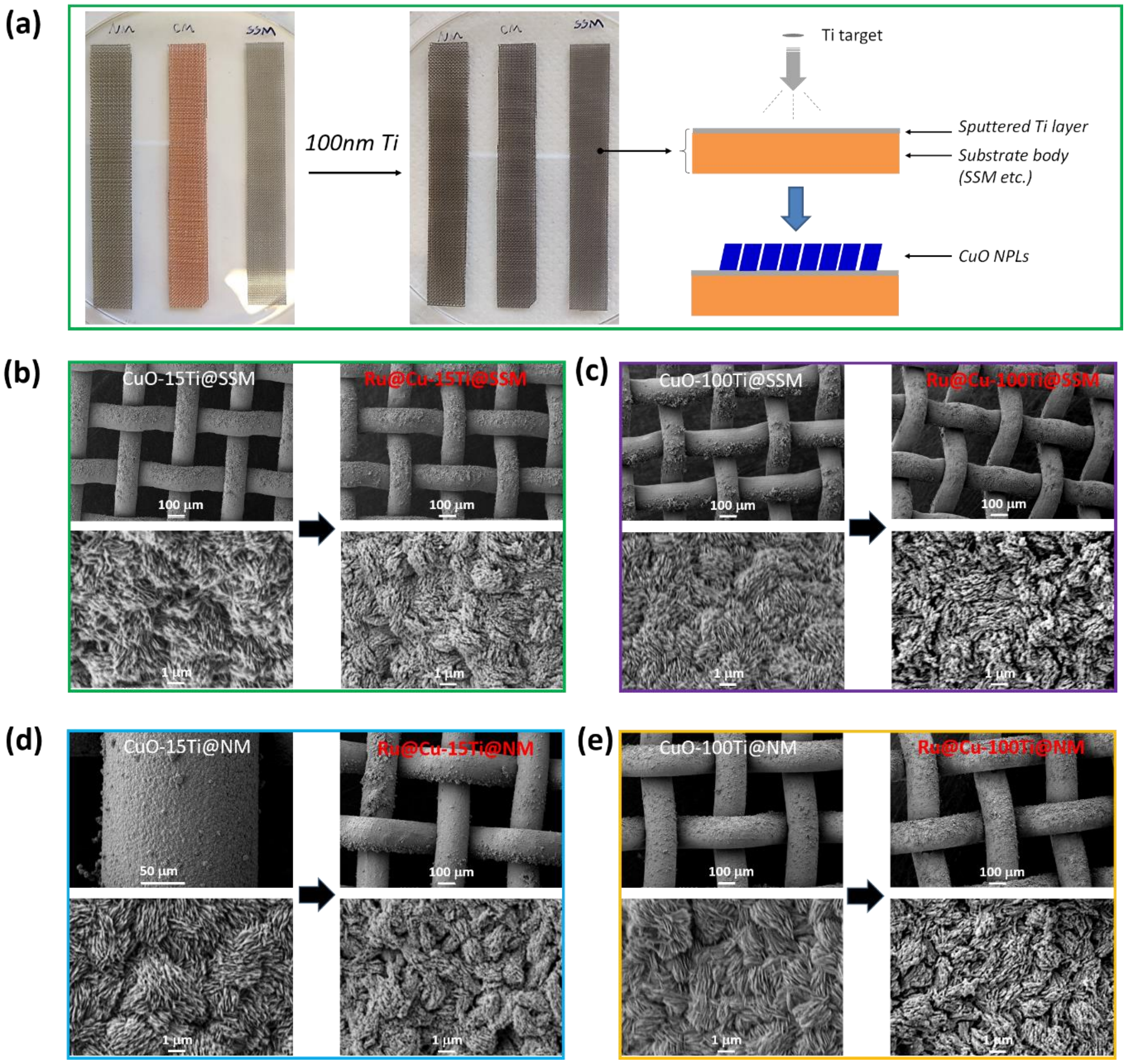

Supplementary Fig. 45. Investigation on synthesis of Ru@Cu on other metallic mesh substrates. (a) Scheme sketching the sputtering of Ti layer on metallic substrates alternative to TM (namely, NM, SSM and CM), followed by the growth of CuO NPLs *via* chemical bath deposition; (b-e) CuO NPLs growth on different Ti-coated NM and SSM for different Ti thickness (15 nm, 100 nm), and the resulting cathodes obtained by electrodepositing Ru NPs.

To control the growth of CuO NPL on CM and SSM substrates as for the case of TM and NM, a layer of Ti was preliminary sputtered on CM and SSM. **Supplementary Fig. 45** shows that CuO NPLs can grow vertically on Ti-coated NM and SSM. In addition, the thickness of the sputtered Ti layer (15 nm or 100 nm, leading to substrate named 15Ti@NM, 100Ti@NM, 15Ti@SSM and 100Ti@SSM) does not have any significant effect on the morphology of the CuO NPLs, as well as on subsequent electrodeposition of the Ru NPs.

These results shown in **Supplementary Fig. 14,45** indicate that the CuO NPL growth is strongly affected by the chemistry (affinity) of the substrate surface, and surface treatment with sputtered Ti is required to obtain the catalysts characteristics of the cathodes previously developed using TM substrate.

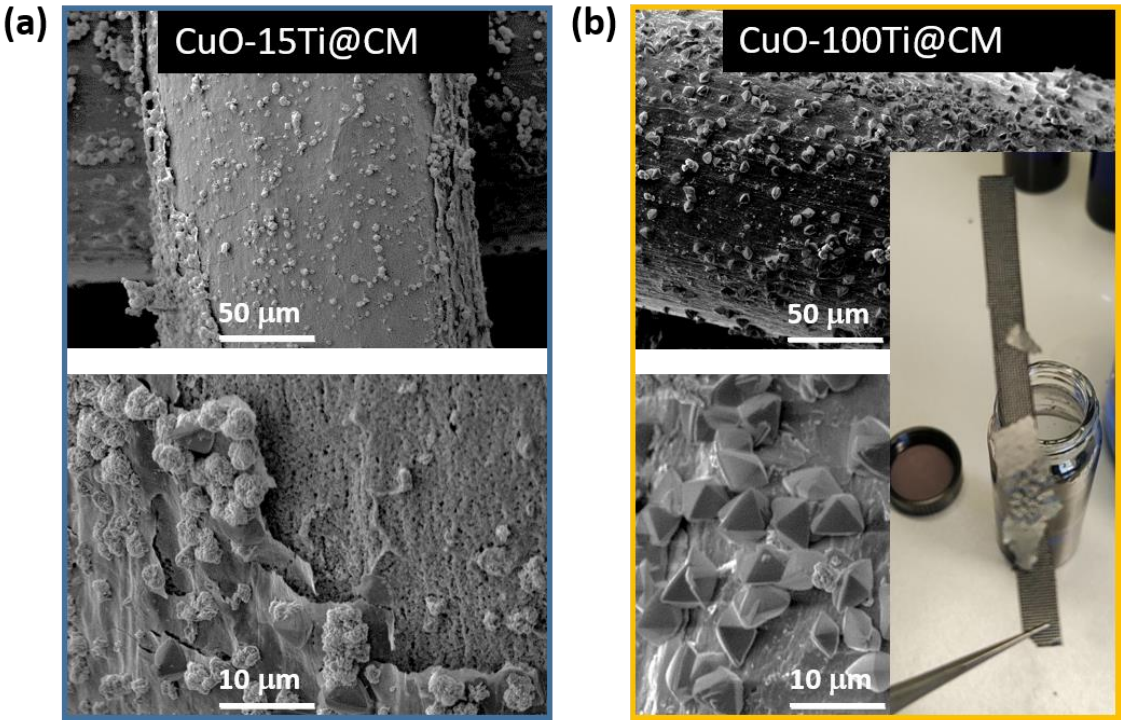

Supplementary Fig. 46. CuO NPLs growth on Ti-coated CM with Ti thickness of (a) 15 nm, and (b) 100 nm. The sputter coated Ti layer peeled off from CM surface, resulting inadequate for the subsequent electrodeposition of the Ru NPs.

CuO NPLs were not able to grow vertically on the surface of Ti-coated CM due to a peeling-off of sputtered Ti during chemical bath deposition.

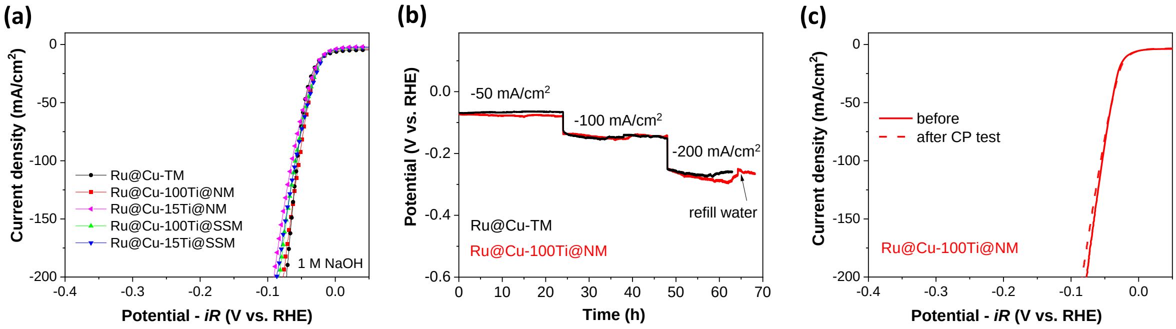

Supplementary Fig. 47. Comparison of HER performance. (a) LSV curves with iR-correction measured for the cathodes fabricated using various substrates (TM, 100Ti@NM, 15 Ti@NM, 100Ti@SSM and 15 Ti@SSM). (b) CP measurements (potential without iR-correction vs. time plots) for Ru@Cu-TM and Ru@Cu-100Ti@NM. (c) LSV curves with iR-correction measured for Ru@Cu-100Ti@NM before and after CP measurements.

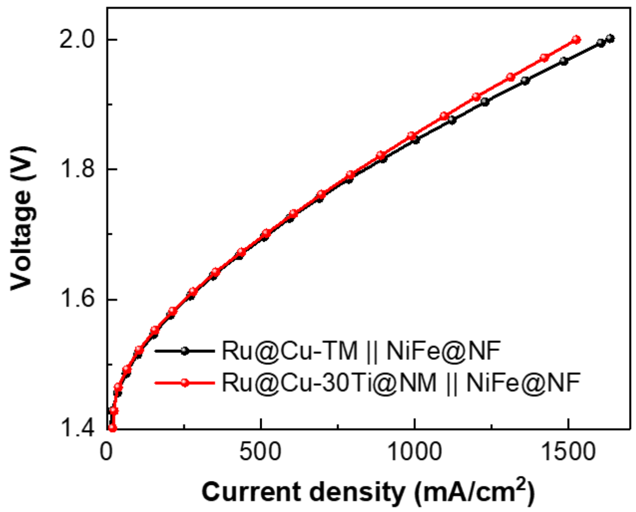

Supplementary Fig. 48. Polarization curves measured for zero-gap AELs based on NiFe@NF anode using different cathodes, Ru@Cu-TM or Ru@Cu-30Ti@NM (electrodes area = 1 cm^2). Test conditions: anode: NiFe@NF; diaphragm: Zirfon Perl UTP 500+; GDLs: CPR at Cat. and none at Ano.; electrolyte: 30 wt% KOH; temperature: 80 °C.

As expected from our previous cathode characterizations using the three-electrode configuration, the replacement of Ru@Cu-TM with its low-cost version Ru@Cu-30Ti@NM did not cause any relevant change of the AEL performance (e.g., 0.5 A/cm^2 at 1.69 V; 1 A/cm^2 at 1.85 V, **Supplementary Fig. 48**).

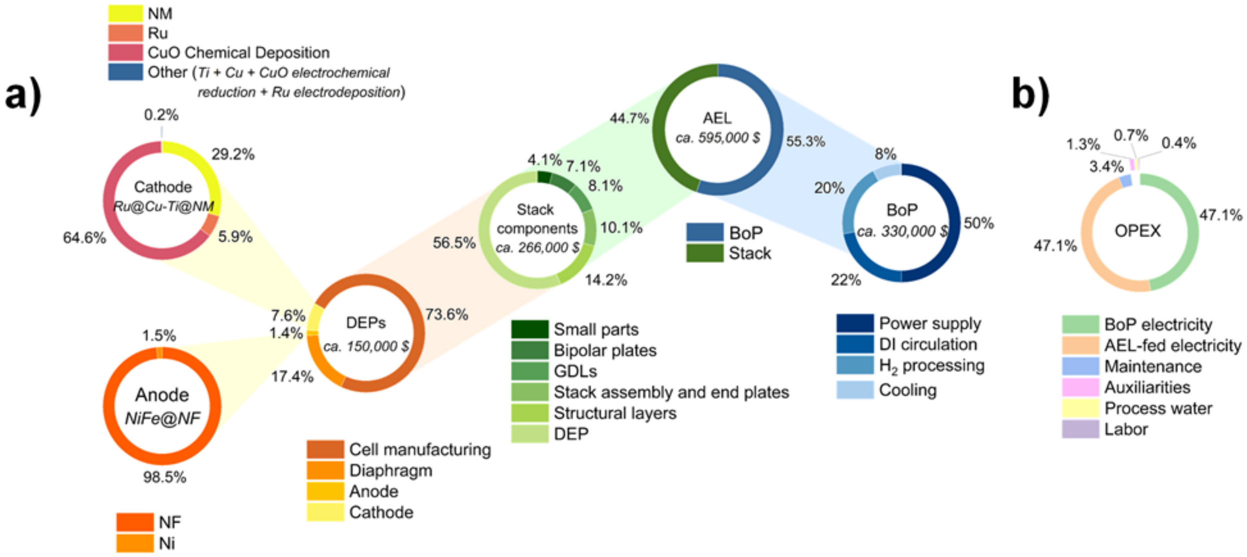

Supplementary Fig. 49. TEA for Ru@Cu-Ti@NM || NiFe@NF AEL operating at 1 MW-scale. (a) CAPEX and (b) OPEX of a 1 MW AEL plant (1000 cells) based on the Ru@Cu-Ti@NM || Zirfon Perl UTP 500+ || NiFe@NF cell configuration.

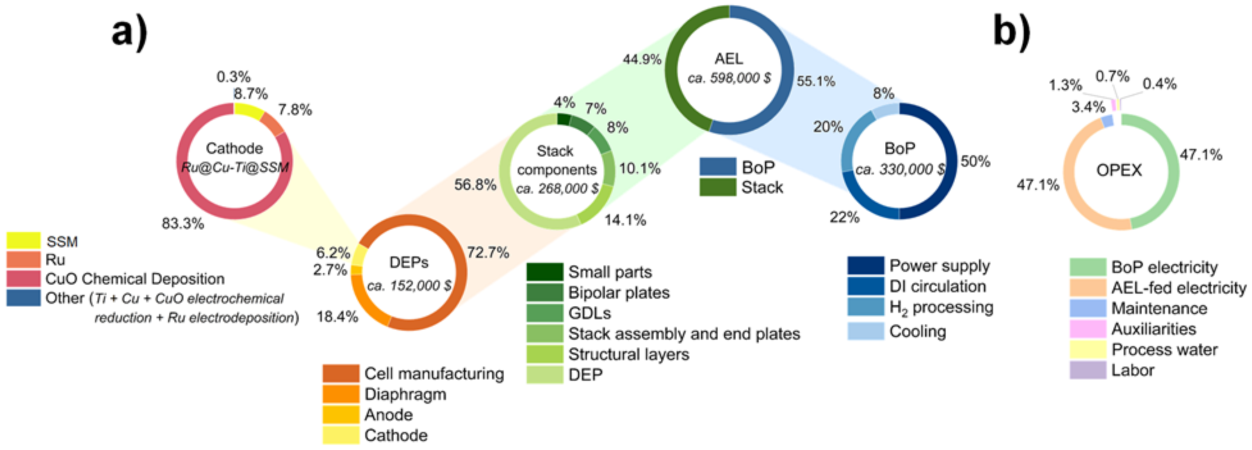

Supplementary Fig. 50. TEA for Ru@Cu-Ti@SSM || 5-stacked SSMs AEL operating at 1 MW-scale. (a) CAPEX and (b) OPEX of a 1 MW AEL plant (1000 cells) based on the Ru@Cu-Ti@SSM || Zirfon Perl UTP 220 || 5-stacked SSMs cell configuration.

Supplementary Table 12. Potential (V) required by different single cells to deliver a 1 A/cm^2 current density

**Cathode** \ **Anode**	*Case 1*: NiFe@NF	*Case 2*: 5-stacked SSMs
Case 1: Ru@Cu-TM	1.71	1.71
Case 2: Ru@Cu-Ti@NM	1.83[a]	-
Case 3: Ru@Cu-Ti@SSM	-	1.71

[a] Diaphragm used: Zirfon Perl UTP 500+, differently from all other configurations that used the Zirfon Perl UTP220.

Supplementary Table 13. List of possible cathode/anode combination determining the DEPs. Unless differently indicated, tested configurations used Zirfon Perl UTP220 as diaphragm.

**Cathode** \ **Anode**	*Case 1*: NiFe@NF	*Case 2*: 5-stacked SSMs
Case 1: Ru@Cu-TM	Tested	Tested
Case 2: Ru@Cu-Ti@NM	Tested *(Zirfon Perl UTP 500+)*	Not tested
Case 3: Ru@Cu-Ti@SSM	Not tested	Best case scenario – Not tested

Supplementary Note 1. Comments on PGM-based electrocatalysts for HER

The most performant electrocatalysts for the HER in alkaline media still often rely on PGMs,[33,34] with Pt deposited on carbonaceous supports (Pt/C) being widely reported as the state-of-the-art catalyst in AELs and KOH-fed AEM-ELs.[35,36] Typical Pt mass loadings in Pt/C catalysts (~600 µg/cm^2 of Pt)[18] obviously result in prohibitive costs in perspective of massive use. Thus, current AEL plant make use of PGM-free electrodes (e.g., Ni), despite their inferior catalytic activity compared to state-of-the-art catalysts. Undoubtedly, several progresses have been recently accomplished in the field of Pt-free catalysts for the HER in alkaline media,[37,38] daily raising the bar of the performance expectations. Nevertheless, investigations are often restricted to maximum current densities of ~100 mA/cm^2 in 1 M KOH, moving away from the operating condition of desired AELs (e.g., hundreds/thousands of mA/cm^2 and 20-30 wt% KOH electrolytes). Also, the electrocatalysts durability is often investigated for limited time, mostly less than 24 h.[39,40]

Supplementary Note 2. Comments on comparison between the water splitting performances of our AELs and those of ELs

A recent work reported a PGM-based AEM-EL based on a poly(fluorenyl-co-aryl piperidinium) (PFAP) AEM that achieved a current density of 7.68 A/cm^2 at 2 V in 1 M KOH at 80 °C.[36] However, despite a claimed durability of 1000 h, after few hours, it operated at only 0.5 A/cm^2 with a cell voltage higher than 2 V, indicating a non-durable performance.[36] In the same work, the PGM-free AEM-EL based on same PFAP AEM operated at 1.6 A/cm^2 at 2 V, showing a performance inferior to that recorded for our AELs (whose low Ru amount has a non-relevant impact on the capital expense -CAPEX- of our AELs, as shown by our technoeconomic analysis). Another impacting work reported an AEM-ELs based on PGM-free anode and RuPt cathode that operated at 5.3 A/cm^2 at 1.8 V, but also in this case the stability at 0.2 A/cm^2 was limited to only few hours.[34]

Supplementary Note 3. Comments on the different trends of performance of SSM and NiFe@NF as anode in three-electrode configuration and AEL conditions

Our best AELs have been realized using SSM-based anodes that were not the most performing ones in the three-electrode characterization tests (**Supplementary Fig. 17g**). This could be due to: 1) under industrial-level current densities (1 A/cm^2 in our case), the NiFe compounds grown on NF may lose Fe species more quickly due to its higher surface area contacted to electrolyte, in relative to the NiFe species continuously produced by dealloying/oxidation of SSMs,[41,42] and thus reducing the catalytic activity; 2) cell assembly and perturbation from those strongly evolved O_2 bubbles in operation could detach/break the structure of the NiFe species grown on NF surface (**Supplementary Fig. 31b**); 3) 5-stacked SSM could decrease the “real” current density applied to it, if compared to a single SSM, thereby alleviating/preventing Fe etching.

Supplementary References

1. Shinde, D. V. *et al.* A robust and highly active hydrogen evolution catalyst based on Ru nanocrystals supported on vertically oriented Cu nanoplates. *J. Mater. Chem. A* **8**, 10787–10795 (2020).
2. Xie, C. *et al.* In-situ phase transition of WO3 boosting electron and hydrogen transfer for enhancing hydrogen evolution on Pt. *Nano Energy* **71**, 104653 (2020).
3. Chen, W. *et al.* Deciphering the alternating synergy between interlayer Pt single-atom and NiFe layered double hydroxide for overall water splitting. *Energy Environ. Sci.* **14**, 6428–6440 (2021).
4. Shinde, D. V. *et al.* In Situ Dynamic Nanostructuring of the Cu-Ti Catalyst-Support System Promotes Hydrogen Evolution under Alkaline Conditions. *ACS Appl. Mater. Interfaces* **10**, 29583–29592 (2018).
5. Feng, J. X. *et al.* Efficient Hydrogen Evolution Electrocatalysis Using Cobalt Nanotubes Decorated with Titanium Dioxide Nanodots. *Angew. Chemie - Int. Ed.* **56**, 2960–2964 (2017).
6. Alexander, C. S. & Pritchard, J. Chemisorption of hydrogen on evaporated copper films. *J. Chem. Soc. Faraday Trans. 1 Phys. Chem. Condens. Phases* **68**, 202–215 (1972).
7. Kraemer, K. & Menzel, D. Adsorption of Gases on Ruthenium Field Emitters II. Hydrogen. *Berichte der Bunsengesellschaft für Phys. Chemie* **78**, 728–733 (1974).
8. Nørskov, J. K. *et al.* Trends in the Exchange Current for Hydrogen Evolution. *J. Electrochem. Soc.* **152**, J23 (2005).
9. Liu, Y. *et al.* Corrosion engineering towards efficient oxygen evolution electrodes with stable catalytic activity for over 6000 hours. *Nat. Commun.* **9**, 2609 (2018).
10. Yin, H. *et al.* Remarkably enhanced water splitting activity of nickel foam due to simple immersion in a ferric nitrate solution. *Nano Res.* **11**, 3959–3971 (2018).
11. Etzi Coller Pascuzzi, M., Man, A. J. W., Goryachev, A., Hofmann, J. P. & Hensen, E. J. M. Investigation of the stability of NiFe-(oxy)hydroxide anodes in alkaline water electrolysis under industrially relevant conditions. *Catal. Sci. Technol.* **10**, 5593–5601 (2020).
12. Tian, X., Liu, Y., Xiao, D. & Sun, J. Ultrafast and large scale preparation of superior catalyst for oxygen evolution reaction. *J. Power Sources* **365**, 320–326 (2017).
13. Lu, X. & Zhao, C. Electrodeposition of hierarchically structured three-dimensional nickel-iron electrodes for efficient oxygen evolution at high current densities. *Nat. Commun.* **6**, 6616 (2015).
14. Liu, Z. *et al.* The effect of membrane on an alkaline water electrolyzer. *Int. J. Hydrogen Energy* **42**, 29661–29665 (2017).
15. Tian, M. *et al.* Influence of the Working and Counter Electrode Surface Area Ratios on the Dissolution of Platinum under Electrochemical Conditions. *ACS Catal.* **6**, 5108–5116 (2016).
16. Cui, Z. & Sheng, W. Thoughts about Choosing a Proper Counter Electrode. *ACS Catal.* **13**, 2534–2541 (2023).
17. Möller, S. *et al.* Online Monitoring of Electrochemical Carbon Corrosion in Alkaline Electrolytes by Differential Electrochemical Mass Spectrometry. *Angew. Chemie - Int. Ed.* **59**, 1585–1589 (2020).
18. Kang, Z., Alia, S. M., Young, J. L. & Bender, G. Effects of various parameters of

different porous transport layers in proton exchange membrane water electrolysis. *Electrochim. Acta* **354**, 136641 (2020).

19. de Groot, M. T. & Vreman, A. W. Ohmic resistance in zero gap alkaline electrolysis with a Zirfon diaphragm. *Electrochim. Acta* **369**, 137684 (2021).
20. Härtinger, S., Pettinger, B. & Doblhofer, K. Cathodic formation of a hydroxyde adsorbate on copper (111) electrodes in alkaline electrolyte. *J. Electroanal. Chem.* **397**, 335–338 (1995).
21. Shiddiky, M. J. A., O'Mullane, A. P., Zhang, J., Burke, L. D. & Bond, A. M. Large amplitude fourier transformed AC voltammetric investigation of the active state electrochemistry of a copper/aqueous base interface and implications for electrocatalysis. *Langmuir* **27**, 10302–10311 (2011).
22. Lyu, X. *et al.* Investigation of oxygen evolution reaction with Ni foam and stainless-steel mesh electrodes in alkaline seawater electrolysis. *J. Environ. Chem. Eng.* **10**, 108486 (2022).
23. Moureaux, F., Stevens, P., Toussaint, G. & Chatenet, M. Timely-activated 316L stainless steel: A low cost, durable and active electrode for oxygen evolution reaction in concentrated alkaline environments. *Appl. Catal. B Environ.* **258**, 117963 (2019).
24. Zappia, M. I. *et al.* High-current density alkaline electrolyzers: The role of Nafion binder content in the catalyst coatings and techno-economic analysis. *Front. Chem.* **10**, 1362 (2022).
25. Gannon, W. J. F. & Dunnill, C. W. Raney Nickel 2.0: Development of a high-performance bifunctional electrocatalyst. *Electrochim. Acta* **322**, 134687 (2019).
26. Dotan, H. *et al.* Decoupled hydrogen and oxygen evolution by a two-step electrochemical–chemical cycle for efficient overall water splitting. *Nat. Energy* **4**, 786–795 (2019).
27. Zhu, Y. *et al.* Unusual synergistic effect in layered Ruddlesden−Popper oxide enables ultrafast hydrogen evolution. *Nat. Commun.* **10**, 149 (2019).
28. IRENA. *Green Hydrogen Cost Reduction: Scaling up Electrolysers to Meet the 1.5^{0}C Climate Goal*. (2020).
29. https://www.fuelcellstore.com/alkaline-electrolysis-stack-1-cell. (accessed June 11, 2023)
30. Lee, B. *et al.* Integrative techno-economic and environmental assessment for green H2 production by alkaline water electrolysis based on experimental data. *J. Environ. Chem. Eng.* **9**, 106349 (2021).
31. Zimbro, M. J., Power, D. A., Miller, S. M., Wilson, G. E. & Johnson, J. A. Difco & BBL Manual: Manual of Microbiological Culture Media. *Citeseer* **40**, 289 (2009).
32. Yates, J. *et al.* Techno-economic Analysis of Hydrogen Electrolysis from Off-Grid Stand-Alone Photovoltaics Incorporating Uncertainty Analysis. *Cell Reports Phys. Sci.* **1**, 100209 (2020).
33. McCrum, I. T. & Koper, M. T. M. The role of adsorbed hydroxide in hydrogen evolution reaction kinetics on modified platinum. *Nat. Energy* **5**, 891–899 (2020).
34. Li, D. *et al.* Highly quaternized polystyrene ionomers for high performance anion exchange membrane water electrolysers. *Nat. Energy* **5**, 378–385 (2020).
35. Kraglund, M. R. *et al.* Ion-solvating membranes as a new approach towards high rate alkaline electrolyzers. *Energy Environ. Sci.* **12**, 3313–3318 (2019).
36. Chen, N. *et al.* High-performance anion exchange membrane water electrolyzers with a

current density of 7.68 A cm-2and a durability of 1000 hours. *Energy Environ. Sci.* **14**, 6338–6348 (2021).

37. Li, Y. *et al.* Implanting Ni-O-VOx sites into Cu-doped Ni for low-overpotential alkaline hydrogen evolution. *Nat. Commun.* **11**, 2720 (2020).
38. Li, Z. *et al.* Stable Rhodium (IV) Oxide for Alkaline Hydrogen Evolution Reaction. *Adv. Mater.* **32**, 1908521 (2020).
39. Lei, C. *et al.* Efficient alkaline hydrogen evolution on atomically dispersed Ni-N x Species anchored porous carbon with embedded Ni nanoparticles by accelerating water dissociation kinetics. *Energy Environ. Sci.* **12**, 149–156 (2019).
40. Fang, S. *et al.* Uncovering near-free platinum single-atom dynamics during electrochemical hydrogen evolution reaction. *Nat. Commun.* **11**, 1029 (2020).
41. Todoroki, N. & Wadayama, T. Electrochemical stability of stainless-steel-made anode for alkaline water electrolysis: Surface catalyst nanostructures and oxygen evolution overpotentials under applying potential cycle loading. *Electrochem. commun.* **122**, 106902 (2021).
42. Todoroki, N. & Wadayama, T. Heterolayered Ni-Fe Hydroxide/Oxide Nanostructures Generated on a Stainless-Steel Substrate for Efficient Alkaline Water Splitting. *ACS Appl. Mater. Interfaces* **11**, 44161–44169 (2019).
