# Supplementary material for: High-performance alkaline water electrolyzers based on Ru-perturbed Cu nanoplatelets cathode
Source: Nat Commun. 2023 Aug 4;14:4680. doi: 10.1038/s41467-023-40319-5 (PMC10403570; doi:10.1038/s41467-023-40319-5)
Supplement: Supplementary file 2 — Description of Additional Supplementary Files [file 41467_2023_40319_MOESM2_ESM.pdf]

Description of Additional Supplementary Files

Supplementary Data 1: Techno-economic analyses performed on our developed Ru@Cu-TM cathode, coupling the stacked stainless steel anode, to estimate the CAPEX, OPEX and H_2 production cost of a single cell and of a corresponding ideal 1 MW-scale AEL plant. All calculations were run for each cathode-anode combination, depending on the data availability shown in Supplementary Table 13. More details could be found in the Supplementary Information file.

Supplementary Data 2: Techno-economic analyses carried out for a commercial Ni-based single cell AEL (Alkaline Electrolysis Stack, 12 cm^2 active size - Fuel Cell Corp.), whose performances are reported in Supplementary Fig. 44. Following our TEA approach on Ru-based AEL, we have set a desired plant power, which is equivalent to setting a fixed amount of current - energy - OPEX. More details could be found in the Supplementary Information file.
